# When do stereotypes undermine indirect reciprocity?

**DOI:** 10.1371/journal.pcbi.1011862

**Published:** 2024-03-01

**Authors:** Mari Kawakatsu, Sebastián Michel-Mata, Taylor A. Kessinger, Corina E. Tarnita, Joshua B. Plotkin

**Affiliations:** 1 Department of Biology, University of Pennsylvania, Philadelphia, Pennsylvania, United States of America; 2 Center for Mathematical Biology, University of Pennsylvania, Philadelphia, Pennsylvania, United States of America; 3 Department of Ecology and Evolutionary Biology, Princeton University, Princeton, New Jersey, United States of America; Dartmouth College, UNITED STATES

## Abstract

Social reputations provide a powerful mechanism to stimulate human cooperation, but observing individual reputations can be cognitively costly. To ease this burden, people may rely on proxies such as stereotypes, or generalized reputations assigned to groups. Such stereotypes are less accurate than individual reputations, and so they could disrupt the positive feedback between altruistic behavior and social standing, undermining cooperation. How do stereotypes impact cooperation by indirect reciprocity? We develop a theoretical model of group-structured populations in which individuals are assigned either individual reputations based on their own actions or stereotyped reputations based on their groups’ behavior. We find that using stereotypes can produce either more or less cooperation than using individual reputations, depending on how widely reputations are shared. Deleterious outcomes can arise when individuals adapt their propensity to stereotype. Stereotyping behavior can spread and can be difficult to displace, even when it compromises collective cooperation and even though it makes a population vulnerable to invasion by defectors. We discuss the implications of our results for the prevalence of stereotyping and for reputation-based cooperation in structured populations.

## Author summary

Someone who behaves altruistically can gain a good social standing, which makes others more likely to help them in the future. This feedback between altruistic behavior and reputation, called indirect reciprocity, is a key mechanism for promoting cooperation in large societies. However, keeping track of each individual’s social standing may be difficult. To reduce the mental burden, people sometimes rely on proxies, such as stereotypes based on group affiliations. But how do stereotypes affect reputation-based cooperation? We study this problem using a mathematical model in which individuals are judged either by their individual reputation, based on their own behavior, or by their stereotyped reputation, based on the behavior of their group. Using stereotypes can facilitate or impede cooperation, depending on how people share their views of one another. When individuals adjust their behavior through social learning, stereotype usage tends to spread, and it can persist at high levels even when it is detrimental to cooperation. This study helps us understand the spread of stereotyping behavior and its downstream impact on reputations and cooperation in populations with group structure.

## Introduction

Reputations are key to maintaining cooperation in large human societies [1–4]. When someone is observed behaving altruistically, their reputation improves, and others are disposed to help them in the future. This feedback loop, termed indirect reciprocity, can be a strong motivator and mechanism for cooperation [2, 5–8]. Theoretical models of indirect reciprocity have found that cooperation depends on the extent to which individuals share the same views of one another. When reputations are shared, public knowledge [9–11]—facilitated by gossip [2, 12, 13] or by institutions that broadcast information [14, 15]—everyone agrees about each other’s reputation, and cooperation thrives as individuals choose to cooperate with those of good social standing. However, when individuals hold private and thus possibly different opinions about the moral standings of others, disagreements about reputations can lead to the perception of unjustified behavior, a decay of reputations, and the eventual collapse of cooperation [16–19].

In reality, however, people may not use individual reputations when judging each other but instead rely on proxies such as social identities. Coarser reputations assigned to social groups rather than to individuals can be thought of as stereotypes. Social psychology defines stereotypes in various ways; here we adopt the widely accepted view that stereotypes are beliefs about the characteristics of members of certain social groups [20–22], which can be positive or negative. Stereotypes are readily accessible because societies are organized into groups based on factors such as culture, language, wealth, or political affiliations. Stereotypes are also cognitively inexpensive because they provide mental shortcuts that are easily learnable [23, 24]. However, stereotyped reputations present a drawback for indirect reciprocity: stereotypes are less accurate than individual-level reputations, so they might disrupt the positive feedback between altruistic behavior and social standing, thereby undermining cooperation. This raises an important question: what happens to the maintenance of cooperation via indirect reciprocity when people rely, at least to some extent, on stereotyped reputations?

Here we tackle this question by necessarily embedding it into a broader study of information sharing in group-structured populations. Group structure can impact reputation dynamics in two distinct ways. First, group structure allows for the possibility of stereotyped reputations that are assigned to entire groups rather than to individuals. Second, group structure also allows for intermediate scales of information dissemination. For instance, members within a group may agree on their opinions of others, but different groups may hold different views. Both of these effects remain understudied, with a few notable exceptions [15, 25, 26]. In this context, we can break down our main question into three interrelated questions. What matters more for the degree of collective cooperation achieved via indirect reciprocity: the scale at which reputation information is shared across the population (as existing results on public versus private information would suggest) or the granularity of reputation assessment itself (individual versus stereotyped reputations)? Will the tendency to use stereotypes spread across a population, and if so, can stereotyping behavior be dislodged once it has spread? Finally, how does stereotyping affect the stability of cooperation via indirect reciprocity when discriminators compete against strategies that disregard reputations altogether?

**Fig 1:**
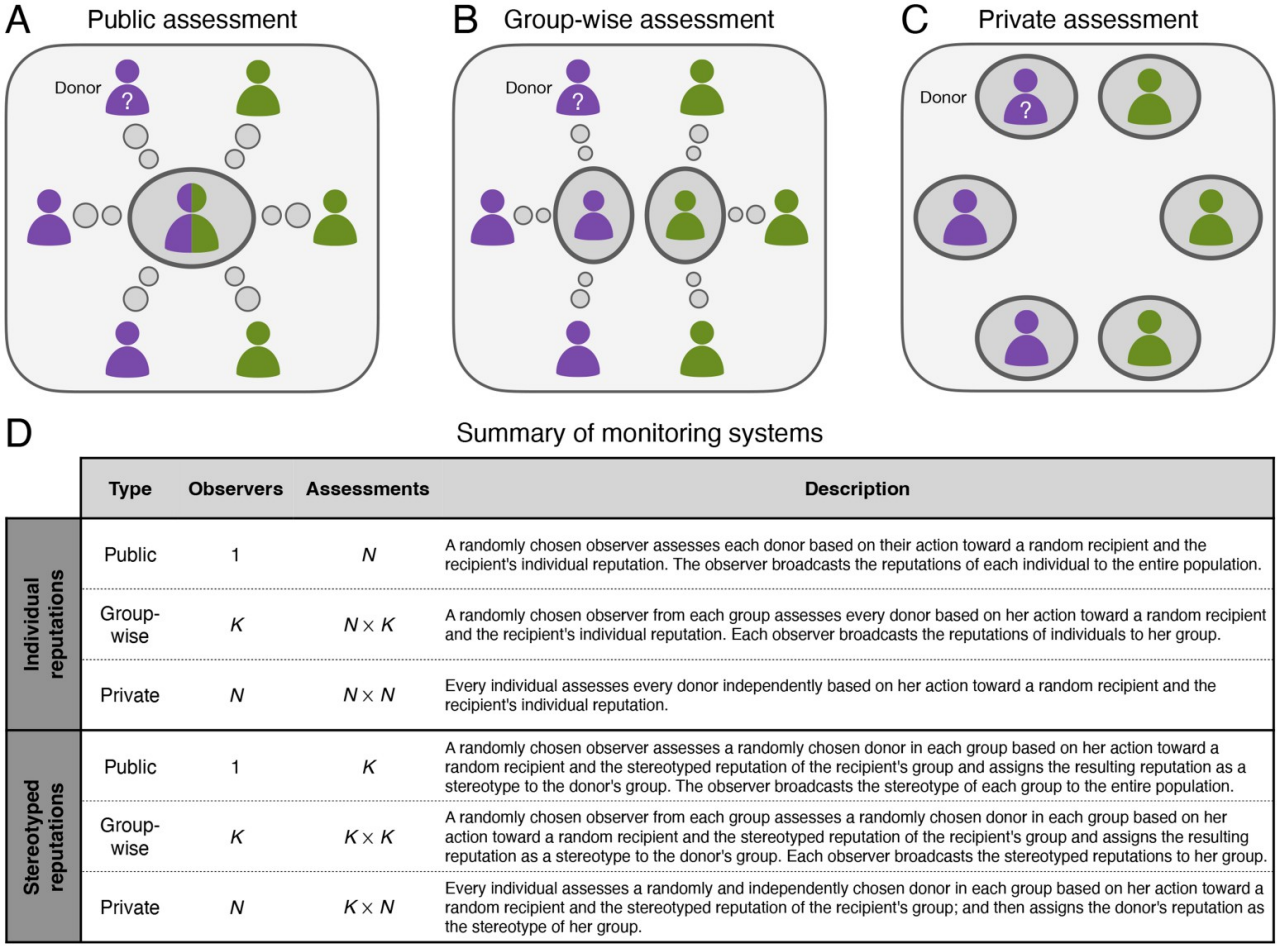
Monitoring systems for individual and stereotyped reputations. A–C: Schematic illustrations of the three monitoring systems. Green and purple denote group memberships. A: Under public assessment, a single observer (who can belong to either the green or purple group; dark gray oval) broadcasts the individual reputation of each donor (or the stereotyped reputation of each group) to the entire population (following the small circles). B: Under group-wise assessment, an observer from each group (dark gray ovals) broadcasts the individual reputation of each donor (or the stereotyped reputation of each group) to her group (following the small circles). C: Under private assessment, individuals hold their own views of each donor (or each group). D: Summary of the number of observers and total number of assessments under the three monitoring systems considered, for either individual or stereotyped reputations.

To study these questions, we extend a game-theoretic model of indirect reciprocity in populations with group structure [15] in two ways. First, we introduce the possibility of stereotyped reputations—that is, reputations assigned to groups as opposed to individuals. We consider the simplest implementation: to form a stereotyped reputation of a group, an individual observes a single, random member of that group, assesses her reputation, and then ascribes that reputation to everyone in her group. One individual’s altruistic behavior can thus improve not only her own reputation but also the reputation of the group to which she belongs, potentially benefiting members of her in-group. Second, we introduce the possibility that reputation information, whether individual or stereotyped, is shared at different scales (Fig 1): individual, group-wise, or public. When information is held individually, two individuals, even from the same group, may disagree about someone else’s reputation. Group-wise sharing of reputation information ensures agreement within groups but allows for disagreement between groups. Lastly, public sharing results in unanimous agreement about everyone’s reputation.

## Model

We consider an infinitely large population structured into *K* non-overlapping groups. We let *ν*_*k*_ denote the fraction of the total population in group *k*, with $\sum_{k=1}^{K}\nu_{k}=1$. For simplicity, we will focus on two groups of equal size (*ν*_1_ = *ν*_2_ = 0.5), but our model can accommodate any number of groups of different sizes.

### Games and behavioral strategies

Individuals engage in pairwise social interactions with everyone in the population, regardless of group membership. Each interaction takes the form of a one-shot donation game, which provides a minimal model of a social dilemma. In each game, the *donor* must choose whether or not to cooperate by paying a *cost c* > 0 to provide a *benefit b* > *c* > 0 to the *recipient*. If the donor defects, she incurs no cost and provides no benefit.

Whether a donor cooperates or not depends on her current strategy. We consider three strategies commonly explored in game-theoretic models of reputations [27–29]: always cooperate (ALLC), which means the donor intends to cooperate with any recipient; always defect (ALLD), which means the donor defects against any recipient; and discriminate (*p*DISC), which means the donor intends to cooperate when the donor considers the recipient as good but defects when the donor considers the recipient as bad. We allow for errors in strategy execution [18, 19, 27, 29]: with probability 0 ≤ *u*_*e*_ ≤ 1/2 (*execution error rate*), a donor erroneously defects while intending to cooperate.

The *stereotype-use propensity p* associated with strategy *p*DISC modulates the type of information a donor uses to judge the recipient as good or bad. With probability 1 − *p*, a donor uses the recipient’s *individual* reputation, as in traditional models of indirect reciprocity [2, 10, 27, 28]. With probability *p*, the donor uses the recipient’s *stereotyped* reputation, i.e., the donor’s view of the entire group to which the recipient belongs. We describe below how reputations are updated over time (Reputations and monitoring systems).

Assessing and recalling an individual’s reputation may carry a higher cognitive cost than simply using the stereotyped reputation of the group to which the individual belongs [23]. To model this effect, we assume that a *p*DISC donor pays an *access cost η* ≥ 0 per interaction when using the individual reputation of the recipient but pays no such cost when using the stereotyped reputation of the recipient’s group.

### Strategy dynamics

We describe the dynamics of competing strategies using replicator differential equations [30]. As is common in the literature on indirect reciprocity [14, 15, 27, 29], we assume that the timescale of reputation updates is faster than that of strategy dynamics, so that reputations (individual or stereotyped) equilibrate before individuals consider updating their strategies: Every individual interacts pairwise with every other individual in each round of social interactions, acting once as a donor and once as a recipient. After all pairwise games are completed, reputations are updated according to a monitoring system and a social norm, described below. Strategy frequencies then change in the population at rates proportional to their relative payoffs. We assume global imitation: individuals can imitate anyone in the population, not just those in their groups.

We let $f_{i}^{I}$ be the frequency of strategy *i* in group *I*. Under the assumption of global imitation, comparison partners are chosen irrespective of group membership, and as a result, strategy frequencies $f_{i}^{I}$ will quickly equalize across groups *I* and converge to a common frequency *f*_*i*_. The dynamics of strategy frequencies then follow the equation (see [15] for the derivation),

$$\begin{array}{c} {\dot{f}}_{i}=f_{i}\sum_{J}\nu_{J}(\Pi_{i}^{J}-{\bar{\Pi }}^{J})\;, \end{array}$$ (1)

where $\Pi_{i}^{J}$ is the fitness of strategy *i* in group *J* (derived from game payoffs described above and defined mathematically in Eq (2) in Materials and methods) and ${\bar{\Pi }}^{J}=\sum_{j}f_{j}^{J}\Pi_{j}^{J}$ is the average fitness of group *J*.

### Social norms

A given observer assesses a donor according to a prescribed social norm, or a set of rules that determine how the donor’s reputation (good or bad) depends on her behavior towards a third-party recipient [2, 10, 11, 19, 28]. We consider the four second-order norms, which depend on the donor’s action and the recipient’s reputation, that are most common in the literature [27, 29]: Stern Judging, Simple Standing, Shunning, and Scoring (see Materials and methods for definitions). While more complex norms are possible, they typically produce less cooperation than the simple norms we consider [28].

We allow for errors in reputation assessment [18, 19, 27, 29]. With probability 0 ≤ *u*_*a*_ ≤ 1/2 (*assessment error rate*), an observer erroneously assigns a good reputation instead of a bad reputation, or vice versa.

### Reputations and monitoring systems

In a population with group structure, reputation information can be shared at different scales. We define *monitoring systems* for reputations that specify how reputations are shared within the population. Aside from the commonly studied cases of public [2, 11, 14, 28] or private [16, 18, 19, 27] monitoring systems, we also study a *group-wise* monitoring system [15, 25] in which members within a group agree on their views of others, but there might be disagreement between groups. Altogether, we consider three scales of information sharing, summarized in Fig 1:

1. *Public*: There is a single public view of each individual or group (Fig 1A).
2. *Group-wise*: Each group has a common view of each individual or group (Fig 1B).
3. *Private*: Each individual has a private view of each individual or group (Fig 1C).

To study the effects of stereotyping on cooperation, we consider two types of reputations (Fig 1D). An individual reputation is assigned to each donor by assessing her action toward a randomly chosen recipient according to the prescribed social norm. By contrast, a stereotyped reputation is assigned to an entire group by assessing the behavior of one randomly chosen donor from that group. If the selected member is assessed as good (bad), then the stereotyped assessment of her entire group is good (bad).

Individual and stereotyped reputations can operate at different scales of information sharing, leading to nine possible combinations of monitoring systems. For example, reputations about individuals may be held privately, whereas stereotyped reputations about groups may be held group-wise. As a concrete example, members of an academic department might disagree on their views of individual colleagues (private individual reputations) but collectively subscribe to the stereotype that their department is good and another department is bad (group-wise stereotyped reputations).

## Results

### The scale of information sharing matters more for cooperation than the granularity of reputation information

To address the first of the three questions outlined in the Introduction, we study the joint effects of the granularity of information (stereotyped reputations) and the scale of information sharing (monitoring system) on cooperation. To do so, we first consider a population of discriminators (*p*DISC) with a fixed, uniform propensity *p* ∈ [0, 1] to use stereotypes instead of individual reputations. This analysis allows us to isolate the effect of information sharing (the nine monitoring systems described above and in Fig 1) and stereotyping propensity (*p*) on the equilibrium behavior and payoffs among discriminators. The equilibrium cooperation levels described below are thus independent of the payoff parameters, *b* and *c*, and the cost of accessing individual reputations, *η*.

**Fig 2:**
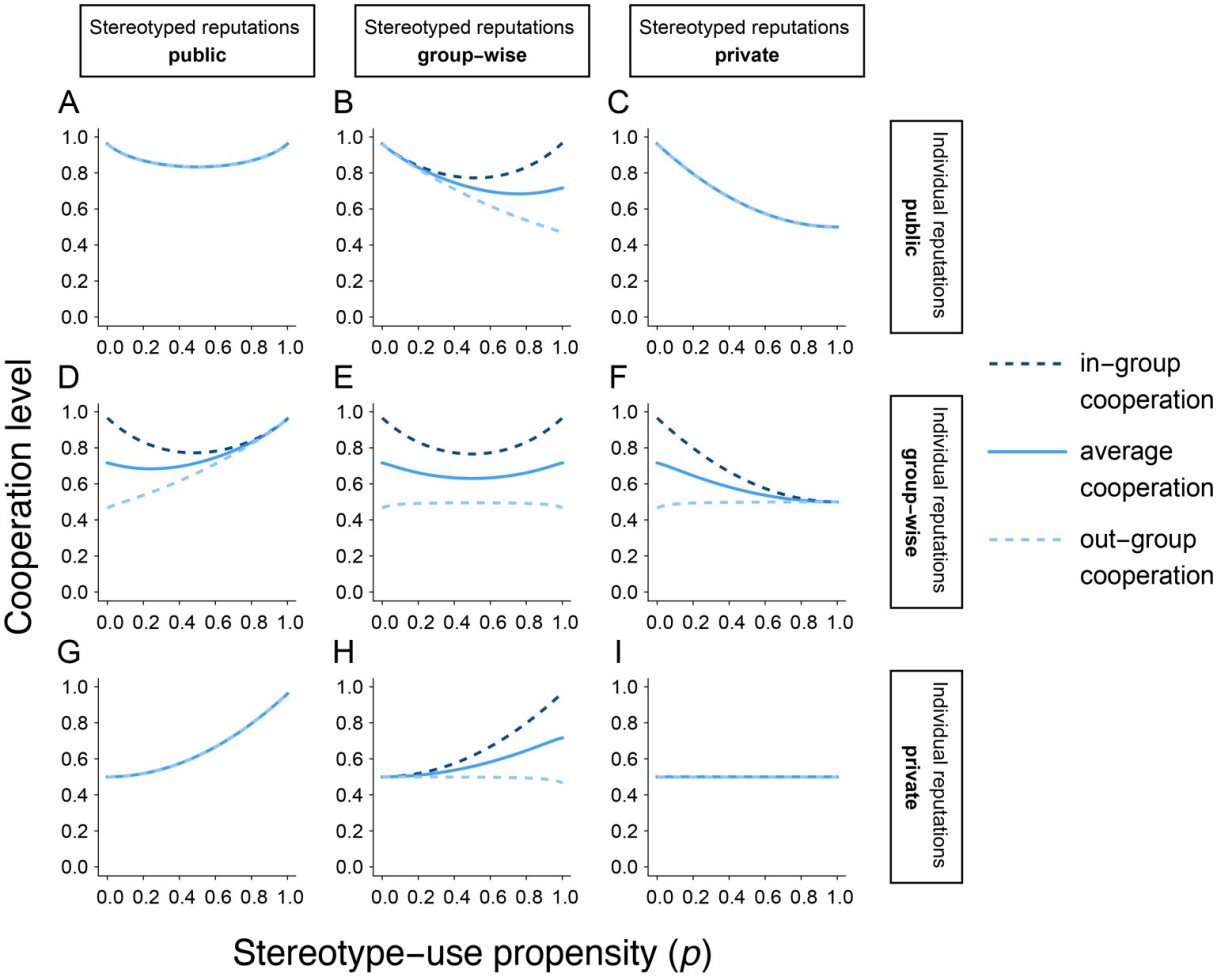
Stereotyping can produce high or low levels of cooperation depending on the scale of information sharing. A-I: We analyzed the equilibrium level of cooperation among *p*DISC strategists under the Stern Judging norm, as a function of the propensity *p* to use stereotypes instead of individual reputations. The population consists of two groups of equal size (*K* = 2, *ν*_1_ = *ν*_2_ = 0.5). Each panel shows a combination of monitoring systems for individual (row) and stereotyped (column) reputations. Solid lines show the average cooperation levels across the entire population; dashed lines show average cooperation levels within (dark blue) and between (light blue) groups. The gap between in- and out-group cooperation is most pronounced when individuals use only individual reputations (*p* = 0; B, E, and F) or only stereotypes (*p* = 1; D, E, and H). Error rates are *u*_*a*_ = *u*_*e*_ = 0.02. Analogous results for the Scoring, Shunning, and Simple Standing norms are shown in S1–S3 Figs. Analytical results corresponding to the case in panel A are provided in Section 3.2 of S1 Text.

The highest rates of cooperation occur when individuals exclusively use either stereotyped (*p* = 1) or individual (*p* = 0) reputations (Fig 2). But whether full stereotyping (*p* = 1) or no stereotyping (*p* = 0) leads to the highest cooperation levels depends on whether individual or stereotyped information is broadcast more widely. If individual information is shared more widely than stereotyped information, then cooperation is maximized when the population uses only individual information (*p* = 0; Fig 2B, 2C and 2F); conversely, if stereotyped information is more widely shared than individual information, then cooperation is maximized when the population always uses stereotypes (*p* = 1; Fig 2D, 2G and 2H). If individual and stereotyped reputations are both shared group-wise or both shared publicly, then cooperation is equally maximized at both *p* = 0 and *p* = 1 (Fig 2A and 2E). These results highlight that, in general, a discriminating population is most cooperative when everyone uses individual assessment or everyone uses stereotypes; but which particular solution is best depends on how information is shared. The only exception is when both types of reputations are held privately, in which case the cooperation level is independent of stereotype propensity *p* (Fig 2I). These qualitative patterns also hold for norms other than Stern Judging (S1–S3 Figs): the level of cooperation is maximized at *p* = 0 or *p* = 1 (S1(A)–S1(G) and S2(A)–S2(G) Figs), unless it is independent of *p* (S1(I), S2(I) and S3(A)–S3(I) Figs).

These results reflect a trade-off between precision and disagreement under each monitoring system. Stereotyped reputations are less precise than individual reputations because the former rely on the behavior of a single randomly sampled donor. However, stereotypes can reduce disagreements about reputations and thereby stimulate cooperation when they are shared more broadly than individual reputations. In other words, the answer to the first of the three questions in the Introduction is that the scale of information sharing trumps the granularity of reputations in its effect on collective cooperation.

The degree of collective cooperation is one important metric; however, when a population is partitioned into groups, one can also measure the degree of within- versus between-group cooperation. An unexpected outcome of our model setup is that group-wise monitoring gives rise to in-group favoritism—that is, individuals cooperate preferentially with members of their own group—a phenomenon that does not occur under public or private monitoring (Fig 2B, 2D–2F and 2H; versus Fig 2A, 2C, 2G and 2I). This is an emergent phenomenon: we have assumed no behavioral strategies with cooperation conditioned on group memberships. Rather, group-wise monitoring allows for different levels of agreement within and between groups, which, in turn, produce differential rates of in- and out-group cooperation. Interestingly, the stereotyping level(s) *p* that maximizes in-group relative to out-group cooperation can also sometimes maximize collective cooperation (Fig 2E, 2F and 2H), similar to phenomena in models of cooperation in polarized populations [31].

In-group favoritism is particularly strong under Stern Judging relative to the other three social norms (Fig 2 versus S1–S3 Figs). This is likely because the Stern Judging norm harshly punishes discrepancies in assessment. Prior literature has found that Stern Judging is the most effective norm for promoting cooperation under indirect reciprocity [28] and is naturally favored when norms compete [15, 32]. Therefore, we will hereafter focus our analysis on Stern Judging, although our mathematical formulation can be used to study all four norms (Materials and methods). We leave detailed analyses of the norms other than Stern Judging, as well as comparisons across norms, for future work.

### Stereotyping behavior can spread and be stable even when it reduces collective cooperation

Under Stern Judging, each combination of monitoring systems has an optimal level (or levels) of stereotype usage that maximizes collective cooperation in populations of discriminators (Fig 2). However, it remains unclear whether the degree of stereotyping that is best for the collective payoff will actually evolve in a population.

To address this question, we use the framework of adaptive dynamics [33] to study the spread of stereotyping propensity. We consider a resident population with a given propensity to use stereotypes, *p*_*R*_, and we analyze whether a rare mutant with a slightly different propensity, *p*_*Q*_, will have higher fitness and invade. We restrict our analysis to discriminators (*p*DISC), and we let the stereotype propensity *p* gradually change in the direction that increases payoff. We define Π_*Q*_ and Π_*R*_ as the per-round expected payoff of the resident and invader types with stereotype propensities 0 < *p*_*R*_, *p*_*Q*_ < 1, respectively. We derive an analytic expression for the invasion fitness Π_*Q*_ − Π_*R*_ in the limit of negligible invader frequency (Eq (9) in Materials and methods), and we evaluate this expression numerically across a range of model parameters (S4 Fig). To determine long-term population dynamics, we identify singular points 0 < p* < 1 and characterize their stability (Materials and methods).

Adaptive dynamics in stereotyping propensity (*p*) do not always lead to the collective optimum payoff. For example, under public stereotyped reputations assessed according to the Stern Judging norm and with *η* = 0.3, the maximum collective fitness is achieved when individuals always use stereotypes (*p* = 1; S6(A) and S6(D) Fig), but adaptive dynamics predict that *p* will approach a unique stable equilibrium at *p* = 0 (S4(A) and S4(D) Fig). Monte Carlo simulations in finite populations confirm these predictions (S5(A) and S5(D) Fig).

More generally, the dynamic trajectory of stereotype propensity depends on the monitoring systems for individual and stereotyped reputations. Under Stern Judging, when all information is public, the only attractor is no stereotyping (*p* = 0; S4(A) Fig). When all information is held either group-wise or privately, maximum stereotype use (*p* = 1) is one possible attractor (S4(E) and S4(I) Fig). However, under group-wise monitoring, there is another, repulsive singular point at intermediate *p* that produces bistability (S4(E) Fig): if the resident population starts with *p*_*R*_ below this value, then the population will eventually use only individual reputations (*p* = 0), whereas a population starting from above this value will eventually adopt complete stereotyping (*p* = 1). Stochastic simulations in finite populations show agreement with this analysis (S5 Fig).

We can systematically identify how model parameters impact the dynamics of stereotype propensity. Parameters that tend to promote high stereotype usage include a high cost to access individual reputations (S7 Fig); a low benefit-to-cost ratio of cooperation (S7 Fig); and high error rates in assessment and execution (S8 Fig). Each of these model parameters can move the system from regimes in which the only stable outcome is complete reliance on individual reputations (*p* = 0) to regimes in which the only stable outcome is complete reliance on stereotypes (*p* = 1). In between these extremes, there are intermediate regimes featuring bistability, so that the long-term outcome for *p* depends on initial conditions. These results have intuitive explanations in terms of a cost-precision trade-off between individual and stereotyped reputations (S7 and S8 Figs).

We have shown that stereotype use can sometimes spread by adaptive dynamics, especially when individual reputations are costly to access or when strategy execution and reputation judgments are prone to errors. Eliminating these conditions might reduce the use of stereotypes. However, it is unclear whether a population will always benefit from curbing the use of stereotypes in this model, because stereotyping can be either beneficial or harmful to cooperation (Fig 2). When will the spread of stereotyping behavior be beneficial to collective cooperation in the population? And how might a population transition from full stereotyping to no stereotyping?

**Fig 3:**
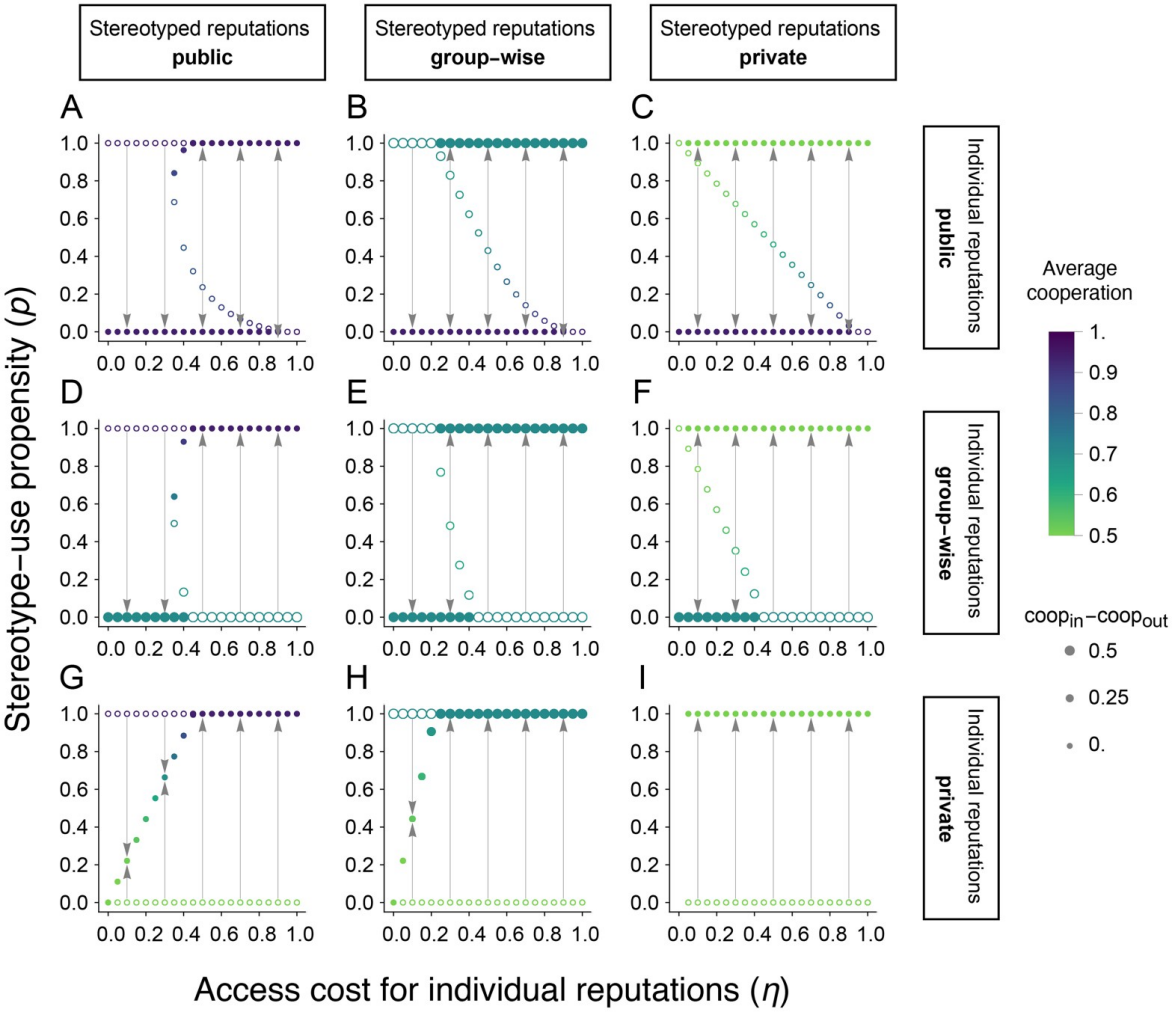
The spread of stereotyping behavior and its effects on cooperation. We analyzed the stability of singular points (p*) and extreme values for the adaptive dynamics of stereotyping propensity, *p*, as a function of the cost *η* to access individual reputations. The population consists of two groups of equal size (*K* = 2, *ν*_1_ = *ν*_2_ = 0.5), all using the discriminator strategy; the results remain qualitatively unchanged even when a fixed proportion of the population (20%) uses the defector strategy (S9 Fig). Each panel shows a combination of monitoring systems for individual (row) and stereotyped (column) reputations. Solid (empty) circles denote attractive (repulsive) singular points for *p*. Gray arrows denote the attractive points toward which the population will converge for given values of *η* and initial values of *p*. Colors indicate the average level of cooperation for each singular point (Fig 2). Circle size indicates the difference between in- and out-group cooperation levels; larger sizes indicate larger differences. A–F: When individual reputations are held group-wise or publicly, there are regions of bistability facilitated by backward bifurcations. For intermediate costs *η*, an attractive singular point emerges at a high stereotyping level (e.g., p* ≈ 0.85 at *η* = 0.35 in A). As *η* increases, so does the basin of initial conditions from which the population will tend towards the attractor at *p* > 0. But when *η* is sufficiently high, *p* = 0 becomes unstable, and populations will always converge to high stereotyping levels regardless of initial conditions. G, H: When individual reputations are held privately but stereotypes are not (i.e., held group-wise or publicly), the stable level of stereotyping increases gradually from 0 to 1 with increasing access cost *η*. Intermediate values of *η* lead to stable intermediate equilibria (0 < p* < 1). I: When both types of reputations are private, full stereotyping (*p* = 1) is the only attractor for any cost *η* > 0; stereotyping is neither favored nor disfavored for *η* = 0 (see also S7(I) Fig). Results are shown for the Stern Judging norm. Parameters: *b* = 3, *c* = 1, *u*_*e*_ = *u*_*a*_ = 0.02.

To address these questions, we examine the dynamically attractive values of stereotype propensity *p* under Stern Judging and, for each attractor, we quantify the resulting level of cooperation in a population of discriminators (Fig 3). Our analysis uncovers the possibility of bistability and hysteresis in stereotype usage.

The costlier it is to access individual reputations (higher *η*), the more likely it is for individuals to use stereotypes (higher *p*) (Fig 3, consistent with S7 Fig). However, whether higher access cost *η* improves cooperation or not depends on the monitoring systems. When stereotypes are more widely shared than individual reputations (panels below the diagonal, Fig 3D, 3G and 3H), higher access cost *η* increases cooperation at the corresponding dynamically attractive stereotype rate *p*. In contrast, when stereotypes are less widely shared than reputations (panels above the diagonal, Fig 3B, 3C and 3F), reducing the access cost improves cooperation at the attractor. And when stereotypes and reputations are both monitored at the same scale (private, group-wise, or public), the access cost has little effect on the level of cooperation at attractive values of *p*. (The only exception is the bistable region in Fig 3A, where cooperation dips slightly at p* < 1.) Overall, while the propensity to stereotype tends to increase with the cost of accessing individual reputations, *η*, the resulting levels of cooperation can increase or decrease depending on the scales of information sharing.

Importantly, stereotyping behavior can be ‘sticky’ (Fig 3A–3F) even when individuals can adjust their stereotyping propensities to achieve higher payoffs. Under group-wise or public individual reputations, if a population initially relies solely on stereotypes (*p* = 1), then a small decrease in *η* may not immediately curtail the use of stereotypes, even in regimes where *p* = 0 would be stable and produce higher levels of cooperation. This phenomenon is caused by a large region of bistability in *p* as a function of *η*. (When a shift to individual reputations does occur, however, it will be sudden: for example, we predict a jump from *p* ≈ 0.85 to 0 as *η* crosses 0.35 (Fig 3A).) Hence, stereotyping can reach high levels and thereafter be resistant to displacement, even when parameters change so that the population could stably persist without stereotyping and achieve higher collective payoffs. These results highlight regimes where stereotyping is not only deleterious to a population’s fitness but also difficult to dislodge.

### Stereotyping can destabilize indirect reciprocity

We have shown that stereotyping behavior can persist even when it lowers the level of cooperation in the population. However, our analysis has been restricted to strategic types that condition their donation behavior based on the reputation of the recipient (i.e., discriminators). So the question remains: How will discriminators adapt their stereotyping behavior in the presence of opponents who ignore reputations altogether? And what are the downstream consequences of widespread stereotyping for the stability of cooperation?

To address the first question, we repeated our analysis of how, under the Stern Judging norm, stereotype propensities change as individuals seek higher fitness, assuming now that a fixed proportion of the population behaves as unconditional defectors (S9 Fig). We considered a population consisting of 20% defectors (ALLD), who cannot change strategies, and 80% discriminators (*p*DISC), who can adapt their stereotyping propensity following adaptive dynamics but cannot imitate unconditional defectors. We find that the overall levels of cooperation achieved at the stable levels of stereotyping are lower in the presence of defectors (S9 Fig) than in their absence (Fig 3), which is to be expected. Importantly, though, the long-term outcomes for stereotyping usage *p* are qualitatively similar in both cases: for a large majority of the parameter conditions we considered, the population will adopt either full stereotyping (*p* = 1) or no stereotyping (*p* = 0), with bistable regimes characterizing the transitions between the two states under public or group-wise individual reputations (S9(A)–S9(F) Fig versus Fig 3A–3F). Thus, the dynamics of stereotyping propensity are not significantly changed, even when discriminators compete against a fixed pool of defectors.

**Fig 4:**
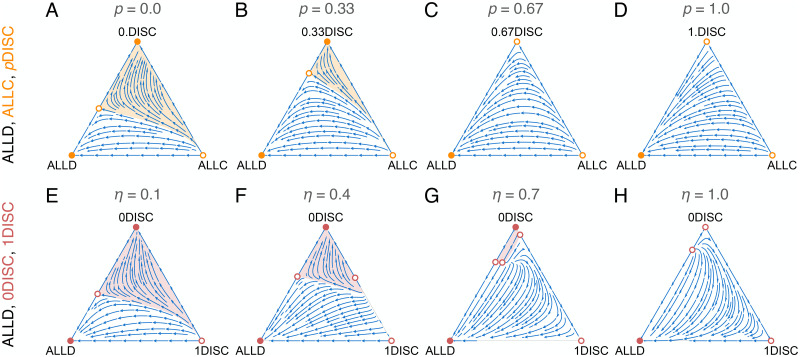
The use of stereotypes can destabilize cooperation. Arrows indicate dynamical flow within the frequency simplex of three competing strategies—ALLD, ALLC, and *p*DISC (A–D), or ALLD, 0DISC, and 1DISC (E–H). Solid (open) circles indicate stable (unstable) equilibria. Individuals are distributed across two groups of equal size (*K* = 2, *ν*_1_ = *ν*_2_ = 0.5). Individual and stereotyped reputations are assessed using the Stern Judging norm and broadcast publicly; in S10 Fig, we study the case with group-wise monitoring, which results in qualitatively similar outcomes. A–D: When discriminators rely solely on individual reputations (*p* = 0; A), there is a large basin of attraction towards a stable non-stereotyping population (0DISC), which produces high rates of cooperation (Fig 2A). As the stereotyping propensity *p* increases (B), the basin of attraction shrinks. When discriminators rely heavily on stereotyped reputations (*p* = 0.67, C; *p* = 1, D), the all-*p*DISC equilibrium loses stability, and the only stable outcome is pure defection. E–H: The basin of attraction towards the *p*DISC vertex is largest when individual reputations are inexpensive (low *η*; E), but it quickly shrinks with increasing *η* (F–H). Discriminators who do not stereotype are robust to invasion by defectors (along the 0DISC-ALLD edge); but, for sufficiently high access cost (H), discriminators who do not stereotype (0DISC) can be invaded by those who do (1DISC), who, in turn, can be invaded by ALLD. Thus, stereotyping strategies serve as a Trojan horse that can dislodge a population from a cooperative state to full defection (ALLD). Results are shown for the Stern Judging norm. Parameters: *b* = 3, *c* = 1, *u*_*e*_ = *u*_*a*_ = 0.02.

To address the second question—the effects of widespread stereotyping on the stability of cooperation—we analyze competition among strategies that do and do not use reputations. For this analysis, we let the strategy frequencies change according to replicator dynamics (Model). That is, individuals imitate others’ strategies in order to increase payoffs. Here we focus on the case when individual and stereotyped reputations are fully public information (Fig 4); results qualitatively similar to those below also hold under group-wise information (S10 Fig).

First, we analyzed competition among cooperators (ALLC), defectors (ALLD), and discriminators (*p*DISC) under the Stern Judging norm (Fig 4A–4D). When discriminators use individual reputations (*p* = 0), there is a large basin of attraction towards the all-discriminator equilibrium, which produces a high level of cooperation (Fig 4A). This is a classic result about the value of Stern Judging and public information [15, 18]. As the stereotyping propensity *p* of discriminators increases, however, the basin for the all-discriminator equilibrium shrinks, and the basin for pure defection expands (Fig 4B). When *p* increases further (*p* = 0.67, 1), the all-discriminator equilibrium becomes unstable altogether, and the only stable outcome is pure defection (Fig 4C and 4D); in fact, regardless of *η*, the *p*DISC equilibrium is locally unstable when *p* = 1 (Sections 3 and 4 in S1 Text). In sum, high levels of stereotyping among discriminators destabilize cooperation when individuals can choose to imitate ALLD or ALLC.

This collapse of cooperation occurs because a population of discriminators who rely on stereotyped reputations is vulnerable to invasion by unconditional defectors. Whether a discriminator who stereotypes will cooperate with a focal individual depends on the stereotyped reputation of the focal individual’s group, which is based on the action of a randomly sampled member of that group rather than the focal individual’s own action. As a result, the focal individual has little incentive to cooperate: her cooperative actions are unlikely to have a positive impact on her social standing in the eyes of heavily stereotyping discriminators. Hence, reliance on stereotyped reputations—even when stereotypes are fully public information—removes the collective benefit of reputations that is otherwise the mainstay of cooperation under the standard theory of indirect reciprocity [2, 10, 11].

We also analyzed competition among defectors (ALLD), non-stereotyping discriminators (0DISC), and full-stereotyping discriminators (1DISC) under Stern Judging (Fig 4E–4H), with different values of the cost to access individual reputations (*η*). When individual reputations are inexpensive (*η* = 0.1), 0DISC has a large basin of attraction (Fig 4A). As the access cost *η* increases, however, non-stereotyping and full-stereotyping strategies become bistable (along the 0DISC–1DISC edge), which diminishes the size of the basin for 0DISC and magnifies the basin for ALLD (Fig 4B and 4C). For a sufficiently high *η*, 1DISC dominates 0DISC, so that the non-stereotyping discriminator (0DISC) is no longer stable and unconditional defection is the only stable outcome (Fig 4D).

These results show that stereotyping can act as a Trojan horse: when discriminators who stereotype (1DISC) enjoy a higher fitness than non-stereotypers (0DISC), the former will increase in frequency, but they will eventually be invaded by unconditional defectors (Fig 4D; Section 2 in S1 Text). This phenomenon occurs even when non-stereotyping discriminators would have been stable against defection in the absence of any stereotyping types. This vulnerability presents a dilemma: when *η* is sufficiently high, full stereotyping (*p* = 1) is the sole dynamic attractor for *p*, and monomorphic populations of discriminators who rely only on stereotypes are highly cooperative (Figs 2A and 3A). But in such populations, the introduction of an unconditional defector will cause cooperation to collapse. A similar tragic outcome can also occur when unconditional cooperators make a population vulnerable to eventual defection, although the mechanism is qualitatively different (S11 Fig).

## Discussion

We have developed a game-theoretic model of cooperative behavior conditioned on two alternative types of reputations, assigned either to individuals or to entire groups. A donor can condition her behavior on either the recipient’s individual reputation, at a cost, or on the stereotyped reputation of the recipient’s group. Such a discriminating donor is characterized by her propensity to use stereotypes. In addition, reputation information (individual or stereotyped) can be shared privately, group-wise, or publicly. This model allows us to study when stereotype usage will spread and how it affects the level of sustained cooperation in a population.

We find that the impact of stereotyping on cooperation depends critically on how widely information is shared. In the context of our model, stereotyping is not always harmful to cooperation: monomorphic populations of conditional cooperators who rely on stereotypes can achieve higher levels of cooperation when stereotypes are shared more broadly than individual reputations. This finding complements previous theoretical and empirical work demonstrating that people tend to cooperate more when they engage in intuitive, rather than deliberative, decision-making [34–36] and that uncalculating cooperation can even elicit trust from others [37].

But when individuals are allowed to adapt their stereotyping propensity, we find a series of deleterious effects. The propensity to use stereotypes can spread when individual reputations are costly to access or when strategy execution and moral assessments are error-prone. Stereotype propensity can also exhibit bistability, such that stereotyping can be sticky and persist at high levels even in populations that would stably benefit without stereotype usage. Finally, high levels of stereotyping can destabilize cooperation, making an otherwise cooperative population vulnerable to invasion by pure defectors.

These findings do not bode well for a world where political groups hold antagonistic stereotypes of one another. In the United States, for example, both Democrats and Republicans dislike members of the opposing party and describe them as “hypocritical, selfish, and closed-minded” [38]. Our results suggest that such affective polarization may result in a triple tragedy: the persistent reliance on group-wise stereotypes can (1) entrench in-group preference, (2) diminish society-wide cooperation, and (3) serve as an intermediary step toward pure defection. Mechanisms to reverse this series of tragedies, even in the context of simple mathematical models, remain an important open question.

Our results raise the question: if stereotypes adversely affect population fitness, under even the most robust and otherwise beneficial social norm [15, 32], then what explains their prevalence in human behavior? One possibility is that stereotypes are common today because of the evolutionary history of population sizes and systems of information sharing. In small-scale societies without a formal mechanism to broadcast information, reputations are effectively private information. In this scenario, our model suggests that the use of stereotypes may be favored, provided there is some (cognitive) cost of tracking individual-level reputations (Fig 3I). But as societies grow in size, observing others’ actions becomes increasingly difficult. This could not only make individual reputations more costly but also give rise to formal institutions that broadcast information, either at the level of the group (group-wise monitoring; Fig 3E) or the whole population (public monitoring; Fig 3A). Our analysis suggests that these two effects—higher access cost for individual reputations and wider dissemination of reputation information—can produce bistability (Fig 3A and 3E), so that high levels of stereotyping persist even in regimes where individual reputations would be more beneficial.

Behavior conditioned on stereotyped reputations is distinct from the notion of tag-based cooperation [39–44] and from the concept of statistical discrimination in economics [45, 46]. There are some similarities between these theories and our results: for example, statistical discrimination theory posits that individuals are more likely to use group-based proxies when individualized information is costly to access [46]. However, there is a key distinction: tag-based cooperation and statistical discrimination concern how tags, which are immutable labels based solely on group affiliation, modulate behavior (e.g., the green beard effect) [39, 42]. By contrast, the stereotyped reputations in our study are generalized assessments based on observed behavior, and they may change from positive to negative over time. Recent work has explored such generalized assessments in the context of direct reciprocity, in repeated games within and between groups of finite size [47]. Our study complements this work by exploring stereotypes in the context of indirect reciprocity, in one-shot games with infinitely large groups. Bridging these two approaches by exploring the effect of relative and absolute group sizes on stereotype formation, particularly in finite populations (e.g. [19]) subject to stochasticity, would be a key step towards a unified understanding of how cognitive heuristics affect cooperation based on reciprocity [48].

We have implemented a minimal model of stereotypes to establish their basic consequences on behavior. As simple as it is, this model shows that coarser social information adds significant nuance to our understanding of reputation-driven cooperation, underscoring the need to extend indirect reciprocity to include stereotyping. Our study thus provides a framework for future research—both theoretical and empirical—that incorporates real-world complexities of stereotyping and studies their effects on cooperative behavior. For example, evidence shows that individuals preferentially recall information consistent with existing stereotypes [22, 49]. This suggests stereotypes may be slower to change than individual reputations: observing a single ‘bad’ behavior by a member of a stereotypically ‘good’ group may not alter the observer’s stereotype of that group. At the same time, recent theoretical work suggests that extrinsic ‘shocks’ can quickly cause positive stereotypes to turn negative [47]. Exploring how relative timescales of updating individual versus stereotyped reputations affect cooperation remains an important area for future research.

Our analysis did not impose any asymmetry between within- and among-group behavior. For example, our model assumes a single rate of stereotyping for both in- and out-group interactions. On the one hand, this restriction allowed us to reveal emergent behavioral asymmetries that arise even without any intrinsic bias for in-group members. Nonetheless, empirical work in social psychology suggests that people may intrinsically judge out-groups as more homogeneous than in-groups (out-group homogeneity) or, occasionally, vice versa (in-group homogeneity), depending on context [50–52]. For instance, members of minority groups may perceive in-group members as being more similar to one another than out-group members [53, 54]. To accommodate this in our model, we would need to incorporate differential rates of stereotyping for in- and out-groups. Moreover, stereotypes are prone to exaggeration [21, 55]; for instance, people tend to view minority groups more negatively than majority groups, even if they behave identically [22]. It remains unclear how such minority biases will affect indirect reciprocity.

Our model also assumes that individuals use a single social norm to assess all individuals and groups, but in reality people may adopt different rules to evaluate in- and out-groups. Previous work has considered combinations of norms in monomorphic populations, with stereotypes applied only to out-groups [25]. However, it remains unclear how variation in norms will affect competition among types who use or do not use stereotypes.

Finally, our study focuses on a simple population structure, where strategic interactions are well-mixed. A natural alternative is that individuals interact more frequently with in-group members than out-group members (or exclusively with in-group members [56]). Recent theoretical work has shown that interaction insularity can boost cooperation under group-wise information sharing [15]. One extension of our analysis could consider the dynamics of stereotyping among individuals who favor in-group social interactions. Moreover, group membership itself may be dynamic and overlapping: even within a lifetime, one can belong to different cultural, familial, or occupational groups at different times. Although we have tools for studying cooperation in temporal social networks [15, 57–59], little is known about the co-evolution of population structure, individual reputations, and stereotypes. These topics remain important directions for future research.

## Materials and methods

Here we provide additional details of our mathematical model (Model). We refer the reader to S1 Text for detailed derivations.

### Fitness

We consider three strategic types, always cooperate (ALLC), always defect (ALLD), and discriminate (*p*DISC), whose behaviors are described in Model (Games and behavioral strategies). The fitness of each strategic type in group *I* is given by

$$\begin{array}{cc} \Pi_{\text{ALLC}}^{I} & =\left(1-u_{e}\right)[b\sum_{J}\nu_{J}(f_{\text{ALLC}}^{J}+f_{p\text{DISC}}^{J}\left[\left(1-p\right)g_{\text{ALLC}}^{I,J}+pg_{S}^{I,J}\right])-c]\;,\\ \Pi_{\text{ALLD}}^{I} & =\left(1-u_{e}\right)[b\sum_{J}\nu_{J}(f_{\text{ALLC}}^{J}+f_{p\text{DISC}}^{J}\left[\left(1-p\right)g_{\text{ALLD}}^{I,J}+pg_{S}^{I,J}\right])]\;,\\ \Pi_{p\text{DISC}}^{I} & =\left(1-u_{e}\right)[b\sum_{J}\nu_{J}(f_{\text{ALLC}}^{J}+f_{p\text{DISC}}^{J}\left[\left(1-p\right)g_{p\text{DISC}}^{I,J}+pg_{S}^{I,J}\right])-c\left[\left(1-p\right)g^{•,I}+pg^{⋆,I}\right]]\\ & -\eta (1-p)\;, \end{array}$$ (2)

where $f_{i}^{I}$ be the frequency of strategy *i* in group *I*; $g_{i}^{I,J}$ is the fraction of strategy *i* individuals in group *I* who have good individual reputations in the eyes of group *J*; $g_{S}^{I,J}$ is the fraction of group *I* who have good stereotyped reputations in the eyes of group *J*; $g^{•,I}=\sum_{J}\nu_{J}\sum_{i}f_{i}^{J}g_{i}^{J,I}$ is the fraction of the whole population who have good individual reputations in the eyes of group *I*; and $g^{⋆,I}=\sum_{J}\nu_{J}g_{S}^{J,I}$ is the fraction of the whole population who have good stereotyped reputations in the eyes of group *I*.

### Social norms

A second-order social norm can be expressed as a binary matrix, with rows indicating the donor’s action (row one for cooperation, two for defection), columns indicating the recipient’s reputation (individual or stereotyped; column one for good, two for bad), and entries indicating how the donor is assessed (good or bad) [29]. We consider four second-order social norms that are most common in studies of indirect reciprocity [14, 15, 27, 29]: Stern Judging $\left(\begin{array}{cc} G & B\\ B & G \end{array}\right)$, Simple Standing $\left(\begin{array}{cc} G & G\\ B & G \end{array}\right)$, Shunning $\left(\begin{array}{cc} G & B\\ B & B \end{array}\right)$, and Scoring $\left(\begin{array}{cc} G & G\\ B & B \end{array}\right)$. For example, under Stern Judging, an observer will endorse (with a good reputation) a donor who either cooperates with a recipient who has a good reputation (in the eyes of the observer) or who defects against a recipient with a bad reputation; but the observer will condemn (with a bad reputation) a donor who cooperates with a bad recipient or who defects against a good recipient. Note that Scoring is a first-order norm that disregards recipient reputation when assessing a donor.

Let *q*_*C*_ (*q*_*D*_) be the probability that cooperating with (defecting against) a bad recipient yields a good standing. Then the norms can be parameterized as (*q*_*C*_, *q*_*D*_): Stern Judging (0, 1), Simple Standing (1, 1), Scoring (1, 0), Shunning (0, 0).

### Reputation dynamics

We assume that reputations equilibrate more quickly than strategies. In other words, the timescale of reputations is faster than that of strategy dynamics [14, 27, 29].

After all games in a round are complete, each observer—specified for each monitoring system in Fig 1—observes an independent, random interaction of each donor (in the case of individual reputations) or a random interaction of a randomly selected donor in each group (in the case of stereotyped reputations). In the former, the observer evaluates each donor according to the social norm and the individual reputation of the recipient; in the latter, the observer applies the norm to the stereotype of the recipient’s group instead.

Computing the equilibrium reputations involves keeping track of observations with different combinations of (a) observer view (does the observer view the recipient as good or bad?) and (b) donor intent (did the donor view the recipient as good (or bad) and therefore intend to cooperate (or defect)?). To facilitate this, we define the following quantities:

$$\begin{array}{c} \begin{array}{cc} P_{GC} & =\left(1-u_{e}\right)\left(1-u_{a}\right)+u_{e}u_{a}\equiv \varepsilon \;,\\ P_{GD} & =u_{a}\;,\\ P_{BC} & =q_{C}\left(\varepsilon -u_{a}\right)+q_{D}\left(1-\varepsilon -u_{a}\right)+u_{a}\;,\\ P_{BD} & =q_{D}\left(1-2u_{a}\right)+u_{a}\;, \end{array} \end{array}$$ (3)

where *P*_*XY*_ is the probability that a donor who intends to *Y* ∈{cooperate (*C*), defect (*D*)} with a recipient viewed as *X* ∈{good (*G*), bad (*B*)} by the observer is assigned a good reputation (individual or stereotyped). For example, consider *P*_*GC*_: a donor who intends to cooperate with a recipient who has a good individual reputation in the eyes of the observer can maintain a good individual reputation when the donor either (i) successfully cooperates (with probability 1 − *u*_*e*_) and is correctly assigned a good individual reputation (with probability 1 − *u*_*a*_), or (ii) erroneously defects (with probability *u*_*e*_) and is erroneously assigned a good individual reputation (with probability *u*_*a*_).

We also define the following terms:

$$\begin{array}{c} \begin{array}{cc} g_{\alpha ,1}^{J,I} & =\sum_{L}\nu_{L}\sum_{i}f_{i}^{L}g_{i}^{L,I}g_{i}^{L,J}\;,\\ g_{\alpha ,2}^{J,I} & =\sum_{L}\nu_{L}g_{S}^{L,J}\sum_{i}f_{i}^{L}g_{i}^{L,I}=\sum_{L}\nu_{L}g^{L,I}g_{S}^{L,J}\;,\\ g_{\alpha ,3}^{J,I} & =\sum_{L}\nu_{L}g_{S}^{L,I}\sum_{i}f_{i}^{L}g_{i}^{L,J}=\sum_{L}\nu_{L}g_{S}^{L,I}g^{L,J}\;,\\ g_{\alpha ,4}^{J,I} & =\sum_{L}\nu_{L}g_{S}^{L,I}g_{S}^{L,J}\;. \end{array} \end{array}$$ (4)

Consider a group *I* observer assessing a group *J* donor. When both the observer and the donor use *individual* reputations, they agree with probability $g_{\alpha ,1}^{J,I}$ that a randomly chosen third individual has a good *individual* reputation. When the donor uses *stereotyped* reputations while the observer uses *individual* reputations, they agree with probability $g_{\alpha ,2}^{J,I}$ that the third individual has a good *individual* reputation. When the donor uses *individual* reputations while the observer uses *stereotyped* reputations, they agree with probability $g_{\alpha ,3}^{J,I}$ that the third individual has a good *stereotyped* reputation. Finally, when both use *stereotyped* reputations, they agree with probability $g_{\alpha ,4}^{J,I}$ that the third individual has a good *stereotyped* reputation.

In S1 Text (Section 1.1), we show that the average individual reputations associated with the three strategies satisfy

$$\begin{array}{c} \begin{array}{cc} g_{\text{ALLC}}^{I,I} & =g_{\text{ALLC}}^{J,I}=g^{•,I}P_{GC}+\left(1-g^{•,I}\right)P_{BC}\;,\\ g_{\text{ALLD}}^{I,I} & =g_{\text{ALLD}}^{J,I}=g^{•,I}P_{GD}+\left(1-g^{•,I}\right)P_{BD}\;,\\ g_{p\text{DISC}}^{J,I} & =\left(1-p\right)[A_{IJ}(g^{•,I}P_{GC}+\left(1-g^{•,I}\right)P_{BD})\\ & \;+\left(1-A_{IJ}\right)(g_{\alpha ,1}^{J,I}P_{GC}+(g^{•,I}-g_{\alpha ,1}^{J,I})P_{GD}+(g^{•,J}-g_{\alpha ,1}^{J,I})P_{BC}+(1-g^{•,I}-g^{•,J}+g_{\alpha ,1}^{J,I})P_{BD})]\\ & \;+p[g_{\alpha ,2}^{J,I}P_{GC}+(g^{•,I}-g_{\alpha ,2}^{J,I})P_{GD}+(g^{⋆,J}-g_{\alpha ,2}^{J,I})P_{BC}+(1-g^{•,I}-g^{⋆,J}+g_{\alpha ,2}^{J,I})P_{BD}]\;, \end{array} \end{array}$$ (5)

with

$$\begin{array}{c} \begin{array}{cc} A_{IJ} & =\{\begin{array}{cc} 0 & \text{for}\;\text{private}\;\text{individual}\;\text{reputations}\;,\\ \delta_{IJ} & \text{for}\;\text{group-wise}\;\text{individual}\;\text{reputations}\;,\\ 1 & \text{for}\;\text{public}\;\text{individual}\;\text{reputations}\;. \end{array} \end{array} \end{array}$$ (6)

where *δ*_*IJ*_ = 1 if *I* = *J* and 0 if *I* ≠ *J*. We also show (Section 1.2 in S1 Text) that the average stereotyped reputations satisfy

$$\begin{array}{c} \begin{array}{cc} g_{S}^{J,I} & =f_{\text{ALLC}}(g^{⋆,I}P_{GC}+\left(1-g^{⋆,I}\right)P_{BC})+f_{\text{ALLD}}(g^{⋆,I}P_{GD}+\left(1-g^{⋆,I}\right)P_{BD})\\ & \;+f_{p\text{DISC}}\{(1-p)[g_{\alpha ,3}^{J,I}P_{GC}+(g^{⋆,I}-g_{\alpha ,3}^{J,I})P_{GD}+(g^{•,J}-g_{\alpha ,3}^{J,I})P_{BC}+(1-g^{⋆,I}-g^{•,J}+g_{\alpha ,3}^{J,I})P_{BD}]\\ & \;\;+p[\left(1-B_{IJ}\right)(g_{\alpha ,4}^{J,I}P_{GC}+(g^{⋆,I}-g_{\alpha ,4}^{J,I})P_{GD}+(g^{⋆,J}-g_{\alpha ,4}^{J,I})P_{BC}+(1-g^{⋆,I}-g^{⋆,J}+g_{\alpha ,4}^{J,I})P_{BD})\\ & \;\;\;+B_{IJ}(g^{⋆,I}P_{GC}+\left(1-g^{⋆,I}\right)P_{BD})]\}\;, \end{array} \end{array}$$ (7)

with

$$\begin{array}{c} \begin{array}{cc} B_{IJ} & =\{\begin{array}{cc} 0 & \text{for}\;\text{private}\;\text{stereotyped}\;\text{reputations}\;,\\ \delta_{IJ} & \text{for}\;\text{group-wise}\;\text{stereotyped}\;\text{reputations}\;,\\ 1 & \text{for}\;\text{public}\;\text{stereotyped}\;\text{reputations}\;. \end{array} \end{array} \end{array}$$ (8)

### Pairwise invasibility analysis

To determine the level(s) of stereotyping that are dynamically attractive, we use the framework of adaptive dynamics [33] and perform pairwise invasibility analysis in *p*. That is, we investigate which invaders *p*_*Q*_DISC (with stereotyping probability 0 ≤ *p*_*Q*_ ≤ 1) can invade a given resident population *p*_*R*_DISC (with stereotyping propensity 0 ≤ *p*_*R*_ ≤ 1).

Let *f*_*Q*_ and *f*_*R*_ be the frequencies of *p*_*Q*_DISC and *p*_*R*_DISC individuals in the population, respectively. The replicator dynamics for ${\dot{f}}_{Q}$ is given by Eq (1) with *j* ∈ {*Q*, *R*}. To determine when *p*_*Q*_DISC can invade *p*_*R*_DISC, we check whether the gradient is positive when *p*_*Q*_DISC is rare. That is, *p*_*Q*_DISC will invade resident *p*_*R*_DISC if and only if

$$\begin{array}{c} \frac{\partial {\dot{f}}_{Q}}{\partial f_{Q}}{|}_{f_{Q}=0}=\sum_{J}\nu_{J}(\Pi_{Q}^{J}-\Pi_{R}^{J}){|}_{f_{Q}=0}>0\;. \end{array}$$ (9)

See Section 2 in S1 Text for the derivation.

### Stochastic simulations

We perform stochastic simulations in finite populations of *N* = 50 discriminators (*p*DISC). We assume that, initially, all individuals are characterized by a single stereotyping use propensity *p*, but allow for subsequent variation in *p* arising from the stochastic updating. Both individual and stereotyped reputations are initialized randomly, i.e., each is either good or bad with equal probability. All individuals in a given simulation follow the same prescribed social norm and adhere to the prescribed monitoring systems for reputations (Model).

In a generation, individuals undergo multiple rounds of games and reputation updates. A round consists of two steps: First, every individual interacts with everyone in the population (including herself), once as a donor and once as a recipient. Second, all reputations are updated according to the monitoring systems; for simplicity, we assume all updates within a round occur synchronously. These steps are repeated over 2,500 rounds; that is, within a generation, every individual plays 2,500 games with *N* = 50 individuals, for a total of 125,000 pairwise games. This ensures that reputations equilibrate sufficiently before strategy updating, approximating the time-scale separation assumed in the numerical treatment.

Strategy updating follows a pairwise comparison process. After all rounds in a generation are complete, we compute payoff *π*_*i*_ for each individual, with a fixed benefit *b* and cost *c* of cooperation as well as a fixed access cost *η* of using reputations. Here we use per-generation average payoff (i.e., cumulative payoff across 100 games in a generation, averaged over generations), a scaled version of the per-game average payoff used in the numerical treatment. Then, 5 random pairs are chosen from the population. Within each pair *i* and *j*, *j* adopts *i*’s strategy with probability 1/(1 + exp{−*w*(*π*_*i*_ − *π*_*j*_)}); parameter *w* denotes the intensity of selection [60], which captures the impact of the game payoffs on relative success.

The population is also subject to recurring local mutations in *p*. In each generation, the stereotype use propensity *p* of a randomly selected individual changes by some Δ*p* with probability *u*_*s*_ = 10/*N* = 0.2. The deviation Δ*p* is sampled from a normal distribution with mean 0 and standard deviation 0.05.

## Supporting information

S1 Text Supplementary analysis. (PDF)

S1 Table Model parameters and variables. (PDF)

S1 Fig Cooperation levels in monomorphic populations of *p*DISC (Simple Standing). As in Fig 2, but under the Simple Standing norm.
(PDF)

S2 Fig Cooperation levels in monomorphic populations of *p*DISC (Shunning). As in Fig 2, but under the Shunning norm.
(PDF)

S3 Fig Cooperation levels in monomorphic populations of *p*DISC (Scoring). As in Fig 2, but under the Scoring norm.
(PDF)

S4 Fig Pairwise invasibility of *p*DISC strategies. We use adaptive dynamics to predict the dynamics of stereotype-use propensity *p* under the Stern Judging norm. Pairwise invasibility plots indicate parameter regions in which *p*_*Q*_ can invade *p*_*R*_ (white), i.e., invader payoff Π_*Q*_ exceeds resident payoff Π_*R*_ in the limit of negligible invader frequency, or not (black) (Pairwise invasibility analysis in Materials and methods). Each panel shows a combination of monitoring systems for individual reputations (rows) and stereotyped reputations (columns). Orange arrows indicate predicted dynamics of *p* over time. Payoff parameters are *b* = 3, *c* = 1, and *η* = 0.3; error rates are *u*_*a*_ = *u*_*e*_ = 0.02.
(PDF)

S5 Fig Stochastic dynamics of stereotype-use propensity under adaptive dynamics. Stochastic simulations under the Stern Judging norm in finite populations of *N* = 50 with small, local mutations (Stochastic simulations in Materials and methods) support the predictions based on adaptive dynamics (S4 Fig), with rare exceptions (C: three simulation runs starting from *p* = 0.7 go to *p* = 0; E: one simulation run starting from *p* = 0.5 goes to *p* = 0) likely due to mutations moving the population above or below the singular value. Lines indicate mean stereotype-use propensity *p* in the population over time. Colors distinguish initial conditions (monomorphic populations with uniform *p*), with 10 simulation runs per initial condition. Data are sampled every 100 time steps. Each panel shows a combination of monitoring systems for individual reputations (rows) and stereotyped reputations (columns).
(PDF)

S6 Fig Fitness in monomorphic populations. We analyzed individual fitness levels under the Stern Judging norm among *p*DISC strategists with a uniform stereotype-use propensity *p*. As in Fig 2, individuals are in two groups of equal size (*K* = 2, *ν*_1_ = *ν*_2_ = 0.5). Each panel shows a combination of monitoring systems for individual (row) and stereotyped (column) reputations. Color indicates access cost *η*. Parameters: *b* = 3, *c* = 1, *u*_*a*_ = *u*_*e*_ = 0.02.
(PDF)

S7 Fig A lower benefit of cooperation and costly individual reputations promote the use of stereotypes. We show the number and type of dynamically attractive values of *p* under the Stern Judging norm as a function of benefit of cooperation (*b*) and access cost for individual reputations (*η*). Individuals are distributed across two groups of equal size (*K* = 2, *ν*_1_ = *ν*_2_ = 0.5). Each panel shows a combination of monitoring systems for individual (rows) and stereotyped (columns) reputations. Light gray means stereotype use does not spread by adaptive dynamics (*p* = 0 in the only stable outcome). Hues of purple mean stereotype use will spread (p* > 0 is the only stable outcome). Hues of orange mean bistability (*p* = 0 and p* > 0 are both stable outcomes), i.e., stereotype use may spread depending on initial conditions. Parameters: *c* = 1, *u*_*e*_ = *u*_*a*_ = 0.02. **Decreasing the benefit *b* of cooperation promotes stereotyping**: A lower value of *b* increases the relative cost of accessing individual reputations, thus making stereotypes more beneficial. As a result, given a fixed *η*, decreasing *b* shifts the system from a regime that does not support stereotyping (light gray regions in A–F) through bistable regimes (light and dark orange regions in A–F), to regimes with a single attractive point (light and dark purple regions in A–F) in which stereotyping persists in the population in the long term regardless of initial conditions. Although a small benefit of cooperation generally promotes stereotyping, there is one exception: under private individual reputations, the long-term outcome is independent of *b* (G–I). Individuals gain a benefit *b* when donors view them as having good reputations and, therefore, cooperate with them (Model). Under private monitoring, invader and resident individuals have identical individual reputations on average, because two private observers’ assessments are uncorrelated. As a result, for any value of *b*, residents and invaders receive equal amounts of cooperation. Therefore, changing *b* has no impact on their relative fitness and, consequently, on the long-term outcomes for stereotype propensity in this setting. **Increasing the cost *η* of individual reputations promotes stereotyping**: In general, *p* = 0 is the unique attractor when *η* is low, but *p* = 1 is the attractor when *η* is high. The only exception is when both types of reputations are private, where *p* = 1 is the only attractor for any *η* > 0; stereotyping is neither favored nor disfavored for *η* = 0 (I; see also Fig 3I). These results can be understood in terms of a cost-precision trade-off between individual and stereotyped reputations. Individual reputations are more costly to use than stereotypes, but they are also more precise indicators each individual’s standing because, in our model, the stereotype of a group results from the assessment of a randomly sampled individual in that group. However, a sufficiently high *η* exceeds the benefit provided by increased reputational precision, tipping the balance towards favoring stereotyping.
(PDF)

S8 Fig Errors in reputation assessment and strategy execution promote the use of stereotypes. As in S7 Fig, but with varying rates of assessment (*u*_*a*_) and execution (*u*_*e*_) errors. Errors in assessment are more harmful to individual reputations than for stereotyped reputations, because each assessment introduces the possibility of an erroneous judgment. A single observation is used to assign a stereotype to a group of *N*/*K* individuals, whereas *N*/*K* observations are required to assign individual reputations to each member of the group. This means that stereotyping confers a roughly *N*/*K*-fold decrease in the expected number of evaluation errors. Errors in strategy execution also have more negative consequences under individual reputations than under stereotypes. A donor who defects erroneously is more likely to get a bad individual reputation, at least under Stern Judging, which makes others less likely to cooperate with her. However, if the donor is part of a group with a good stereotype, she may still be seen as good. And so relying on stereotypes can help mitigate the vicious cycle of bad reputations and reduced cooperation that is initiated by erroneous actions or judgments. Results are shown for the Stern Judging norm, as in S7 Fig. Parameters: *b* = 3, *c* = 1, *η* = 0.3.
(PDF)

S9 Fig The spread of stereotyping behavior in a population with a fixed proportion of defectors (ALLD). We repeat the analysis by adaptive dynamics as in Fig 3 but in a population in which 20% of individuals are unconditional defectors (ALLD) who do not change strategies. Each panel shows a combination of monitoring systems for individual (row) and stereotyped (column) reputations. Solid (empty) circles denote attractive (repulsive) singular points for *p*. Gray arrows denote the attractive points toward which the population converges for given values of *η* and initial values of *p*. Colors indicate the average level of cooperation for each singular point (Fig 2). Circle size indicates the difference between in- and out-group cooperation levels; larger sizes indicate larger differences. Qualitatively, the outcomes for *p* are similar to the case without ALLD in the population (Fig 3); in particular, in a majority of the parameter conditions studied here, the population will adopt either full stereotyping (*p* = 1) or no stereotyping (*p* = 0) in the long term. Quantitatively, the levels of cooperation achieved at the singular points are lower than in the absence of ALLD (Fig 3). Results are shown for the Stern Judging norm, as in Fig 3. Parameters: *b* = 3, *c* = 1, *u*_*e*_ = *u*_*a*_ = 0.02.
(PDF)

S10 Fig The use of stereotypes can destabilize cooperation (group-wise monitoring). As in Fig 4, but with group-wise monitoring for both individual and stereotyped reputations. The outcomes for both sets of strategies (A–D: ALLD, ALLC and in *p*DISC; E–H: ALLD, 0DISC, and 1DISC) are qualitatively similar to the corresponding results under public monitoring (Fig 4). Results are shown for the Stern Judging norm, as in Fig 4.
(PDF)

S11 Fig Competition among unconditional defectors (ALLD), unconditional cooperators (ALLC), and discriminators who stereotype (0DISC). Just as stereotyping discriminators and tag-based cooperators can destabilize cooperation (Fig 4 and S12 Fig), unconditional cooperators can also make a population vulnerable to defection when individual reputations are costly. However, the mechanism by which the latter creates a pathway toward defection is qualitatively different. To demonstrate this, we analyze competition among cooperators (ALLC), defectors (ALLD), and non-stereotyping discriminators (0DISC), but with varying access cost *η*. Arrows indicate the dynamical flow within the simplex of three competing strategies. All reputations are public information assessed according to the Stern Judging norm. Individuals are distributed across two groups of equal size (*K* = 2, *ν*_1_ = *ν*_2_ = 0.5). When individual reputations are inexpensive (low *η*), there is a large basin of attraction toward either the 0DISC vertex (A) or a stable mixed equilibrium with ALLC and 0DISC (B), each of which can sustain high levels of cooperation. However, this cooperative basin disappears as soon as *η* is high enough such that the ALLC-0DISC equilibrium becomes unstable (C), after which defection is the only stable outcome (C–F). Hence, unlike in the presence of stereotyping, where the cooperative basin shrinks gradually (Fig 4), increasing access cost leads to a discontinuous loss of cooperation in the absence of stereotyping. Parameters: *b* = 3, *c* = 1, *u*_*e*_ = *u*_*a*_ = 0.02. In addition to *η* = 0.1, 0.4, 0.7, 1.0 used in Fig 4 and S10 Fig, we also show *η* = 0.2 and *η* = 0.3 to highlight the details of the transition from cases in which cooperation is sustained (A, B) to those in which cooperation is not sustained (C–F).
(PDF)

S12 Fig Competition among discriminators who stereotype (1DISC), discriminators who do not stereotype (0DISC), and players who cooperate only with the in-group (TAG). Arrows indicate the dynamical flow within the simplex of three competing strategies. All reputations are public information assessed according to the Stern Judging norm. Individuals are distributed across two groups of equal size (*K* = 2, *ν*_1_ = *ν*_2_ = 0.5). TAG exhibits behavior qualitatively similar to ALLD when competing with 0DISC and 1DISC (A–D versus Fig 4E–4H). The basin of attraction towards the 0DISC vertex, which produces high levels of cooperation (Fig 2), is largest when individual reputations are inexpensive (low *η*; A), but it quickly shrinks with increasing *η* (B–D). For sufficiently high access cost (D), 0DISC can be invaded not only by TAG but also by 1DISC, which, in turn, can be invaded by TAG—so that pure tribalism (TAG) is the only stable outcome. Parameters: *b* = 3, *c* = 1, *u*_*e*_ = *u*_*a*_ = 0.02.
(PDF)
