# Supplementary material for: When do stereotypes undermine indirect reciprocity?
Source: PLoS Comput Biol. 2024 Mar 1;20(3):e1011862. doi: 10.1371/journal.pcbi.1011862 (PMC10906830; doi:10.1371/journal.pcbi.1011862)
Supplement: S1 Text — (PDF) [file pcbi.1011862.s001.pdf]

Supporting Information for

When do stereotypes undermine indirect reciprocity?

Mari Kawakatsu[1,2,*,†], Sebastián Michel-Mata[3,*], Taylor A. Kessinger[1],
Corina E. Tarnita[3], and Joshua B. Plotkin[1,2]

ORCiDs: M.K.: 0000-0003-2072-6653, S.M.-M.: 0000-0003-3424-6669, T.A.K.: 0000-0001-7311-9414,
C.E.T.: 0000-0003-4476-0012, J.B.P.: 0000-0003-2349-6304
[1]Department of Biology, University of Pennsylvania, Philadelphia, PA 19104, USA
[2]Department of Ecology and Evolutionary Biology, Princeton University, Princeton, NJ 08544, USA
[3]Center for Mathematical Biology, University of Pennsylvania, Philadelphia, PA 19104, USA
[*]These authors contributed equally.
[†]Corresponding author: M.K.: marikawa@sas.upenn.edu

Contents

1 Reputation dynamics **2**
1.1 Individual reputations . . . 2
1.2 Stereotyped reputations . . . 4

2 Pairwise invasibility analysis **5**
2.1 Two types of discriminators (p_QDISC and p_RDISC) . . . 5
2.2 Non-stereotyping (0DISC) and full-stereotyping (1DISC) discriminators . . . 6
2.3 Non-stereotyping discriminators (0DISC) and unconditional defectors (ALLD) . . . 6
2.4 Full-stereotyping discriminators (1DISC) and unconditional defectors (ALLD) . . . 7

3 Stability analysis **8**
3.1 Equilibrium reputations at the all-pDISC equilibrium under public monitoring . . . 8
3.2 Cooperation levels at the all-pDISC equilibrium under public monitoring . . . 8
3.3 Stability of the all-pDISC equilibrium under public monitoring . . . 9

4 Special case: symmetric groups with $u_e = 0$ **10**

1 Reputation dynamics

Here we derive the equilibrium reputations reported in Materials and Methods. First, recall the following quantities:

$$\begin{aligned}
P_{GC} &= (1-u_e)(1-u_a) + u_e u_a \equiv \varepsilon \ , \\
P_{GD} &= u_a \ , \\
P_{BC} &= q_C(\varepsilon - u_a) + q_D(1-\varepsilon-u_a) + u_a \ , \\
P_{BD} &= q_D(1-2u_a) + u_a \ ,
\end{aligned}$$

where P_{XY} is the probability that a donor who intends to $Y \in$\{cooperate (C), defect (D)\} with a recipient viewed as $X \in$\{good (G), bad (B)\} by the observer is assigned a good reputation (individual or stereotyped).

1.1 Individual reputations

Individual reputations of cooperators. A cooperator (ALLC) gains a good individual reputation by either

- interacting with someone with a good individual reputation (prob. $g^{\bullet,I}$), intending to cooperate, and successfully being assigned a good individual reputation (prob. P_{GC}), or
- interacting with someone with a bad individual reputation (prob. $1-g^{\bullet,I}$), intending to cooperate, and erroneously being assigned a good individual reputation (prob. P_{BC}).

Thus, the average individual reputation for cooperators is given by

$$g^{I,I}_{\text{ALLC}} = g^{J,I}_{\text{ALLC}} = g^{\bullet,I} P_{GC} + (1-g^{\bullet,I}) P_{BC} \ .$$

Individual reputations of defectors. Similarly, a defector (ALLD) gains a good individual reputation by either

- interacting with someone with a good individual reputation (prob. $g^{\bullet,I}$), intending to defect, and erroneously being assigned a good individual reputation (prob. P_{GD}), or
- interacting with someone with a bad individual reputation (prob. $1-g^{\bullet,I}$), intending to defect, and successfully being assigned a good individual reputation (prob. P_{BD}).

Thus, the average individual reputation for cooperators is given by

$$g^{I,I}_{\text{ALLD}} = g^{J,I}_{\text{ALLD}} = g^{\bullet,I} P_{GD} + (1-g^{\bullet,I}) P_{BD} \ .$$

Individual reputations of discriminators. Throughout the following, we assume, without loss of generality, that the observer is in group I, the donor in group J, and the recipient in group L.

A discriminator (pDISC) in group I with probability p of using stereotypes can gain a good individual reputation by

(i) using individual reputations (prob. $1-p$)

  (a) that are shared between the donor and the observer (i.e., individual reputations are public, or they are group-wise and donor and observer belong to the same group), and

    * interacting with someone with a good individual reputation (prob. $g^{\bullet,I}$), intending to cooperate, and successfully being assigned a good individual reputation (prob. P_{GC}), or
    * interacting with someone with a bad individual reputation (prob. $1-g^{\bullet,I}$), intending to defect, and successfully being assigned a good individual reputation (prob. P_{BC}).

  (b) that are not shared between the donor and the observer (i.e., individual reputations are private, or they are group-wise and donor and observer belong to different groups), and

    * interacting with someone the donor (the focal discriminator) views as good (prob. $g_i^{L,J}$) and the observer views as good (prob. $g_i^{L,I}$), intending to cooperate, and being assigned a good individual reputation (prob. P_{GC}), or
    * interacting with someone the donor views as bad (prob. $1-g_i^{L,J}$) and the observer views as good (prob. $g_i^{L,I}$), intending to defect, and being assigned a good individual reputation (prob. P_{GD}).

* interacting with someone the donor views as good (prob. $g_i^{L,J}$) and the observer views as bad (prob. $1 - g_i^{L,I}$), intending to cooperate, and being assigned a good individual reputation (prob. P_{BC}).
* interacting with someone the donor views as bad (prob. $1 - g_i^{L,J}$) and the observer views as bad (prob. $1 - g_i^{L,I}$), intending to defect, and being assigned a good individual reputation (prob. P_{BD}).

(ii) using stereotyped reputations (prob. p), and

- interacting with someone the donor (the focal discriminator) views as good (prob. $g_S^{L,J}$) and the observer views as good (prob. $g_i^{L,I}$), intending to cooperate, and being assigned a good individual reputation (prob. P_{GC}).
- interacting with someone the donor views as bad (prob. $1 - g_S^{L,J}$) and the observer views as good (prob. $g_i^{L,I}$), intending to defect, and being assigned a good individual reputation (prob. P_{GD}).
- interacting with someone the donor views as good (prob. $g_S^{L,J}$) and the observer views as bad (prob. $1 - g_i^{L,I}$), intending to cooperate, and being assigned a good individual reputation (prob. P_{BC}).
- interacting with someone the donor views as bad (prob. $1 - g_S^{L,J}$) and the observer views as bad (prob. $1 - g_i^{L,I}$), intending to defect, and being assigned a good individual reputation (prob. P_{BD}).

Altogether, a pDISC in group J will gain a good individual reputation

(i)(a) with probability $g_{\text{public}}^{J,I} = g^{\bullet,I} P_{GC} + (1 - g^{\bullet,I}) P_{BD}$, when using individual reputations that are shared;

(i)(b) with probability $g_{\text{private}}^{J,I} = g_{\alpha,1}^{J,I} P_{GC} + g_{\beta,1}^{J,I} P_{GD} + g_{\gamma,1}^{J,I} P_{BC} + g_{\delta,1}^{J,I} P_{BD}$, when using individual reputations that are not shared; or

(ii) with probability $g_{\text{independent}}^{J,I} = g_{\alpha,2}^{J,I} P_{GC} + g_{\beta,2}^{J,I} P_{GD} + g_{\gamma,2}^{J,I} P_{BC} + g_{\delta,2}^{J,I} P_{BD}$, when using stereotyped reputations.

Here we define the following disagreement terms for convenience:

$$
\begin{aligned}
g_{\alpha,1}^{J,I} &= \sum_L \nu_L \sum_i f_i^L g_i^{L,I} g_i^{L,J} \\
g_{\beta,1}^{J,I} &= \sum_L \nu_L \sum_i f_i^L g_i^{L,I} (1 - g_i^{L,J}) = g^{\bullet,I} - g_{\alpha,1}^{J,I} \\
g_{\gamma,1}^{J,I} &= \sum_L \nu_L \sum_i f_i^L (1 - g_i^{L,I}) g_i^{L,J} = g^{\bullet,J} - g_{\alpha,1}^{J,I} \\
g_{\delta,1}^{J,I} &= \sum_L \nu_L \sum_i f_i^L (1 - g_i^{L,I})(1 - g_i^{L,J}) = 1 - g^{\bullet,I} - g^{\bullet,J} + g_{\alpha,1}^{J,I}
\end{aligned}
$$

$$
\begin{aligned}
g_{\alpha,2}^{J,I} &= \sum_L \nu_L g_S^{L,J} \sum_i f_i^L g_i^{L,I} = \sum_L \nu_L g_S^{L,J} g^{L,I} \\
g_{\beta,2}^{J,I} &= \sum_L \nu_L (1 - g_S^{L,J}) \sum_i f_i^L g_i^{L,I} = \sum_L \nu_L (1 - g_S^{L,J}) g^{L,I} = g^{\bullet,I} - g_{\alpha,2}^{J,I} \\
g_{\gamma,2}^{J,I} &= \sum_L \nu_L g_S^{L,J} \sum_i f_i^L (1 - g_i^{L,I}) = \sum_L \nu_L g_S^{L,J} (1 - g^{L,I}) = g^{\star,J} - g_{\alpha,2}^{J,I} \\
g_{\delta,2}^{J,I} &= \sum_L \nu_L (1 - g_S^{L,J}) \sum_i f_i^L (1 - g_i^{L,I}) = \sum_L \nu_L (1 - g_S^{L,J})(1 - g^{L,I}) = 1 - g^{\bullet,I} - g^{\star,J} + g_{\alpha,2}^{J,I}
\end{aligned}
$$

Putting these together, we obtain the average individual reputation of discriminators:

$$
g_{p\text{DISC}}^{J,I} = (1-p) \left[(1 - A_{IJ}) \big(g_{\text{private}}^{J,I} \big) + A_{IJ} \big(g_{\text{public}}^{J,I} \big) \right] + p \left[g_{\text{independent}}^{J,I} \right] ,
$$

where

$$
A_{IJ} = \begin{cases} 0 & \text{for private individual reputations ,} \\ \delta_{IJ} & \text{for group-wise individual reputations ,} \\ 1 & \text{for public individual reputations .} \end{cases}
$$

The version of Eq (1.1) presented in Materials and Methods (Eq (5)) is obtained by substituting the right-most expressions in Section 1.1 into Section 1.1.

1.2 Stereotyped reputations

A cooperator (ALLC) gains a good stereotyped reputation by either

- interacting with someone with a good stereotyped reputation (prob. $g^{\star,I}$), intending to cooperate, and successfully being assigned a good stereotyped reputation (prob. P_{GC}), or
- interacting with someone with a bad stereotyped reputation (prob. $1 - g^{\star,I}$), intending to cooperate, and erroneously being assigned a good stereotyped reputation (prob. P_{BC}).

Thus, a cooperator in group J gains a good stereotyped reputation in the eyes of I with probability

$$g^{J,I}_{S,\text{ALLC}} = g^{\star,I} P_{GC} + (1 - g^{\star,I}) P_{BC} \ .$$

Similarly, a defector in group J gains a good stereotyped reputation in the eyes of I with probability

$$g^{J,I}_{S,\text{ALLD}} = g^{\star,I} P_{GD} + (1 - g^{\star,I}) P_{BD} \ .$$

Finally, analogously to Section 1.1, a discriminator (pDISC) in group J gains a good stereotyped reputation

(iii) with probability $g^{J,I}_{S,\text{independent}} = g^{J,I}_{\alpha,3} P_{GC} + g^{J,I}_{\beta,3} P_{GD} + g^{J,I}_{\gamma,3} P_{BC} + g^{J,I}_{\delta,3} P_{BD}$, when using individual reputations;

(iv)(a) with probability $g^{J,I}_{S,\text{public}} = g^{\star,I} P_{GC} + (1 - g^{\star,I}) P_{BD}$, when using shared stereotyped reputations; or

(iv)(b) with probability $g^{J,I}_{S,\text{private}} = g^{J,I}_{\alpha,4} P_{GC} + g^{J,I}_{\beta,4} P_{GD} + g^{J,I}_{\gamma,4} P_{BC} + g^{J,I}_{\delta,4} P_{BD}$, when using private stereotyped reputations.

Here we define the following disagreement terms for convenience:

$$g^{J,I}_{\alpha,3} = \sum_L \nu_L g^{L,I}_S \sum_i f^L_i g^{L,J}_i = \sum_L \nu_L g^{L,I}_S g^{L,J}$$
$$g^{J,I}_{\beta,3} = \sum_L \nu_L g^{L,I}_S \sum_i f^L_i (1 - g^{L,J}_i) = \sum_L \nu_L g^{L,I}_S (1 - g^{L,J}) = g^{\star,I} - g^{J,I}_{\alpha,3}$$
$$g^{J,I}_{\gamma,3} = \sum_L \nu_L (1 - g^{L,I}_S) \sum_i f^L_i g^{L,J}_i = \sum_L \nu_L (1 - g^{L,I}_S) g^{L,J} = g^{\bullet,J} - g^{J,I}_{\alpha,3}$$
$$g^{J,I}_{\delta,3} = \sum_L \nu_L (1 - g^{L,I}_S) \sum_i f^L_i (1 - g^{L,J}_i) = \sum_L \nu_L (1 - g^{L,I}_S)(1 - g^{L,J}) = 1 - g^{\star,I} - g^{\bullet,J} + g^{J,I}_{\alpha,3},$$

$$g^{J,I}_{\alpha,4} = \sum_L \nu_L g^{L,I}_S g^{L,J}_S$$
$$g^{J,I}_{\beta,4} = \sum_L \nu_L g^{L,I}_S (1 - g^{L,J}_S) = g^{\star,I} - g^{J,I}_{\alpha,4}$$
$$g^{J,I}_{\gamma,4} = \sum_L \nu_L (1 - g^{L,I}_S) g^{L,J}_S = g^{\star,J} - g^{J,I}_{\alpha,4}$$
$$g^{J,I}_{\delta,4} = \sum_L \nu_L (1 - g^{L,I}_S)(1 - g^{L,J}_S) = 1 - g^{\star,I} - g^{\star,J} - g^{J,I}_{\alpha,4}.$$

Finally, the average stereotyped reputation of J in the eyes of I is given by

$$\begin{aligned} g^{J,I}_S &= f_{\text{ALLC}} g^{J,I}_{S,\text{ALLC}} + f_{\text{ALLD}} g^{J,I}_{S,\text{ALLD}} + f_{p\text{DISC}} g^{J,I}_{S,p\text{DISC}} \\ &= f_{\text{ALLC}} \left(g^{\star,I} P_{GC} + (1 - g^{\star,I}) P_{BC} \right) + f_{\text{ALLD}} \left(g^{\star,I} P_{GD} + (1 - g^{\star,I}) P_{BD} \right) \\ &\quad + f_{p\text{DISC}} \left[(1-p) \left[g^{J,I}_{S,\text{independent}} \right] + p \left[(1 - B_{IJ}) \left(g^{J,I}_{S,\text{private}} \right) + B_{IJ} \left(g^{J,I}_{S,\text{public}} \right) \right] \right] \end{aligned}$$

with

$$B_{IJ} = \begin{cases} 0 & \text{private stereotyped reputations ,} \\ \delta_{IJ} & \text{group-wise stereotyped reputations ,} \\ 1 & \text{public stereotypes .} \end{cases}$$

The version of Section 1.2 presented in Materials and Methods (Eq (7)) is obtained by substituting the right-most expressions in Section 1.2 into Section 1.2.

2 Pairwise invasibility analysis

2.1 Two types of discriminators (p_QDISC and p_RDISC)

We now derive the invasibility condition (Eq (9)) reported in Materials and Methods. Recall that our goal is to determine the level(s) of stereotyping that are dynamically attractive. To do so, we use the framework of adaptive dynamics [3] to investigate which invaders p_QDISC (with stereotyping probability $0 \leq p_Q \leq 1$) can invade a given resident population p_RDISC (with stereotyping propensity $0 \leq p_R \leq 1$). Since p is restricted to $p \in [0, 1]$, we also consider whether the extremal values ($p = 0$ and 1) are attractors.

Let f_Q^I and f_R^I be the frequencies of p_QDISC and p_RDISC individuals in group I, respectively, and let f_Q and f_R be their respective frequencies in the full population. The replicator dynamics for $\dot{f}_Q$ is governed by Eq 1 with $j \in \{Q, R\}$. Since $f_Q^I + f_R^I = 1$ for every I, the dynamics of f_Q simplifies to

$$\dot{f}_Q = f_Q \sum_J \nu_J \left(\Pi_Q^J - \bar{\Pi}^J\right) = f_Q \sum_J \nu_J \left(1 - f_Q^J\right) \left(\Pi_Q^J - \Pi_R^J\right) ,$$

where the last equality follows from the fact that $\bar{\Pi}^J = f_Q^J \Pi_Q^J + (1 - f_Q^J)\Pi_R^J$.

To determine when p_QDISC can invade p_RDISC, we first compute the partial derivative of $\dot{f}_Q$ with respect to f_Q, evaluated at $f_Q = 0$. Noting (1) that $\partial f_Q^I / \partial f_Q = (\partial f_Q / \partial f_Q^I)^{-1} = \nu_I^{-1}$ and (2) that $f_Q = 0$ means $f_Q^I = 0$ for all I, we have

$$\begin{aligned}
\left.\frac{\partial \dot{f}_Q}{\partial f_Q}\right|_{f_Q=0} &= \sum_J \nu_J \left(1 - f_Q^J - \nu_J^{-1} f_Q\right) \left(\Pi_Q^J - \Pi_R^J\right) \Big|_{f_Q=0} + f_Q \sum_J \nu_J \left(1 - f_Q^J\right) \left.\frac{\partial(\Pi_Q^J - \Pi_R^J)}{\partial f_Q}\right|_{f_Q=0} \\
&= \sum_J \nu_J \left(\Pi_Q^J - \Pi_R^J\right) \Big|_{f_Q=0}
\end{aligned}$$

Thus, p_QDISC will invade resident p_RDISC if and only if

$$\left.\frac{\partial \dot{f}_Q}{\partial f_Q}\right|_{f_Q=0} = \sum_J \nu_J \left(\Pi_Q^J - \Pi_R^J\right) \Big|_{f_Q=0} > 0 .$$

Based on Eq (2), the average fitness of p_QDISC and p_RDISC in group I are, respectively,

$$\begin{aligned}
\Pi_Q^I = (1 - u_e) \Bigg[b \sum_J \nu_J \Big\{ \Big(f_Q^J (1 - p_Q) + (1 - f_Q^J)(1 - p_R)\Big) g_Q^{I,J} + \Big(f_Q^J p_Q + (1 - f_Q^J) p_R\Big) g_S^{I,J} \Big\} \\
- c \Big((1 - p_Q) g^{\bullet,I} + p_Q g^{\star,I}\Big) \Bigg] - \eta(1 - p_Q) , \\
\Pi_R^I = (1 - u_e) \Bigg[b \sum_J \nu_J \Big\{ \Big(f_Q^J (1 - p_Q) + (1 - f_Q^J)(1 - p_R)\Big) g_R^{I,J} + \Big(f_Q^J p_Q + (1 - f_Q^J) p_R\Big) g_S^{I,J} \Big\} \\
- c \Big((1 - p_R) g^{\bullet,I} + p_R g^{\star,I}\Big) \Bigg] - \eta(1 - p_R) .
\end{aligned}$$

Substituting these expressions into condition (2.1), we can express the invasibility condition as

$$(1 - u_e) \sum_I \nu_I \left[b \sum_J \nu_J \left(1 - p_R\right) \left(g_Q^{I,J} - g_R^{I,J}\right) - c(p_Q - p_R) \left(-g^{\bullet,I} + g^{\star,I}\right) \right] \Bigg|_{f_Q=0} + \eta(p_Q - p_R) > 0 .$$

Reputation dynamics with two types of discriminators. To evaluate the invasibility condition (2.1) further, we need to compute the equilibrium individual and stereotyped reputations, which are obtained by solving the following equations simultaneously:

- A discriminator of the invader type (p_QDISC) in group J has a good individual reputation in the eyes of group I with probability

$$g_Q^{J,I} = (1-p_Q)\left[(1-A_{IJ})\cdot g_{\text{private}}^{J,I} + A_{IJ}\cdot g_{\text{public}}^{J,I}\right] + p_Q\left[g_{\text{independent}}^{J,I}\right] \ ,$$

respectively, where $g_{\text{public}}^{J,I}, g_{\text{private}}^{J,I}, g_{\text{independent}}^{J,I}$ are defined above.

- Similarly, a discriminator of the resident type (p_RDISC) has a good individual reputation in the eyes of group I with probability

$$g_R^{J,I} = (1-p_R)\left[(1-A_{IJ})\cdot g_{\text{private}}^{J,I} + A_{IJ}\cdot g_{\text{public}}^{J,I}\right] + p_R\left[g_{\text{independent}}^{J,I}\right] \ .$$

- Finally, group J has a good stereotyped reputation in the eyes of I with probability

$$\begin{aligned} g_S^{J,I} = &\left[f_Q^J(1-p_Q) + \left(1-f_Q^J\right)(1-p_R)\right]\left[g_{S,\text{independent}}^{J,I}\right] \\ &+ \left[f_Q^J p_Q + \left(1-f_Q^J\right)p_R\right]\left[(1-B_{IJ})\cdot g_{S,\text{private}}^{J,I} + B_{IJ}\cdot g_{S,\text{public}}^{J,I}\right] \ , \end{aligned}$$

where $g_{S,\text{public}}^{J,I}, g_{S,\text{private}}^{J,I}, g_{S,\text{independent}}^{J,I}$ are also defined above.

2.2 Non-stereotyping (0DISC) and full-stereotyping (1DISC) discriminators

0DISC invading 1DISC. Letting $p_R = 1$ and $p_Q = 0$ in condition (2.1), we find that the non-stereotyping (0DISC) discriminator can invade a full-stereotyping (1DISC) resident population provided

$$-c(1-u_e)\sum_I \nu_I\left(g^{\bullet,I} - g^{\star,I}\right)\Big|_{f_Q=0} - \eta > 0 \ .$$

So 0DISC can invade 1DISC when (1) the average *stereotyped* reputation $\sum_I \nu_I g^{\star,I}$ is sufficiently larger than the average *individual* reputation $\sum_I \nu_I g^{\bullet,I}$ and (2) the access cost η is sufficiently low.

1DISC invading 0DISC. Letting $p_R = 0$ and $p_Q = 1$ in condition (2.1), we find that the full-stereotyping (1DISC) discriminator can invade a non-stereotyping (0DISC) resident population provided

$$(1-u_e)\sum_I \nu_I\left[b\sum_J \nu_J\left(g_Q^{I,J} - g_R^{I,J}\right) + c\left(g^{\bullet,I} - g^{\star,I}\right)\right]\Bigg|_{f_Q=0} + \eta > 0 \ .$$

In other words, 1DISC can invade 0DISC when (1) the average individual reputation of the *invader* type $\sum_I\sum_J g_Q^{I,J}$ is sufficiently larger than the average individual reputation of the *resident* type $\sum_I\sum_J g_R^{I,J}$, (2) the average *individual* reputation $\sum_I \nu_I g^{\bullet,I}$ is sufficiently larger than the average *stereotyped* reputation $\sum_I \nu_I g^{\star,I}$, and (3) the access cost η is sufficiently high.

We can also apply condition (2.1) to study whether conditional types can invade unconditional types and vice versa. We focus on the two extreme types of discriminators, 0DISC and 1DISC, and unconditional defectors, ALLD.

2.3 Non-stereotyping discriminators (0DISC) and unconditional defectors (ALLD)

Setting $p = 0$ and $f_{\text{ALLC}} = 0$ in Eq (2), the payoffs of ALLD and 0DISC in group I are, respectively,

$$\begin{aligned} \Pi_{\text{ALLD}}^I &= (1-u_e)\left[b\sum_J \nu_J\left(f_{\text{0DISC}}^J\cdot g_{\text{ALLD}}^{I,J}\right)\right] \ , \\ \Pi_{\text{0DISC}}^I &= (1-u_e)\left[b\sum_J \nu_J\left(f_{\text{0DISC}}^J\cdot g_{\text{0DISC}}^{I,J}\right) - cg^{\bullet,I}\right] - \eta \ . \end{aligned}$$

0DISC invading ALLD. Non-stereotyping discriminators (0DISC) can invade a resident population of defectors (ALLD) provided $\sum_J \nu_J \left(\Pi^J_{\text{0DISC}} - \Pi^J_{\text{ALLD}}\right)|_{f_{\text{0DISC}}=0} > 0$, which simplifies to

$$-c(1-u_e)\sum_J \nu_J g^{\bullet,J}\big|_{f_{\text{0DISC}}=0} - \eta > 0\ .$$

But this inequality never holds because $g^{\bullet,J}\big|_{f_{\text{0DISC}}=0} > 0$ whenever there is a possibility of assessment errors. Hence, 0DISC cannot invade a population of ALLD.

ALLD invading 0DISC. Defectors (ALLD) can invade a resident population of non-stereotyping discriminators (0DISC) provided $\sum_J \nu_J \left(\Pi^J_{\text{ALLD}} - \Pi^J_{\text{0DISC}}\right)|_{f_{\text{ALLD}}=0} > 0$, which simplifies to

$$(1-u_e)\sum_J \nu_J \left[b\sum_{J'} \nu_{J'} \left(g^{J,J'}_{\text{ALLD}} - g^{J,J'}_{\text{0DISC}} \right) + cg^{\bullet,J} \right]\Bigg|_{f_{\text{ALLD}}=0} + \eta > 0\ .$$

2.4 Full-stereotyping discriminators (1DISC) and unconditional defectors (ALLD)

Setting $p = 1$ and $f_{\text{ALLC}} = 0$ in Eq (2), the payoffs of ALLD and 1DISC in group I are, respectively,

$$\Pi^I_{\text{ALLD}} = (1-u_e)\left[b\sum_J \nu_J \left(f^J_{\text{1DISC}} \cdot g^{I,J}_S \right) \right]\ ,$$

$$\Pi^I_{\text{1DISC}} = (1-u_e)\left[b\sum_J \nu_J \left(f^J_{\text{1DISC}} \cdot g^{I,J}_S \right) - cg^{\star,I} \right]\ ,$$

1DISC invading ALLD. Full-stereotyping discriminators (1DISC) can invade a resident population of defectors (ALLD) provided $\sum_J \nu_J \left(\Pi^J_{\text{1DISC}} - \Pi^J_{\text{ALLD}}\right)|_{f_{\text{1DISC}}=0} > 0$, which simplifies to

$$-c(1-u_e)\sum_J \nu_J g^{\star,J}\big|_{f_{\text{1DISC}}=0} > 0\ .$$

But this inequality never holds because $g^{\star,J}\big|_{f_{\text{1DISC}}=0} > 0$ whenever there is a possibility of assessment errors. Hence, 1DISC cannot invade a population of ALLD.

ALLD invading 1DISC. Defectors (ALLD) can invade a resident population of full-stereotyping discriminators (1DISC) provided $\sum_J \nu_J \left(\Pi^J_{\text{ALLD}} - \Pi^J_{\text{1DISC}}\right)|_{f_{\text{ALLD}}=0} > 0$, which simplifies to

$$c(1-u_e)\sum_J \nu_J g^{\star,J}\big|_{f_{\text{1DISC}}=0} > 0\ .$$

This inequality always holds whenever assessment errors are possible. Hence, ALLD can always invade a population of 1DISC.

3 Stability analysis

In an infinitely large population structured into two groups of equal size ($K = 2, \nu_1 = \nu_2 = 0.5$), the groups will exhibit symmetry: how group 1 sees (a subset of) itself will be identical to how group 2 sees (the corresponding subset of) itself ($g_i^{2,2} = g_i^{1,1}$ for $i \in \{\text{ALLC}, \text{ALLD}, p\text{DISC}\}$; $g_S^{2,2} = g_S^{1,1}$), and how group 1 sees (a subset of) group 2 will be identical to how group 2 sees (the corresponding subset of) group 1 ($g_i^{2,1} = g_i^{1,2}$ for $i \in \{\text{ALLC}, \text{ALLD}, p\text{DISC}\}$; $g_S^{2,1} = g_S^{1,2}$). We use these relationships to simplify the reputations equations, solve for the equilibrium reputations in a population consisting of pDISC individuals only, and compute the condition under which the all-pDISC equilibrium is locally stable. Results are reported for the Stern Judging norm and public monitoring (public stereotyped reputations and individual reputations).

3.1 Equilibrium reputations at the all-pDISC equilibrium under public monitoring

Solving Eqs (5) and (7) at the all-pDISC equilibrium ($f^I_{p\text{DISC}} = 1$) using the symmetry conditions above, we find the following equilibrium reputations under the Stern Judging norm:

$$
\begin{aligned}
g^{1,1}_{\text{ALLC}} = g^{2,2}_{\text{ALLC}} = g^{1,2}_{\text{ALLC}} = g^{2,1}_{\text{ALLC}} &= \frac{1}{(1-p)(1-u_e)(2(1-u_a)-(1-2u_a)(1-u_e))} \\
&\cdot \Big[-p(1-u_e)\big((1-(1-2u_a)(1-u_e))(1-u_a-(1-2u_a)(1-u_e)) + (1-2u_a)(1-u_e)\big) \\
&\quad - \big(-u_a^2(1-2u_e(2-u_e(3-2u_e))) + u_a((3-4u_e)u_e-2)u_e + u_a + u_e^3 + u_e - 1\big) - (1-2u_e)\sqrt{D(p,u_a,u_e)} \Big] \\
g^{1,1}_{\text{ALLD}} = g^{2,2}_{\text{ALLD}} = g^{1,2}_{\text{ALLD}} = g^{2,1}_{\text{ALLD}} & \\
&= \frac{(1-u_a)\big((1-p)(1-u_e)(1-(1-2u_a)(1-u_e)) - (1-u_a-(1-2u_a)(1-u_e))\big) + \sqrt{D(p,u_a,u_e)}}{(1-p)(1-u_e)(2(1-u_a)-(1-2u_a)(1-u_e))} \\
g^{1,1}_{p\text{DISC}} = g^{2,2}_{p\text{DISC}} = g^{1,2}_{p\text{DISC}} = g^{2,1}_{p\text{DISC}} & \\
&= \frac{(1-u_a)(1-u_a-p(1-2u_a)(1-u_e)) - \sqrt{D(p,u_a,u_e)}}{(1-p)(1-2u_a)(1-u_e)(2(1-u_a)-(1-2u_a)(1-u_e))} \\
g_S^{1,1} = g_S^{2,2} = g_S^{1,2} = g_S^{2,1} & \\
&= \frac{(1-u_a)(1-u_a-(1-p)(1-2u_a)(1-u_e)) - \sqrt{D(p,u_a,u_e)}}{p(1-2u_a)(1-u_e)(2(1-u_a)-(1-2u_a)(1-u_e))}
\end{aligned}
$$

where we define

$$D(p,u_a,u_e) = (1-u_a)(1-u_a-(1-2u_a)(1-u_e))(1-u_a-p(1-2u_a)(1-u_e))(1-u_a-(1-p)(1-2u_a)(1-u_e))$$

for concision.

3.2 Cooperation levels at the all-pDISC equilibrium under public monitoring

Let $F(p,u_a,u_e)$ be the cooperation level in a pDISC population under public monitoring, computed numerically in Fig 2A. From the equilibrium reputations above, we can derive an explicit expression for F:

$$
\begin{aligned}
F(p,u_a,u_e) &= \frac{1}{2}(1-u_e)\left[(1-p)\left(g^{1,1}_{p\text{DISC}} + g^{1,2}_{p\text{DISC}}\right) + p\left(g_S^{1,1} + g_S^{1,2}\right)\right] \\
&= \frac{1+u_e-u_a(1+(3-2u_a)u_e) - 2\sqrt{D(p,u_a,u_e)}}{1+u_e-2u_a-4(1-u_a)u_a u_e} .
\end{aligned}
$$

Interestingly, we find that $F(p) = F(1-p)$; in other words, the cooperation level is symmetric about the line $p = 0.5$, consistent with Fig 2A. To gain some intuition for this fact, we consider the edge case with no execution errors ($u_e = 0$) and compute the Taylor expansion of the cooperation level F around $u_a = 0$:

$$
\begin{aligned}
F(p,u_a,0) &= \frac{1-u_a-2\sqrt{(1-u_a)(1+u_a)(1-u_a-p(1-2u_a))(1-u_a-(1-p)(1-2u_a))}}{1-2u_a} \\
&= 1 - 2\sqrt{p(1-p)}\sqrt{u_a} + u_a + O\left(u_a^{3/2}\right) .
\end{aligned}
$$

From the Taylor expansion, we see that, when $u_e = 0$ and u_a is small, F depends on p via the term $\sqrt{p(1-p)}$ (up to first order in u_a), consistent with the symmetry discussed above.

3.3 Stability of the all-pDISC equilibrium under public monitoring

Next we use the equilibrium reputations to derive conditions for local stability of the all-pDISC equilibrium by evaluating the Jacobian of the replicator dynamics (Eq 1) at that equilibrium.

The all-pDISC equilibrium with $p = 0$ is locally stable if the access cost (η) is not too high. When $p = 0$, that is, when pDISC individuals always use individual reputations, the pDISC-only equilibrium is locally stable under Stern Judging if and only if one of the following sets of conditions is satisfied:

$$\text{(I)} \quad \frac{1}{(1-2u_a)(1-u_e)} < \frac{b}{c} \le \frac{2(1-u_a)-(1-2u_a)(1-u_e)}{(1-2u_a)^2(1-u_e)^2} \quad \text{and} \quad \frac{\eta}{c} < \frac{(1-u_e)(1-u_a)\left(\frac{b}{c}(1-2u_a)(1-u_e)-1\right)}{2(1-u_a)-(1-2u_a)(1-u_e)}$$

$$\text{(II)} \quad \frac{b}{c} > \frac{2(1-u_a)-(1-2u_a)(1-u_e)}{(1-2u_a)^2(1-u_e)^2} \quad \text{and} \quad \frac{\eta}{c} < \frac{(1-u_e)(1-u_a-(1-2u_a)(1-u_e))\left(\frac{b}{c}(1-2u_a)(1-u_e)+1\right)}{2(1-u_a)-(1-2u_a)(1-u_e)}$$

In either case, there is an upper bound to the value of η/c that makes pDISC stable; that is, 0DISC will be unstable if the access cost for reputations (η) is too large relative to the cost of cooperation (c). Note that each upper bound segment is linear in b/c (as visualized in Fig A).

The all-pDISC equilibrium with $0 < p < 1$ can also be locally stable if the access cost (η) is not too high. We numerically compute the threshold value of η below which the all-pDISC equilibrium with $p = 0.25, 0.5$, or 0.75 is locally stable (Fig A). When b/c is small, cooperation is *harder* to stabilize with a larger p: for example, while 0DISC can be stable for sufficiently small η when $b/c < 7$, 0.75DISC cannot. In contrast, when b/c is large, cooperation is *easier* to stabilize with a larger p: for example, the maximum value of η for which 0.75DISC can be stable is higher than the corresponding value for 0DISC.

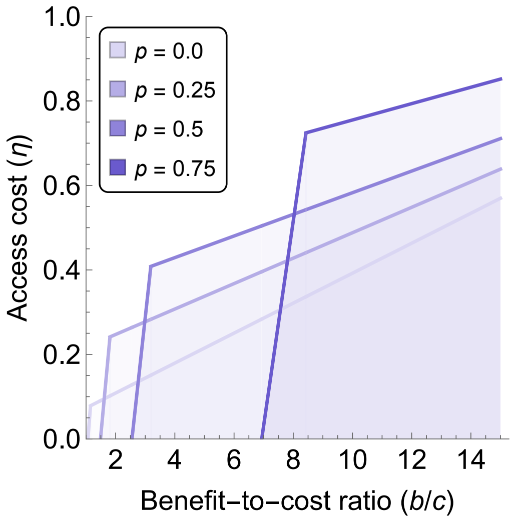

Figure A: Local stability of the pDISC equilibrium. In general, the pDISC-only equilibrium is locally stable when b/c is high and η is low, with the specific region for each p highlighted in color ($p = 0$ in light purple to $p = 0.75$ in dark purple). The boundaries for $p = 0$ are given by conditions (I) and (II) above.

In the special case of $\eta = 0$, we can derive an analytical condition for 0.5DISC to be locally stable:

$$\frac{b}{c} > \frac{2}{(1-2u_a)(1-u_e)}\left(1+\sqrt{1-\frac{(1-2u_a)(1-u_e)}{1-u_a}}\right).$$

The all-pDISC equilibrium with $p = 1$ is never locally stable. By contrast, when $p = 1$, that is, when pDISC individuals always use stereotyped reputations, the pDISC-only equilibrium can never be stable within the parameter constraints (e.g., $0 < u_a, u_e < 1/2$).

4 Special case: symmetric groups with $u_e = 0$

In this section, we obtain simplified expressions for the equations governing the reputation dynamics, under the assumption of two equally sized groups ($\nu_1 = \nu_2 = 1/2$) and strategic frequencies equal across groups (all $f_J^i := f_i$). Then, we derive analytical solutions for the reputation equations *and* the conditions under which pDISC resists invasion by ALLD.

For two symmetric groups, we are assured that

$$\begin{aligned} g_i^{1,1} &= g_i^{2,2} := g_i^{\text{in}}, \\ g_i^{1,2} &= g_i^{2,1} := g_i^{\text{out}}; \end{aligned}$$

we may likewise define

$$\begin{aligned} g^{\text{in}} &= \sum_i f_i g_i^{\text{in}}, \\ g^{\text{out}} &= \sum_i f_i g_i^{\text{out}}. \end{aligned}$$

First, we have

$$\begin{aligned} g^{\bullet,I} &= \sum_J \nu_J \sum_i f_i^J g_i^{J,I} \\ &= \frac{1}{2} \sum_i f_i \sum_J g_i^{J,I} \\ &= \frac{1}{2} \sum_i f_i \left(g_i^{\text{in}} + g_i^{\text{out}} \right) \\ &= \frac{1}{2} \left(g^{\text{in}} + g^{\text{out}} \right) \\ &:= g. \end{aligned}$$

By similar reasoning, we have

$$\begin{aligned} g_S^{1,1} &= g_S^{2,2} := g_S^{\text{in}}, \\ g_S^{1,2} &= g_S^{2,1} := g_S^{\text{out}}, \end{aligned}$$

and

$$\begin{aligned} g^{\star,I} &= \sum_J \nu_J g_S^{J,I} \\ &= \frac{1}{2} \left(g_S^{\text{in}} + g_S^{\text{out}} \right) \\ &:= g_S. \end{aligned}$$

Our disagreement terms likewise become easier to interpret. When $J = I$, we obtain

$$\begin{aligned} g_{\alpha,1}^{\text{in}} &= \frac{1}{2} \sum_i f_i \left[(g_i^{\text{in}})^2 + (g_i^{\text{out}})^2 \right], \\ g_{\alpha,2}^{\text{in}} &= \sum_L \frac{1}{2} \sum_i f_i g_S^{L,J} g_i^{L,I} \\ &= \frac{1}{2} \sum_i f_i \left(g_S^{\text{in}} g_i^{\text{in}} + g_S^{\text{out}} g_i^{\text{out}} \right), \\ g_{\alpha,3}^{\text{in}} &= \sum_L \frac{1}{2} \sum_i f_i g_S^{L,I} g_i^{L,J} \\ &= \frac{1}{2} \sum_i f_i \left(g_S^{\text{in}} g_i^{\text{in}} + g_S^{\text{out}} g_i^{\text{out}} \right) = g_{\alpha,2}^{\text{in}}, \\ g_{\alpha,4}^{\text{in}} &= \frac{1}{2} \left[(g_S^{\text{in}})^2 + (g_S^{\text{out}})^2 \right] \end{aligned}$$

When $J \neq I$, we obtain

$$\begin{aligned}
g_{\alpha,1}^{\text{out}} &= \sum_L \frac{1}{2} \sum_i f_i g_i^{\text{in}} g_i^{\text{out}} \\
&= \sum_i f_i g_i^{\text{in}} g_i^{\text{out}}, \\
g_{\alpha,2}^{\text{out}} &= \sum_L \frac{1}{2} \sum_i f_i g_S^{L,J} g_i^{L,I} \\
&= \frac{1}{2} \sum_i f_i \left(g_S^{\text{in}} g_i^{\text{out}} + g_S^{\text{out}} g_i^{\text{in}} \right), \\
g_{\alpha,3}^{\text{out}} &= \sum_L \frac{1}{2} \sum_i f_i g_S^{L,I} g_i^{L,J} \\
&= \frac{1}{2} \sum_i f_i \left(g_S^{\text{in}} g_i^{\text{out}} + g_S^{\text{out}} g_i^{\text{in}} \right) = g_{\alpha,2}^{\text{out}}, \\
g_{\alpha,4}^{\text{out}} &= \sum_L \frac{1}{2} g_S^{\text{in}} g_S^{\text{out}} \\
&= g_S^{\text{in}} g_S^{\text{out}}.
\end{aligned}$$

We can thus write down

$$\begin{aligned}
g_{\text{ALLC}}^{\text{in}} = g_{\text{ALLC}}^{\text{out}} &= g P_{GC} + (1-g) P_{BC}, \\
g_{\text{ALLD}}^{\text{in}} = g_{\text{ALLD}}^{\text{out}} &= g P_{GD} + (1-g) P_{BD}, \\
g_{\text{public}}^{\text{in}} = g_{\text{public}}^{\text{out}} &= g P_{GC} + (1-g) P_{BD}, \\
g_{\text{private}}^{\text{in}} &= g_{\alpha,1}^{\text{in}} (P_{GC} - P_{GD} - P_{BC} + P_{BD}) + g(P_{GD} + P_{BC} - 2P_{BD}) + P_{BD}, \\
g_{\text{private}}^{\text{out}} &= g_{\alpha,1}^{\text{out}} (P_{GC} - P_{GD} - P_{BC} + P_{BD}) + g(P_{GD} + P_{BC} - 2P_{BD}) + P_{BD}, \\
g_{\text{independent}}^{\text{in}} &= g_{\alpha,2}^{\text{in}} (P_{GC} - P_{GD} - P_{BC} + P_{BD}) + g(P_{GD} - P_{BD}) + g_S(P_{BC} - P_{BD}) + P_{BD}, \\
g_{\text{independent}}^{\text{out}} &= g_{\alpha,2}^{\text{out}} (P_{GC} - P_{GD} - P_{BC} + P_{BD}) + g(P_{GD} - P_{BD}) + g_S(P_{BC} - P_{BD}) + P_{BD}, \\
\therefore\ g_{p\text{DISC}}^{\text{in}} &= \begin{cases} (1-p) g_{\text{private}}^{\text{in}} + p g_{\text{independent}}^{\text{in}}, & \text{private individual reputations,} \\ (1-p) g_{\text{public}}^{\text{in}} + p g_{\text{independent}}^{\text{in}}, & \text{group-wise or public individual reputations,} \end{cases} \\
g_{p\text{DISC}}^{\text{out}} &= \begin{cases} (1-p) g_{\text{private}}^{\text{out}} + p g_{\text{independent}}^{\text{out}}, & \text{private or group-wise individual reputations,} \\ (1-p) g_{\text{public}}^{\text{out}} + p g_{\text{independent}}^{\text{out}}, & \text{public individual reputations.} \end{cases}
\end{aligned}$$

Similarly,

$$\begin{aligned}
g_{S,\text{ALLC}}^{\text{in}} = g_{S,\text{ALLC}}^{\text{out}} &= g_S P_{GC} + (1-g_S) P_{BC}, \\
g_{S,\text{ALLD}}^{\text{in}} = g_{S,\text{ALLD}}^{\text{out}} &= g_S P_{GD} + (1-g_S) P_{BD}, \\
g_{S,\text{public}}^{\text{in}} = g_{S,\text{public}}^{\text{out}} &= g_S P_{GC} + (1-g_S) P_{BD}, \\
g_{S,\text{private}}^{\text{in}} &= g_{\alpha,4}^{\text{in}} (P_{GC} - P_{GD} - P_{BC} + P_{BD}) + g_S(P_{GD} + P_{BC} - 2P_{BD}) + P_{BD}, \\
g_{S,\text{private}}^{\text{out}} &= g_{\alpha,4}^{\text{out}} (P_{GC} - P_{GD} - P_{BC} + P_{BD}) + g_S(P_{GD} + P_{BC} - 2P_{BD}) + P_{BD}, \\
g_{S,\text{independent}}^{\text{in}} &= g_{\alpha,3}^{\text{in}} (P_{GC} - P_{GD} - P_{BC} + P_{BD}) + g_S(P_{GD} - P_{BD}) + g(P_{BC} - P_{BD}) + P_{BD}, \\
g_{S,\text{independent}}^{\text{out}} &= g_{\alpha,3}^{\text{out}} (P_{GC} - P_{GD} - P_{BC} + P_{BD}) + g_S(P_{GD} - P_{BD}) + g(P_{BC} - P_{BD}) + P_{BD},
\end{aligned}$$

and so we may write

$$\begin{aligned}
g_{S,p\text{DISC}}^{\text{in}} &= \begin{cases} (1-p) g_{S,\text{independent}}^{\text{in}} + p g_{S,\text{private}}^{\text{in}}, & \text{private stereotyped reputations,} \\ (1-p) g_{S,\text{independent}}^{\text{in}} + p g_{S,\text{public}}^{\text{in}}, & \text{group-wise or public stereotyped reputations,} \end{cases} \\
g_{S,p\text{DISC}}^{\text{out}} &= \begin{cases} (1-p) g_{S,\text{independent}}^{\text{out}} + p g_{S,\text{private}}^{\text{out}}, & \text{private or group-wise stereotyped reputations,} \\ (1-p) g_{S,\text{independent}}^{\text{out}} + p g_{S,\text{public}}^{\text{out}}, & \text{public stereotyped reputations,} \end{cases}
\end{aligned}$$

with

$$g_S^{\text{in}} = f_{\text{ALLC}} g_{S,\text{ALLC}} + f_{\text{ALLD}} g_{S,\text{ALLD}} + f_{p\text{DISC}} g_{S,p\text{DISC}}^{\text{in}},$$
$$g_S^{\text{out}} = f_{\text{ALLC}} g_{S,\text{ALLC}} + f_{\text{ALLD}} g_{S,\text{ALLD}} + f_{p\text{DISC}} g_{S,p\text{DISC}}^{\text{out}}.$$

Reputation solutions for $f_{p\text{DISC}} = 1$ and $u_e = 0$

Fixing the entire population for discriminators (pDISC) and sending the error rate u_e to zero yields intuitive, easily parseable solutions. It is worth recalling that, with *no* stereotypes, we have, under Stern Judging and group-wise individual reputations,

$$g_{\text{DISC}}^{\text{in}} = 1 - u_a,$$
$$g_{\text{DISC}}^{\text{out}} = \frac{1}{2};$$

A population of DISC resists invasion by ALLD provided

$$\frac{b}{c} > \left(\frac{b}{c}\right)^* = \frac{g_{\text{DISC}}}{g_{\text{DISC}} - g_{\text{ALLD}}} = \frac{3 - 2u_a}{2(1-u_a)(1-2u_a)}.$$

For public reputations, the entire population uses the $g_{\text{DISC}}^{\text{in}}$ expression, in which case $(b/c)^* = 1/(1-2u_a)$, and for private reputations, the entire population uses the $g_{\text{DISC}}^{\text{out}}$ expression, in which case $(b/c)^*$ blows up.

We proceed to derive analytical solutions to the reputation equations when $f_{p\text{DISC}} = 1$. We also interrogate the circumstances under which a population of pDISC, under different stereotyping scenarios, can resist invasion by ALLD. We will see that full stereotyping *never* allows pDISC to resist invasion by ALLD under *any* scenario for evaluating individual and stereotyped reputations. In general, stability requires

$$\begin{aligned}
0 &< \sum_J \nu_J \Pi_{p\text{DISC}}^J - \sum_J \nu_J \Pi_{\text{ALLD}}^J \\
0 &< \left(b\left[(1-p)g_{p\text{DISC}} + pg_S\right] - c\left[(1-p)g + pg_S\right] - \eta(1-p)\right) \\
&\quad - b\left[(1-p)g_{\text{ALLD}} + pg_S\right] \\
\therefore \frac{1}{c}\left(b - \frac{\eta}{g_{p\text{DISC}} - g_{\text{ALLD}}}\right) &> \frac{g_{p\text{DISC}} + \frac{p}{1-p}g_S}{g_{p\text{DISC}} - g_{\text{ALLD}}}, \\
\text{with } g_{p\text{DISC}} &\coloneqq \frac{1}{2}(g_{p\text{DISC}}^{\text{in}} + g_{p\text{DISC}}^{\text{out}}).
\end{aligned}$$

In the following expressions, we set $\eta = 0$ for simplicity, so the ratio on the left hand side is simply b/c, as in the no-stereotyping case.

1. Under private individual reputations and stereotypes, we have

   $$g_{\text{ALLC}} = g_{\text{ALLD}} = g_{p\text{DISC}}^{\text{in}} = g_{p\text{DISC}}^{\text{out}} = g_S^{\text{in}} = g_S^{\text{out}} = \frac{1}{2}.$$

   Such a population can never resist invasion by ALLD.

2. With private individual reputations and group-wise stereotypes, the solutions are the same, except

   $$g_S^{\text{in}} = \frac{1}{2} + p\left(\frac{1}{2} - u_a\right).$$

   For $p = 0$, this is identical to the private stereotype case; as p increases, the ingroup stereotyped reputation moves toward $1 - u_a$. As in the private stereotype case, the population can never resist invasion by ALLD.

3. For private individual reputations and public stereotypes, we have

$$g_S^{\text{out}} = g_S^{\text{in}} = \frac{1}{2} + p\left(\frac{1}{2} - u_a\right);$$

both the in- and out-group stereotyped reputations interpolate between the fully private and fully public values as p changes. The population can never resist invasion by ALLD.

4. Under group-wise individual reputations and private stereotypes, we have

$$\begin{aligned}
g_{\text{ALLC}} &= \frac{1}{2} + (1-p)\left[\frac{1}{4} - u_a(1-u_a)\right], \\
g_{\text{ALLD}} &= \frac{1}{4} + u_a(1-u_a) + p\left[\frac{1}{4} - u_a(1-u_a)\right], \\
g_{p\text{DISC}}^{\text{in}} &= 1 - u_a - p\left(\frac{1}{2} + u_a\right), \\
g_{p\text{DISC}}^{\text{out}} &= g_S^{\text{in}} = g_S^{\text{in}} = \frac{1}{2}.
\end{aligned}$$

Again as p increases, all reputations tend toward $1/2$. The critical b/c ratio is

$$\left(\frac{b}{c}\right)^* = \frac{3 - 2u_a + p(1-2p)(1-2u_a)}{2(1-p)^2(1-u_a)(1-2u_a)}.$$

Predictably, this goes to infinity as p approaches 1 and is more stringent than the condition for stability under group-wise reputations and no stereotyping (Eq 4).

5. With group-wise individual reputations and group-wise stereotypes,

$$\begin{aligned}
g_{\text{ALLC}} &= \frac{7 - 6p - 4(1-2p)u_a(1-u_a) - \xi}{8(1-p)}, \\
g_{\text{ALLD}} &= \frac{(1-2p)[1 + 4u_a(1-u_a)] + \xi}{8(1-p)}, \\
g_{p\text{DISC}}^{\text{in}} &= \frac{5 - 8u_a + 4u_a^2 - 4p(1-2u_a)(1-u_a) - \xi}{4(1-p)(1-2u_a)}, \\
g_{p\text{DISC}}^{\text{out}} &= \frac{1}{2}, \\
g_S^{\text{in}} &= \frac{1 + 4u_a(1-u_a) + 4p(1-2u_a)(1-u_a) - \xi}{4p(1-2u_a)}, \\
g_S^{\text{out}} &= \frac{1}{2}, \\
\text{with } \xi &= \sqrt{[1 + 4u_a(1-u_a)]\,[1 + 4u_a(1-u_a) + 4p(1-p)(1-2u_a)^2]},
\end{aligned}$$

The critical b/c ratio is

$$\left(\frac{b}{c}\right)^* = \frac{4(1-u_a) - \xi}{(1-u_a)(3 - 2p(1-2u_a)^2 - 4u_a(1-u_a) - \xi},$$

which blows up as p approaches 1 and simplifies to Eq 4 for $p = 0$.

6. With group-wise individual reputations and public stereotypes, the full expressions are difficult to parse, but in the

limit of $u_a \to 0$,

$$\begin{aligned} g_{\text{ALLC}} &= \frac{14-11p-\omega}{16(1-p)}, \\ g_{\text{ALLD}} &= \frac{2-5p+\omega}{16(1-p)}, \\ g^{\text{in}}_{p\text{DISC}} &= \frac{6-3p+\omega}{4(1-p)}, \\ g^{\text{out}}_{p\text{DISC}} &= \frac{2-5p+\omega}{8(1-p)}, \\ g^{\text{in}}_{S} &= \frac{2+5p-\omega}{4p}, \\ g^{\text{out}}_{S} &= \frac{2+5p-\omega}{4p}, \\ \text{with } \omega &= \sqrt{(2-p)(2+7p)}. \end{aligned}$$

The b/c condition is

$$\left(\frac{b}{c}\right)^* = \frac{22+9p-5\omega}{12-6p-2\omega}.$$

Sending p to 0 yields $3/2$, consistent with Eq 4.

7. With public individual reputations and private stereotypes,

$$\begin{aligned} g_{\text{ALLC}} &= 1-2u_a(1-u_a)-\frac{1}{2}p(1-2u_a)^2, \\ g_{\text{ALLD}} &= 2u_a(1-u_a)+\frac{1}{2}p(1-2u_a)^2, \\ g^{\text{in}}_{p\text{DISC}} = g^{\text{out}}_{p\text{DISC}} &= 1-u_a-p\left(\frac{1}{2}+u_a\right), \\ g^{\text{in}}_{S} = g^{\text{out}}_{S} &= \frac{1}{2}. \end{aligned}$$

Note that, when $p=0$, we have $g_{\text{ALLD}} = 1-g_{\text{ALLC}}$, as in the case of no stereotyping. As p increases, the reputations for all strategic types tend toward $1/2$. The critical b/c condition is

$$\left(\frac{b}{c}\right)^* = \frac{2(1-u_a)+p(1-p)(1-2u_a)}{2(1-p)^2(1-u_a)(1-2u_a)},$$

which becomes $1/(1-2u_a)$ (public version of Eq 4) for $p=0$ and blows up as p approaches 1.

8. With public individual reputations and group-wise stereotypes, the full expressions are (as in the case of group-wise individual reputations and public stereotypes) difficult to parse, but in the limit of $u_a \to 0$,

$$\begin{aligned} g_{\text{ALLC}} &= \frac{7-5p-\chi}{4(1-p)}, \\ g_{\text{ALLD}} &= \frac{3-p-\chi}{4(1-p)}, \\ g^{\text{in}}_{p\text{DISC}} &= \frac{7-5p-\chi}{4(1-p)}, \\ g^{\text{out}}_{p\text{DISC}} &= \frac{7-5p-\chi}{4(1-p)}, \\ g^{\text{in}}_{S} &= \frac{3+3p-\chi}{4p}, \\ g^{\text{out}}_{S} &= \frac{-3+5p+\chi}{4p}, \\ \text{with } \chi &= \sqrt{(9-7p)(1+p)}. \end{aligned}$$

The critical b/c ratio is

$$\left(\frac{b}{c}\right)^* = \frac{31 - 9p - 5\chi}{8(5 - 3p - \chi)},$$

which goes to 1 at $p = 0$, consistent with the public version of Eq 4.

9. Finally, with public individual reputations and public stereotypes,

$$\begin{aligned} g_{\text{ALLC}} &= \frac{1 - u_a(1-u_a) - p[1 - 2u_a(1-u_a)] - \psi}{1-p}, \\ g_{\text{ALLD}} &= \frac{(1-2p)u_a(1-u_a) + \psi}{1-p}, \\ g_{p\text{DISC}}^{\text{in}} = g_{p\text{DISC}}^{\text{out}} &= \frac{(1-u_a)^2 - p(1-2u_a)(1-u_a) - \psi}{(1-p)(1-2u_a)}, \\ g_S^{\text{in}} = g_S^{\text{out}} &= \frac{u_a(1-u_a) + p(1-2u_a)(1-u_a) - \psi}{p(1-2u_a)}, \\ \text{with } \psi &= \sqrt{u_a(1-u_a)\left[p(1-p)(1-2u_a)^2 + u_a(1-u_a)\right]}. \end{aligned}$$

As expected, these are consistent with the expressions in Section 3.2, under the transformation $\psi = \sqrt{D(p, u_a, u_e)}$ and sending $u_e \to 0$. The critical b/c ratio is

$$\left(\frac{b}{c}\right)^* = \frac{1 - u_a - 2\psi}{(1-u_a)[1 - p(1-2u_a)^2 - 2u_a(1-u_a) - 2\psi]}.$$

This, too, simplifies to the expected $1/(1-2u_a)$ when $p = 0$.

The all-pDISC equilibrium with $p = 1$ is never locally stable under *any* method for evaluating individual and stereotyped reputations. Just as we showed in the case of public monitoring, full stereotyping always allows ALLD to invade a population of pDISC, irrespective of how stereotyped reputations are evaluated.
