# Supplementary material for: When do stereotypes undermine indirect reciprocity?
Source: PLoS Comput Biol. 2024 Mar 1;20(3):e1011862. doi: 10.1371/journal.pcbi.1011862 (PMC10906830; doi:10.1371/journal.pcbi.1011862)
Supplement: S1 Table — (PDF) [file pcbi.1011862.s002.pdf]

Quantity	Definition	Baseline values
K	number of groups	2
ν_I	fraction of the population in group I	0.5
b	benefit of cooperation	3
c	cost of cooperation	1
η	cost of accessing individual reputations	0.3
p	stereotype-use propensity, i.e., probability that a pDISC individual uses stereotyped reputations rather than individual reputations	0 (no stereotyping); 1 (full stereotyping)
u_a	probability that a bad donor is accidentally assigned a good reputation (assessment error rate)	0.02
u_e	probability that a donor intending to cooperate accidentally defects (execution error rate)	0.02
ε	probability that an individual who intends to cooperate with a recipient with a good reputation is assigned a good reputation ($\varepsilon = (1-u_e)(1-u_a)+u_e u_a$)	0.9608 for baseline u_a and u_e
q_C	probability that cooperating with a bad individual yields a good reputation (barring errors)	see Materials and Methods
q_D	probability that defecting against a bad individual yields a good reputation (barring errors)	see Materials and Methods
P_{XY}	probability that a donor intending to $Y \in \{\text{cooperate, defect}\}$ with a recipient whom the observer views as $X \in \{\text{good, bad}\}$ is viewed as good	see Materials and Methods
g_{ALLC}	probability that an ALLC has a good reputation	—
g_{ALLD}	probability that an ALLD has a good reputation	—
$g_{p\text{DISC}}$	probability that a pDISC has a good reputation	—
f_i^I	frequency of strategy i in group I	—
Π_i^I	fitness of a strategy i individual in group I	—
$\bar{\Pi}^I$	average fitness of individuals in group I	—
$g_i^{I,J}$	fraction of strategy i individuals in group I who have good individual reputations in the eyes of J	—
$g^{I,J}$	fraction of I individuals with good individual reputations in the eyes of J	—
$g_S^{I,J}$	fraction of I individuals with good stereotyped reputations in the eyes of J	—
