# Supplementary figures and images for: When do stereotypes undermine indirect reciprocity?
Source: PLoS Comput Biol. 2024 Mar 1;20(3):e1011862. doi: 10.1371/journal.pcbi.1011862 (PMC10906830; doi:10.1371/journal.pcbi.1011862)

Cooperation level

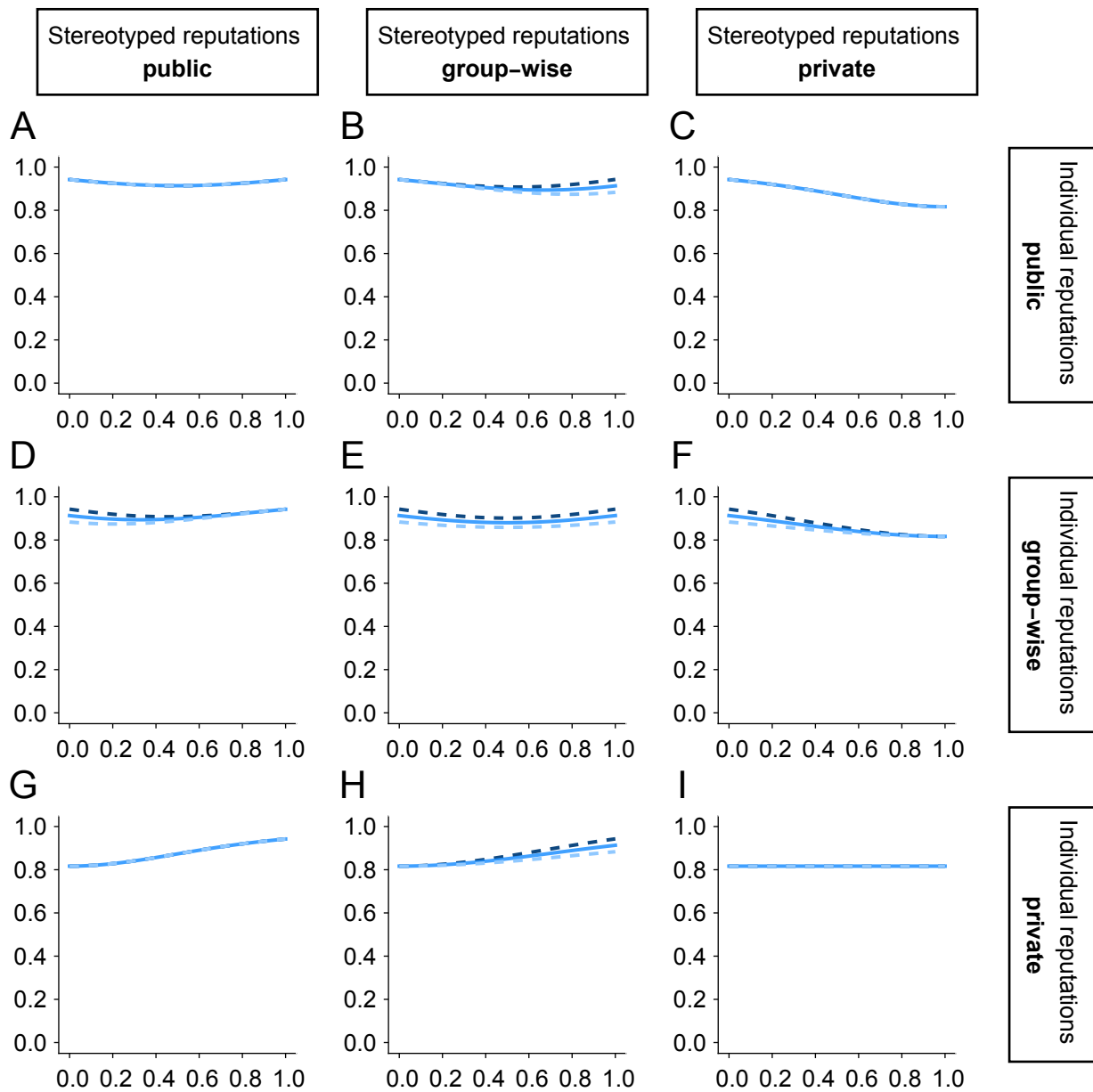

Stereotype-use propensity ( $p$ )

Supplement: S1 Fig — As in Fig 2, but under the Simple Standing norm. (PDF) [file pcbi.1011862.s003.pdf]

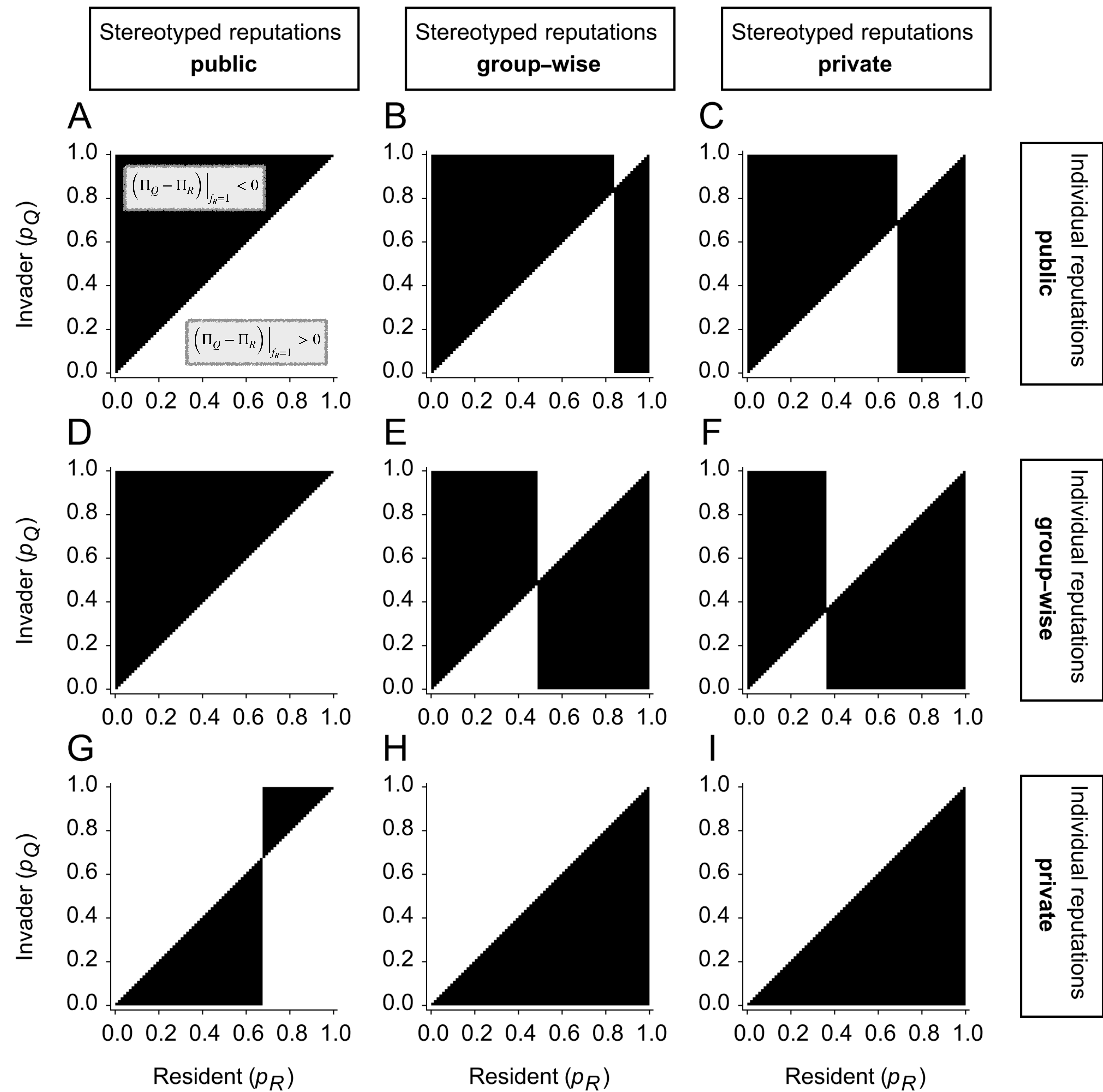

Supplement: S4 Fig — We use adaptive dynamics to predict the dynamics of stereotype-use propensity p under the Stern Judging norm. Pairwise invasibility plots indicate parameter regions in which pQ can invade pR (white), i.e., invader payoff ΠQ exceeds resident payoff ΠR in the limit of negligible invader frequency, or not (black) (Pairwise invasibility analysis in Materials and methods). Each panel shows a combination of monitoring systems for individual reputations (rows) and stereotyped reputations (columns). Orange arrows indicate predicted dynamics of p over time. Payoff parameters are b = 3, c = 1, and η = 0.3; error rates are ua = ue = 0.02. (PDF) [file pcbi.1011862.s006.pdf]

Stereotype-use propensity ( $p$ )

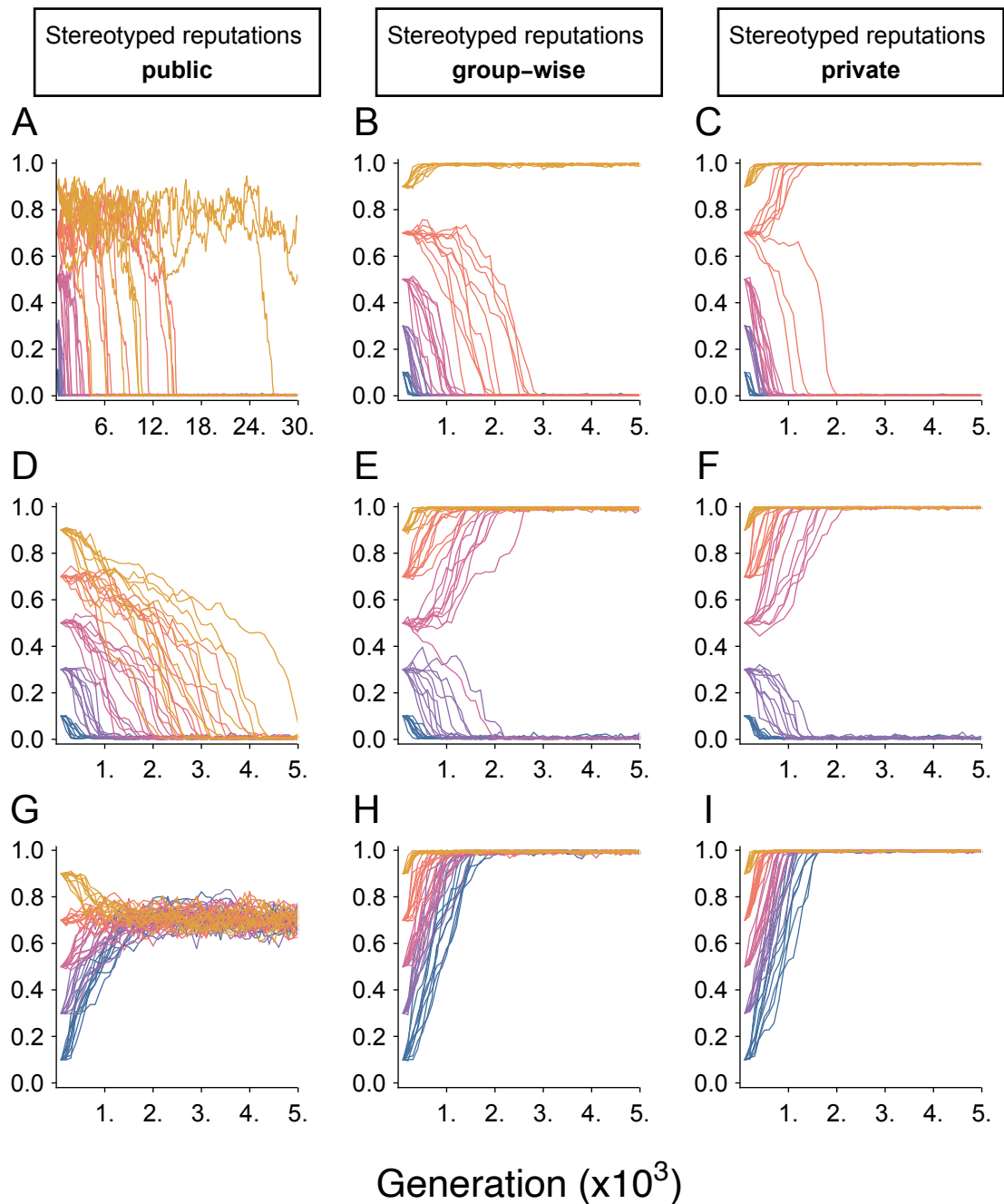

Supplement: S5 Fig — Stochastic simulations under the Stern Judging norm in finite populations of N = 50 with small, local mutations (Stochastic simulations in Materials and methods) support the predictions based on adaptive dynamics (S4 Fig), with rare exceptions (C: three simulation runs starting from p = 0.7 go to p = 0; E: one simulation run starting from p = 0.5 goes to p = 0) likely due to mutations moving the population above or below the singular value. Lines indicate mean stereotype-use propensity p in the population over time. Colors distinguish initial conditions (monomorphic populations with uniform p), with 10 simulation runs per initial condition. Data are sampled every 100 time steps. Each panel shows a combination of monitoring systems for individual reputations (rows) and stereotyped reputations (columns). (PDF) [file pcbi.1011862.s007.pdf]

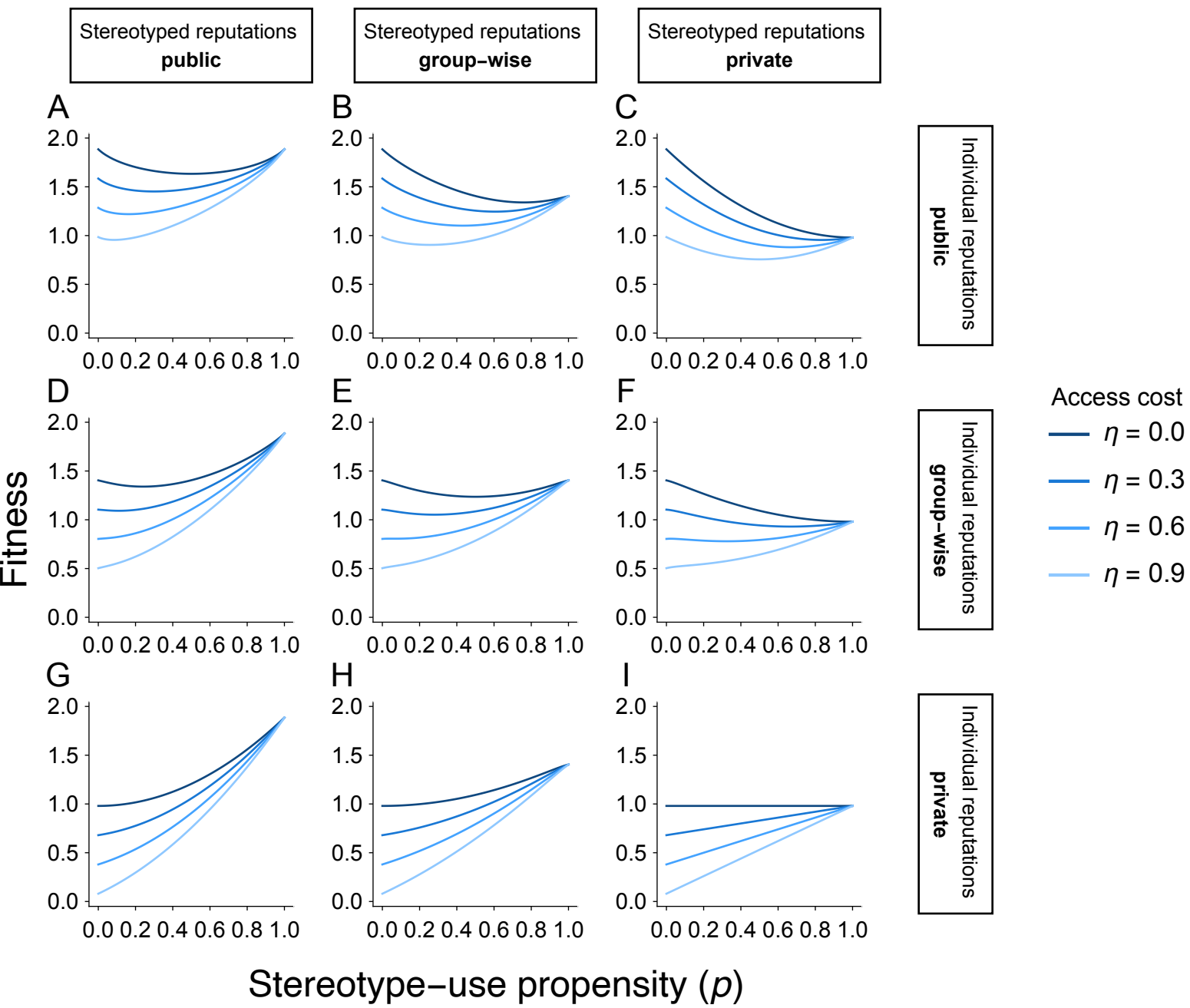

Supplement: S6 Fig — We analyzed individual fitness levels under the Stern Judging norm among pDISC strategists with a uniform stereotype-use propensity p. As in Fig 2, individuals are in two groups of equal size (K = 2, ν1 = ν2 = 0.5). Each panel shows a combination of monitoring systems for individual (row) and stereotyped (column) reputations. Color indicates access cost η. Parameters: b = 3, c = 1, ua = ue = 0.02. (PDF) [file pcbi.1011862.s008.pdf]

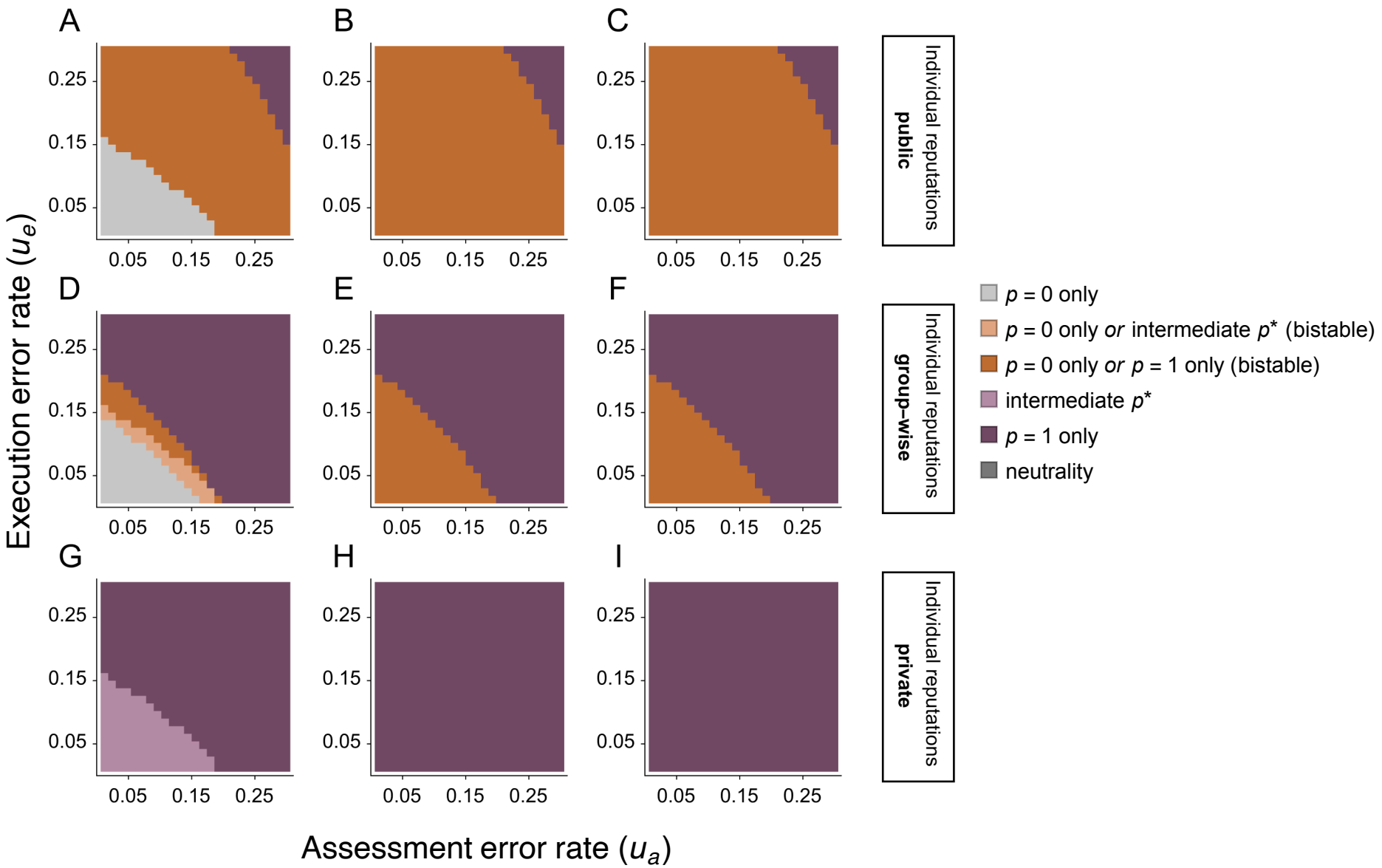

Supplement: S8 Fig — As in S7 Fig, but with varying rates of assessment (ua) and execution (ue) errors. Errors in assessment are more harmful to individual reputations than for stereotyped reputations, because each assessment introduces the possibility of an erroneous judgment. A single observation is used to assign a stereotype to a group of N/K individuals, whereas N/K observations are required to assign individual reputations to each member of the group. This means that stereotyping confers a roughly N/K-fold decrease in the expected number of evaluation errors. Errors in strategy execution also have more negative consequences under individual reputations than under stereotypes. A donor who defects erroneously is more likely to get a bad individual reputation, at least under Stern Judging, which makes others less likely to cooperate with her. However, if the donor is part of a group with a good stereotype, she may still be seen as good. And so relying on stereotypes can help mitigate the vicious cycle of bad reputations and reduced cooperation that is initiated by erroneous actions or judgments. Results are shown for the Stern Judging norm, as in S7 Fig. Parameters: b = 3, c = 1, η = 0.3. (PDF) [file pcbi.1011862.s010.pdf]

Stereotype-use propensity ( $p$ )

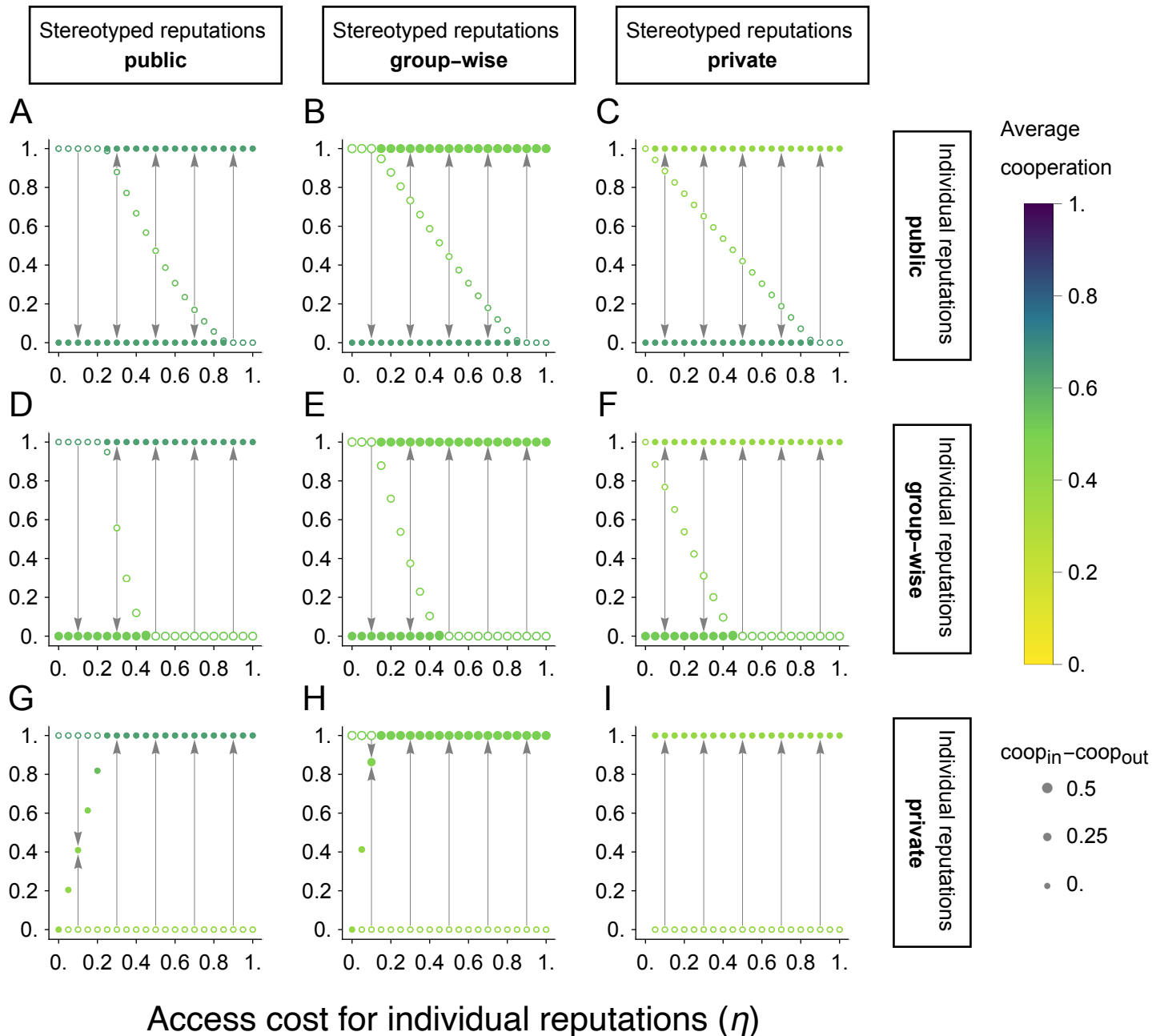

Supplement: S9 Fig — We repeat the analysis by adaptive dynamics as in Fig 3 but in a population in which 20% of individuals are unconditional defectors (ALLD) who do not change strategies. Each panel shows a combination of monitoring systems for individual (row) and stereotyped (column) reputations. Solid (empty) circles denote attractive (repulsive) singular points for p. Gray arrows denote the attractive points toward which the population converges for given values of η and initial values of p. Colors indicate the average level of cooperation for each singular point (Fig 2). Circle size indicates the difference between in- and out-group cooperation levels; larger sizes indicate larger differences. Qualitatively, the outcomes for p are similar to the case without ALLD in the population (Fig 3); in particular, in a majority of the parameter conditions studied here, the population will adopt either full stereotyping (p = 1) or no stereotyping (p = 0) in the long term. Quantitatively, the levels of cooperation achieved at the singular points are lower than in the absence of ALLD (Fig 3). Results are shown for the Stern Judging norm, as in Fig 3. Parameters: b = 3, c = 1, ue = ua = 0.02. (PDF) [file pcbi.1011862.s011.pdf]

ALLD, ALLC,  $p$ DISC

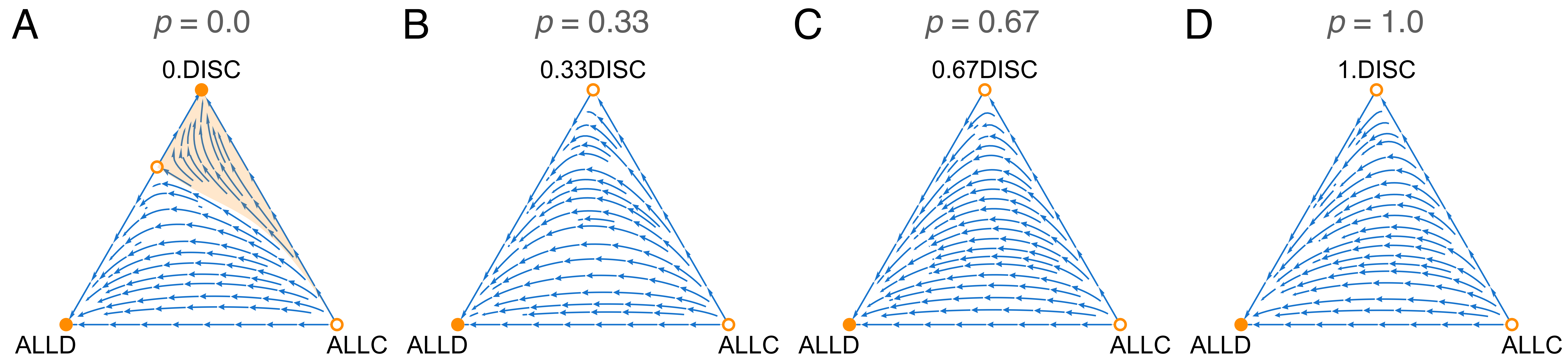

ALLD, 0DISC, 1DISC

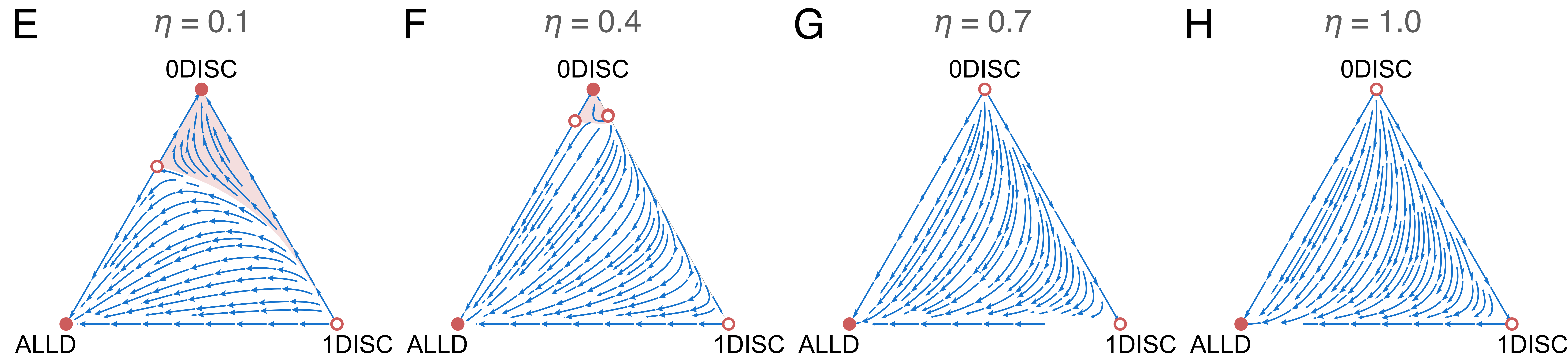

Supplement: S10 Fig — As in Fig 4, but with group-wise monitoring for both individual and stereotyped reputations. The outcomes for both sets of strategies (A–D: ALLD, ALLC and in pDISC; E–H: ALLD, 0DISC, and 1DISC) are qualitatively similar to the corresponding results under public monitoring (Fig 4). Results are shown for the Stern Judging norm, as in Fig 4. (PDF) [file pcbi.1011862.s012.pdf]

**A**  $\eta = 0.1$

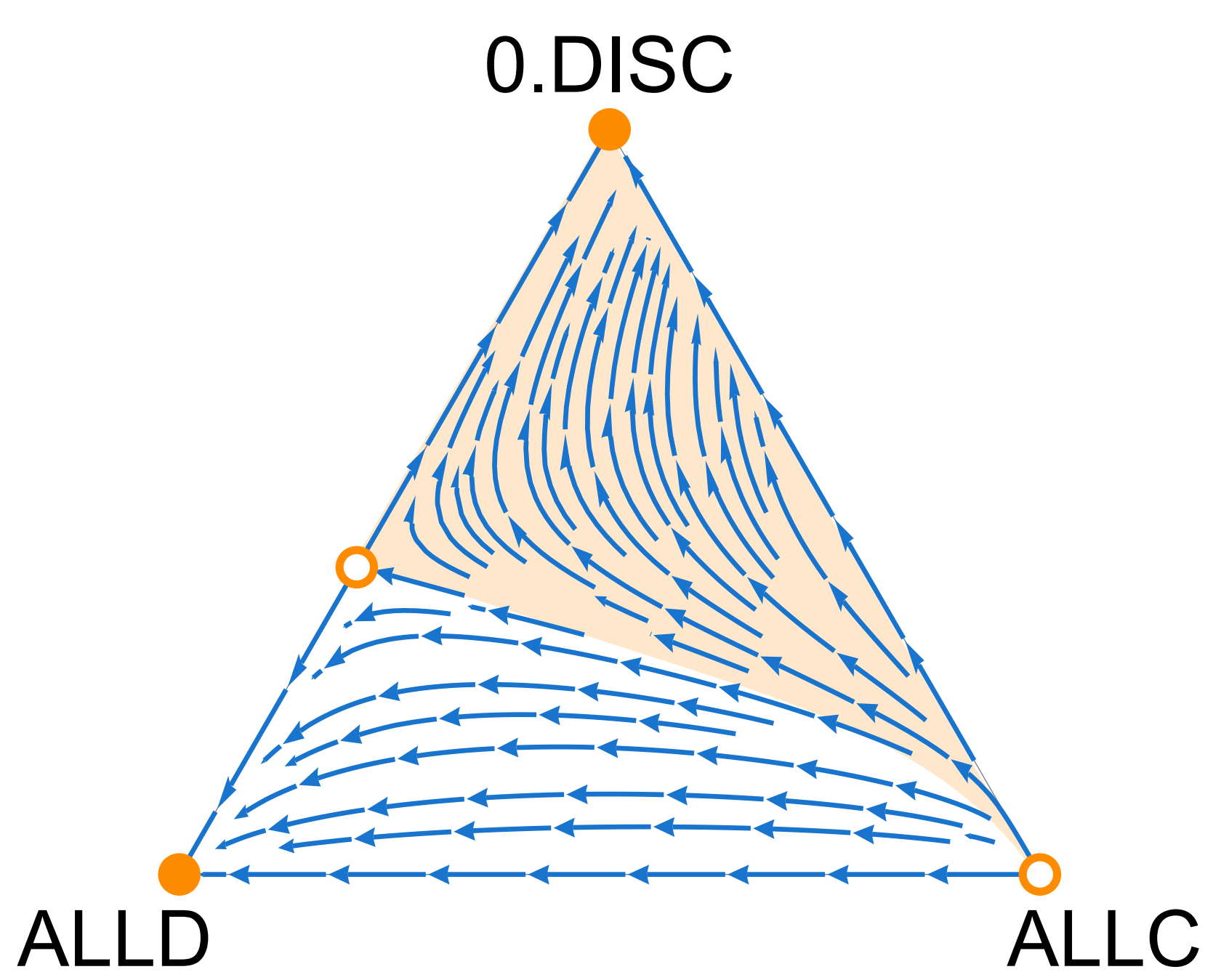

**B**  $\eta = 0.2$

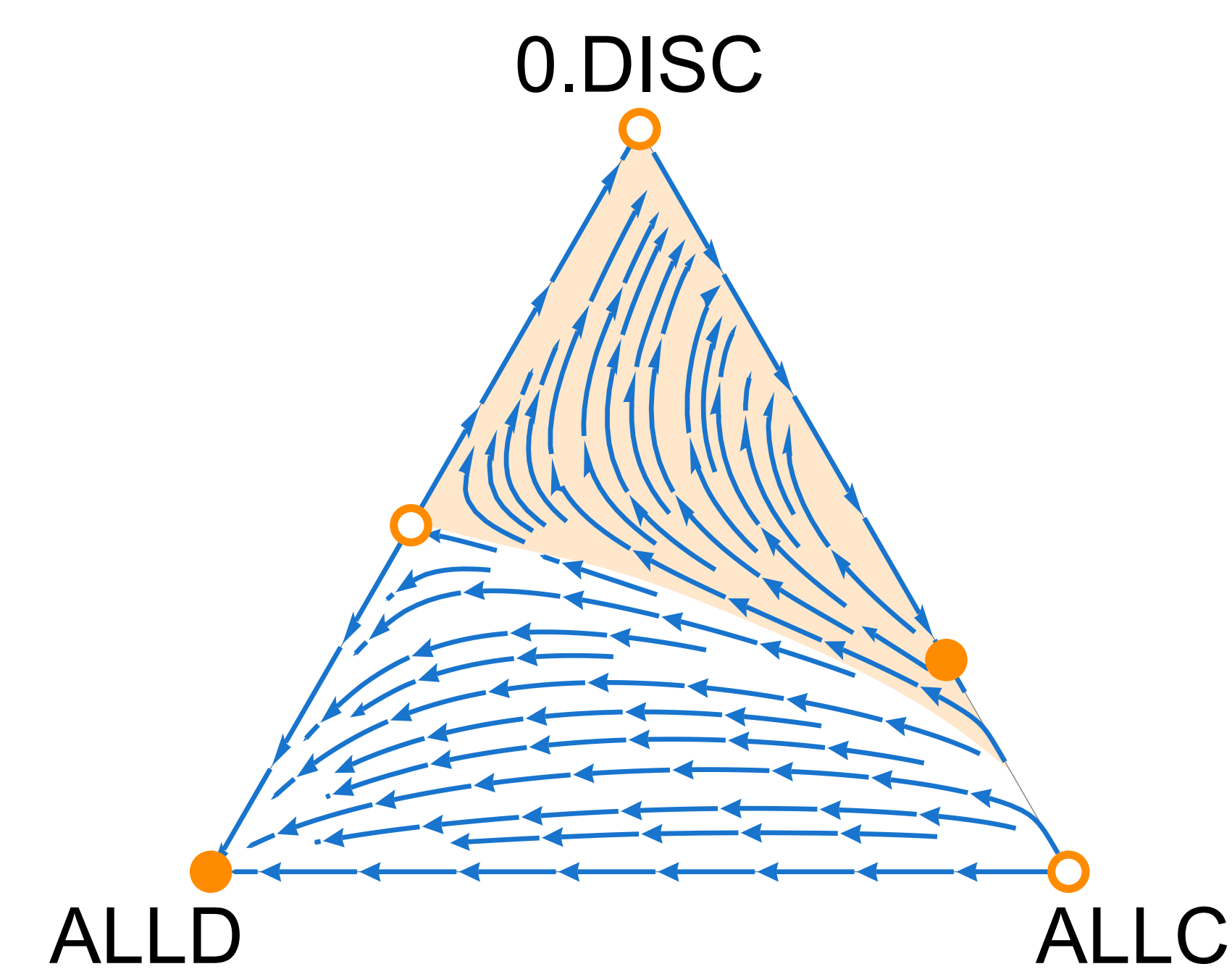

**C**  $\eta = 0.3$

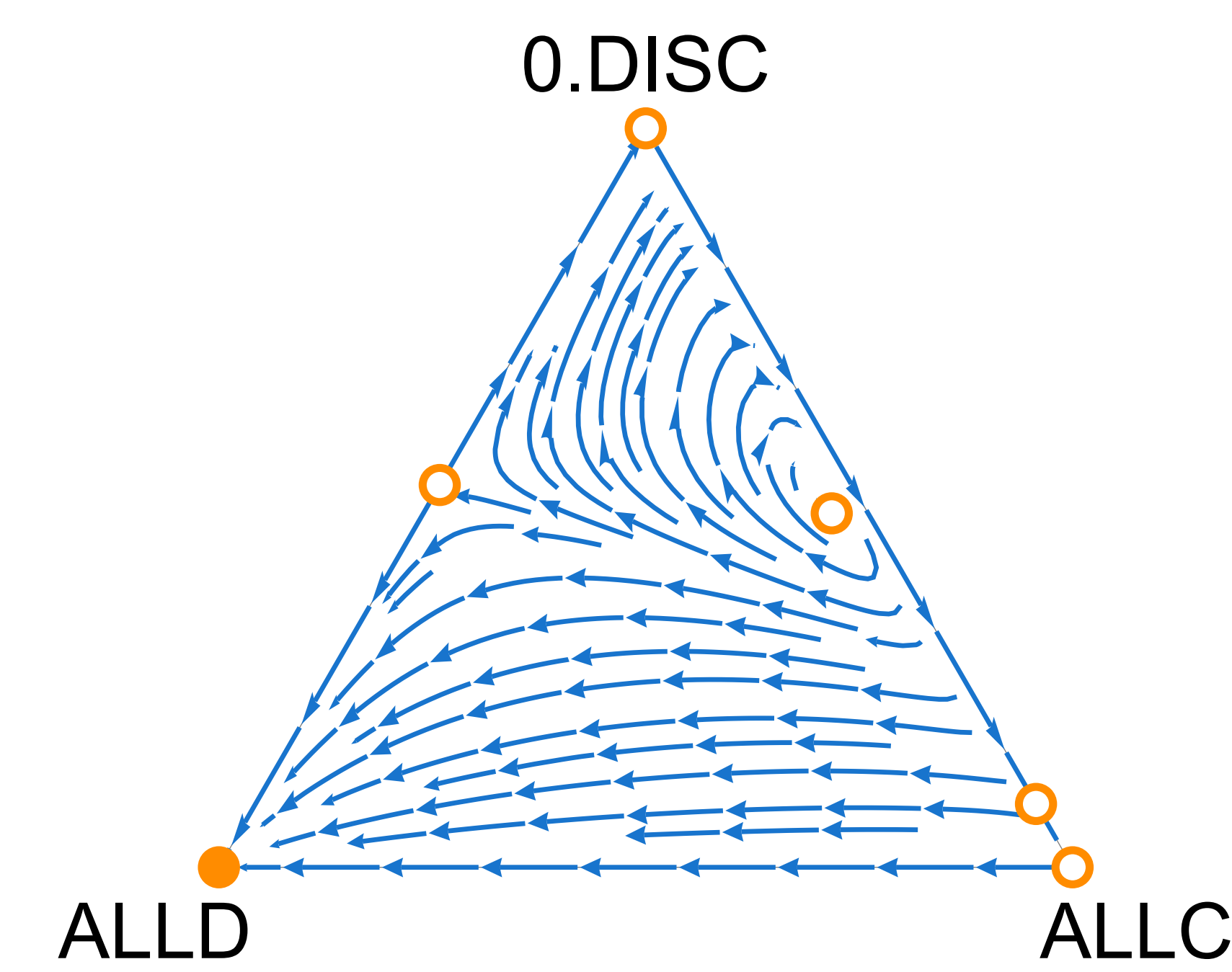

**D**  $\eta = 0.4$

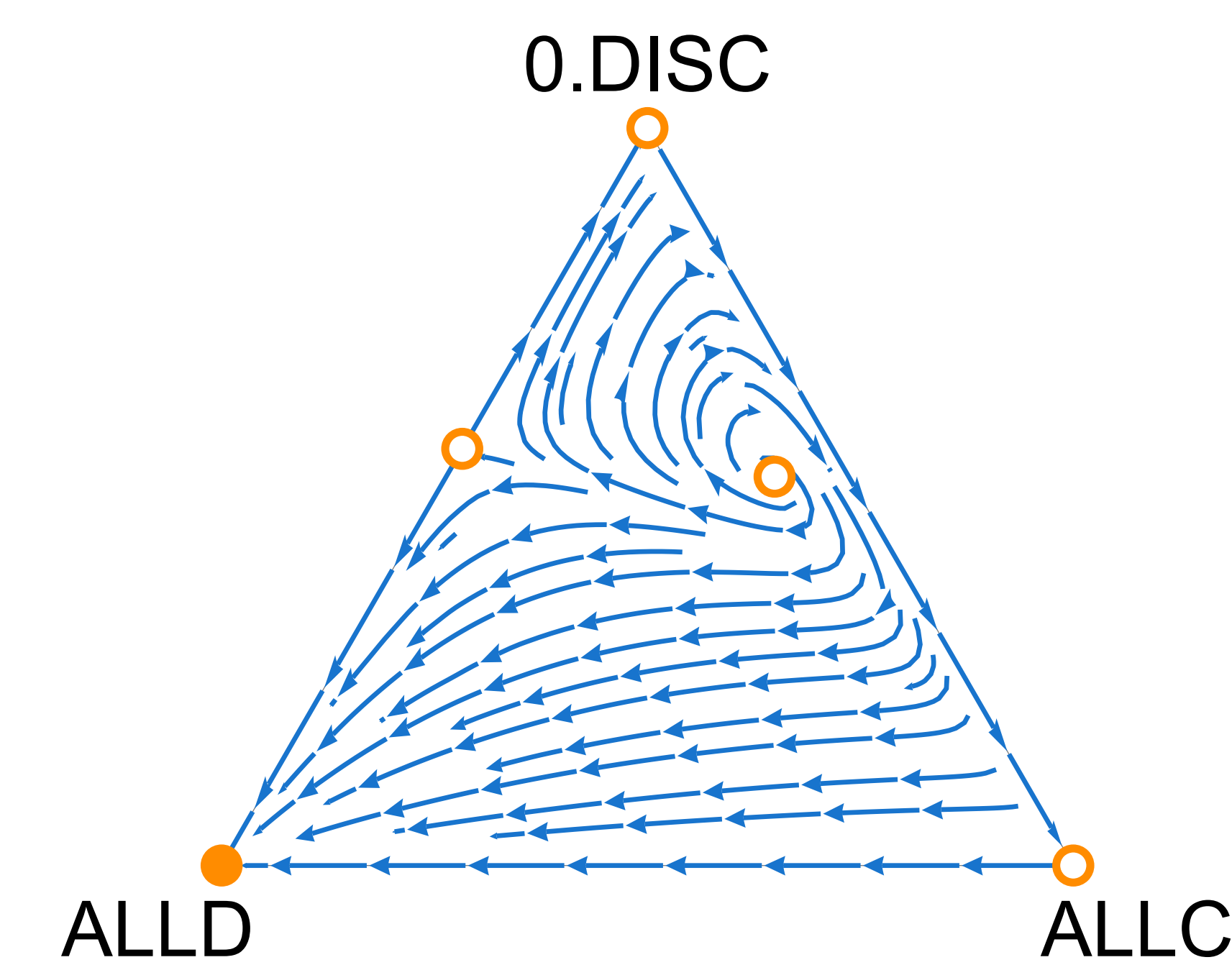

**E**  $\eta = 0.7$

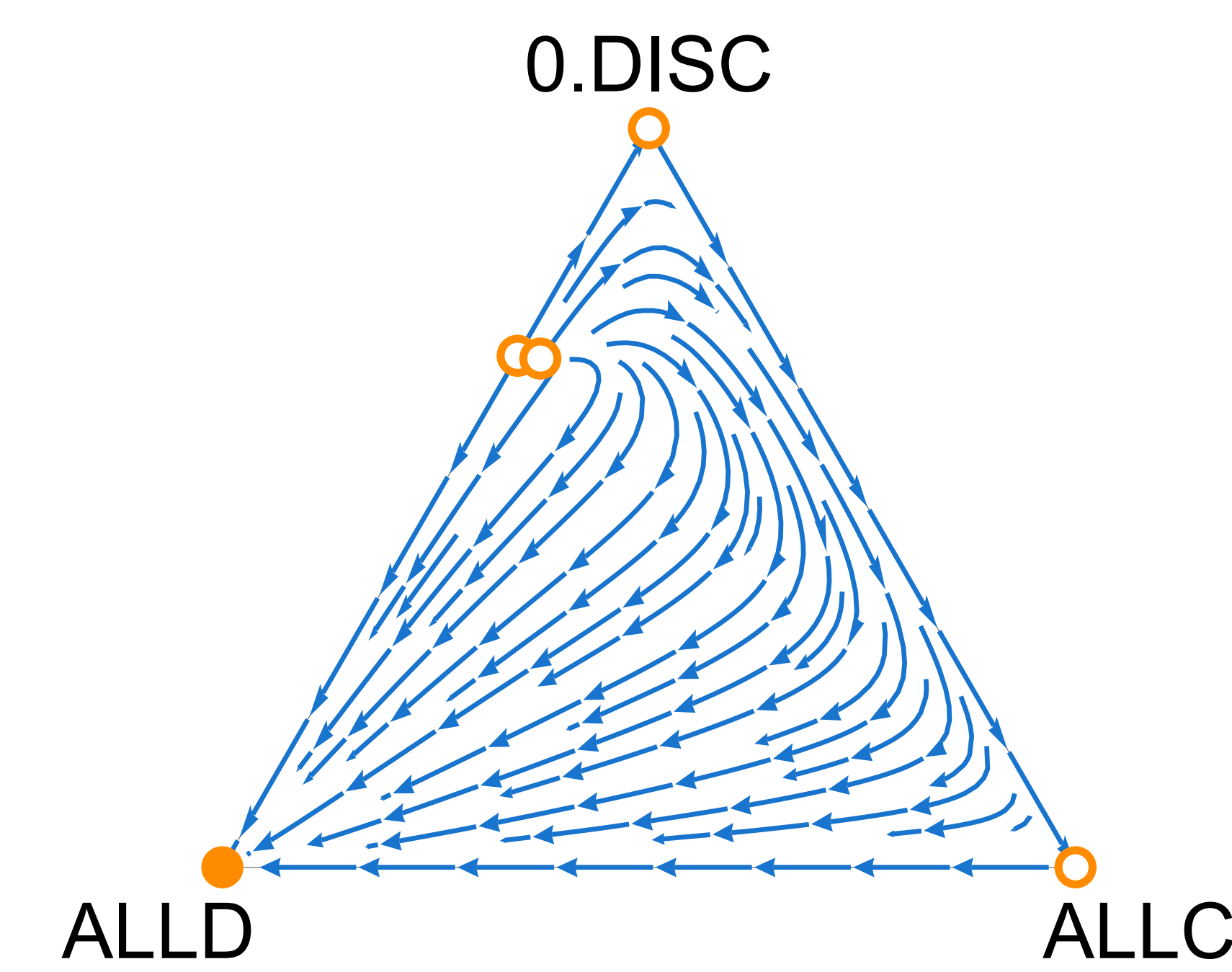

**F**  $\eta = 1.0$

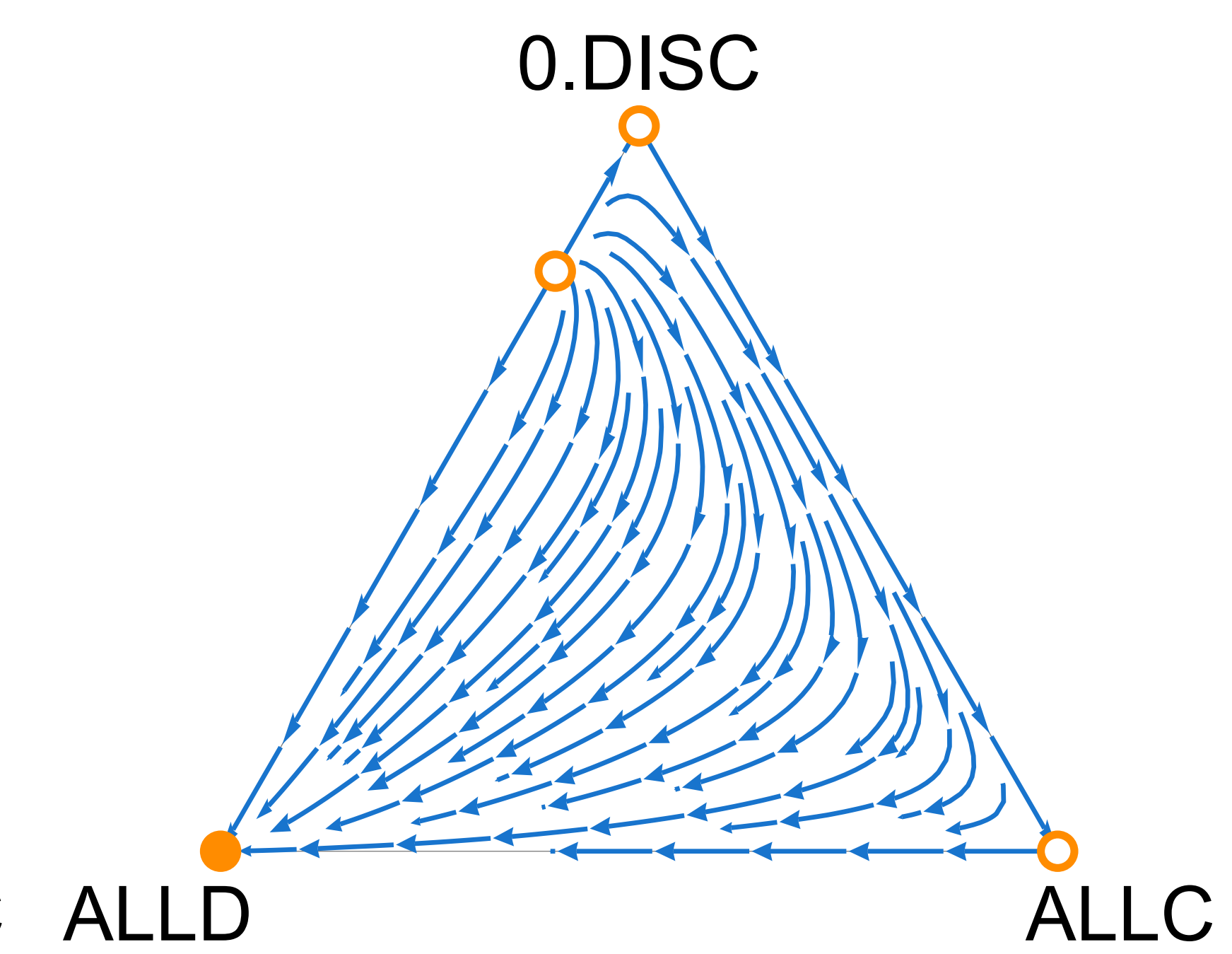

Supplement: S11 Fig — Just as stereotyping discriminators and tag-based cooperators can destabilize cooperation (Fig 4 and S12 Fig), unconditional cooperators can also make a population vulnerable to defection when individual reputations are costly. However, the mechanism by which the latter creates a pathway toward defection is qualitatively different. To demonstrate this, we analyze competition among cooperators (ALLC), defectors (ALLD), and non-stereotyping discriminators (0DISC), but with varying access cost η. Arrows indicate the dynamical flow within the simplex of three competing strategies. All reputations are public information assessed according to the Stern Judging norm. Individuals are distributed across two groups of equal size (K = 2, ν1 = ν2 = 0.5). When individual reputations are inexpensive (low η), there is a large basin of attraction toward either the 0DISC vertex (A) or a stable mixed equilibrium with ALLC and 0DISC (B), each of which can sustain high levels of cooperation. However, this cooperative basin disappears as soon as η is high enough such that the ALLC-0DISC equilibrium becomes unstable (C), after which defection is the only stable outcome (C–F). Hence, unlike in the presence of stereotyping, where the cooperative basin shrinks gradually (Fig 4), increasing access cost leads to a discontinuous loss of cooperation in the absence of stereotyping. Parameters: b = 3, c = 1, ue = ua = 0.02. In addition to η = 0.1, 0.4, 0.7, 1.0 used in Fig 4 and S10 Fig, we also show η = 0.2 and η = 0.3 to highlight the details of the transition from cases in which cooperation is sustained (A, B) to those in which cooperation is not sustained (C–F). (PDF) [file pcbi.1011862.s013.pdf]

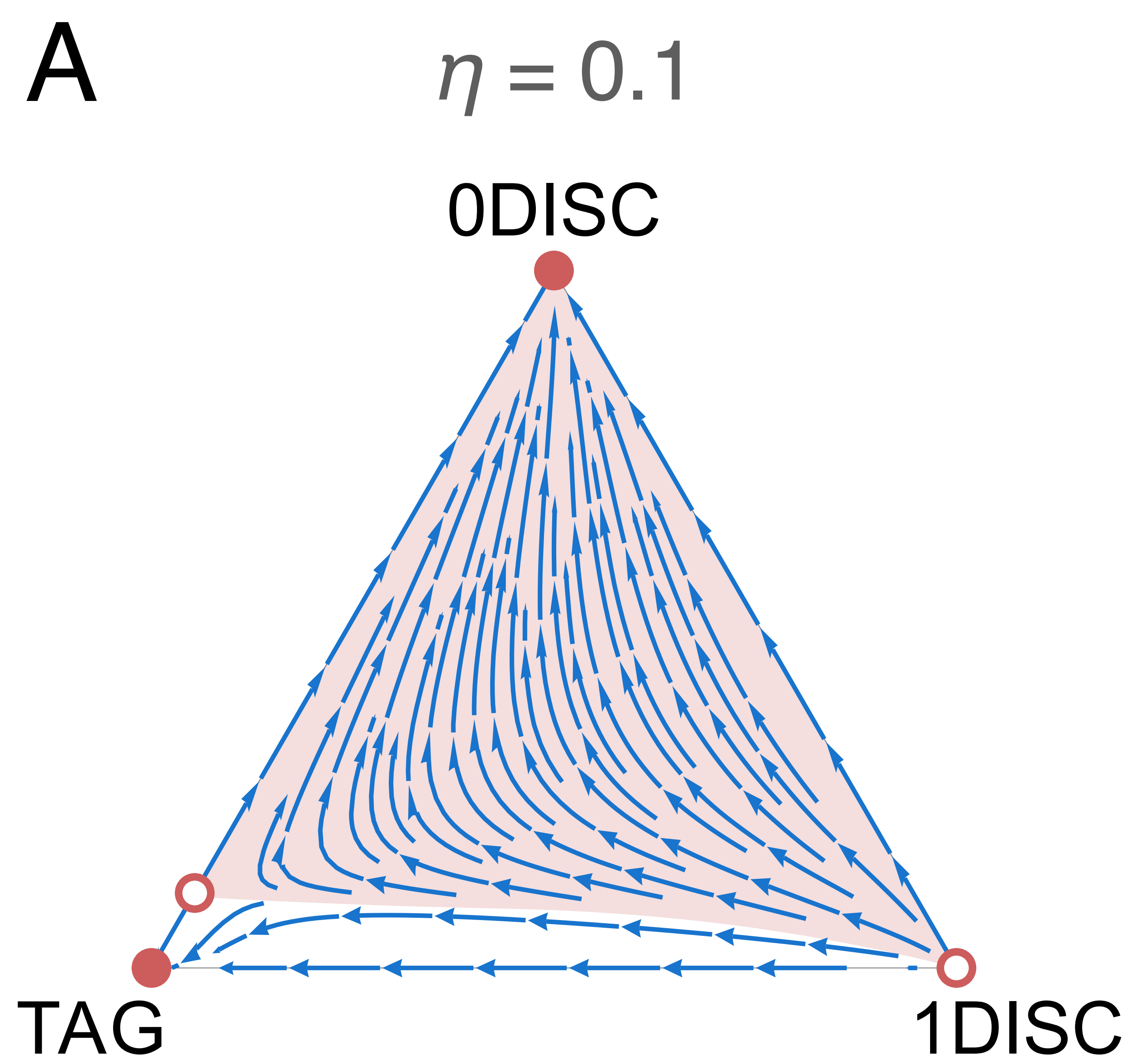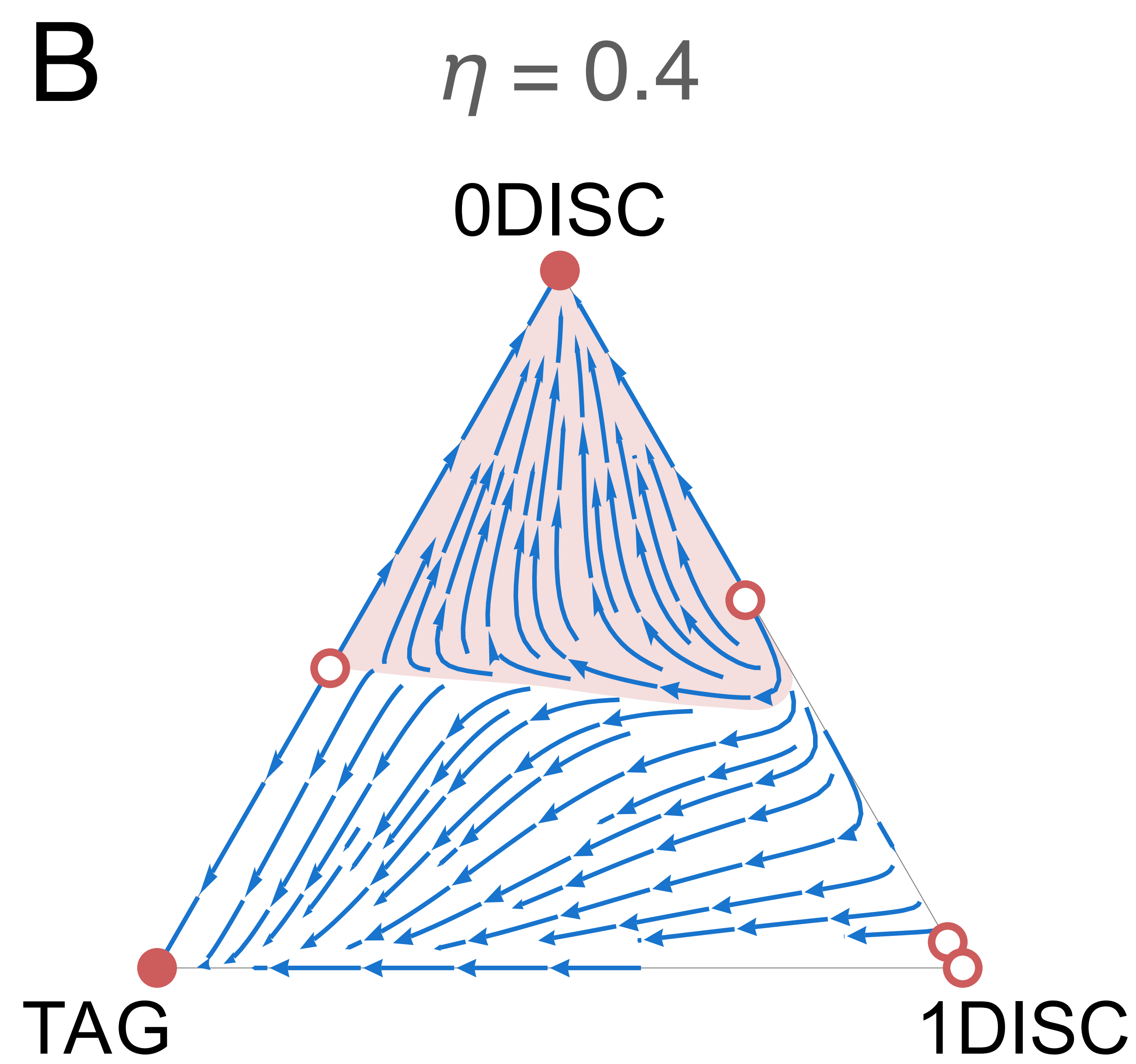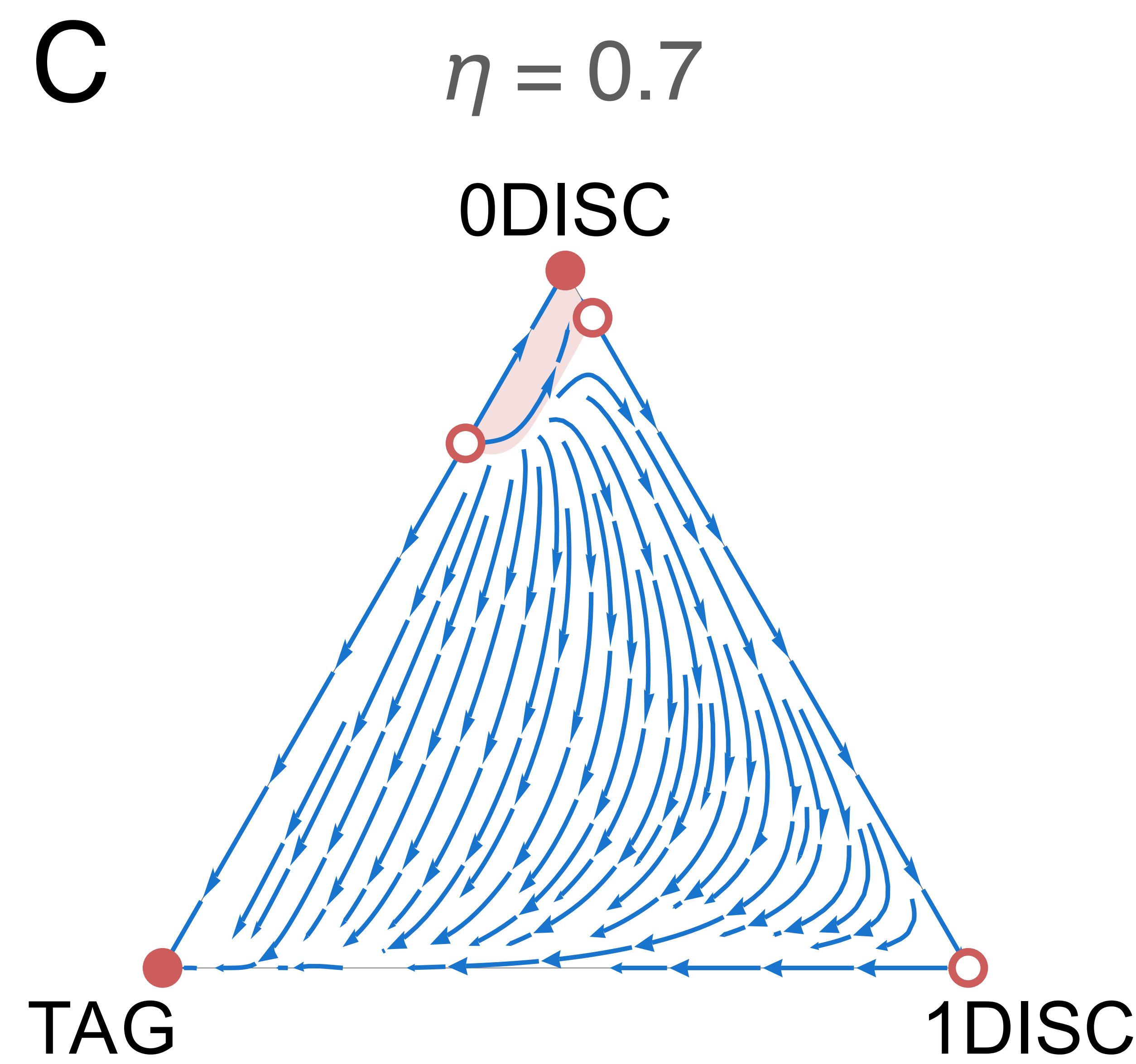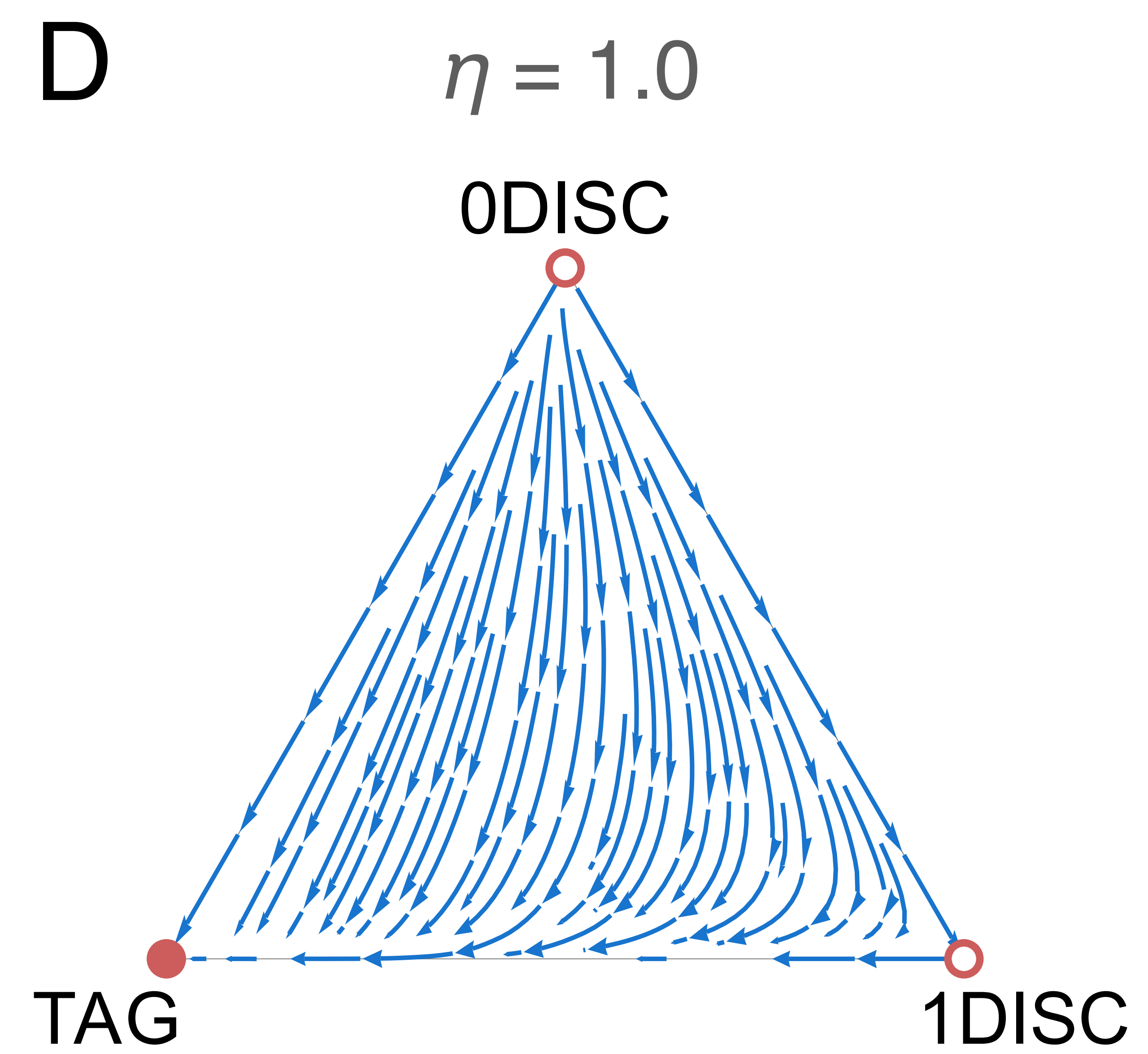

Supplement: S12 Fig — Arrows indicate the dynamical flow within the simplex of three competing strategies. All reputations are public information assessed according to the Stern Judging norm. Individuals are distributed across two groups of equal size (K = 2, ν1 = ν2 = 0.5). TAG exhibits behavior qualitatively similar to ALLD when competing with 0DISC and 1DISC (A–D versus Fig 4E–4H). The basin of attraction towards the 0DISC vertex, which produces high levels of cooperation (Fig 2), is largest when individual reputations are inexpensive (low η; A), but it quickly shrinks with increasing η (B–D). For sufficiently high access cost (D), 0DISC can be invaded not only by TAG but also by 1DISC, which, in turn, can be invaded by TAG—so that pure tribalism (TAG) is the only stable outcome. Parameters: b = 3, c = 1, ue = ua = 0.02. (PDF) [file pcbi.1011862.s014.pdf]
