# Supplementary material for: When do stereotypes undermine indirect reciprocity?
Source: PLoS Comput Biol. 2024 Mar 1;20(3):e1011862. doi: 10.1371/journal.pcbi.1011862 (PMC10906830; doi:10.1371/journal.pcbi.1011862)
Supplement: S2 Fig — As in Fig 2, but under the Shunning norm. (PDF) [file pcbi.1011862.s004.pdf]

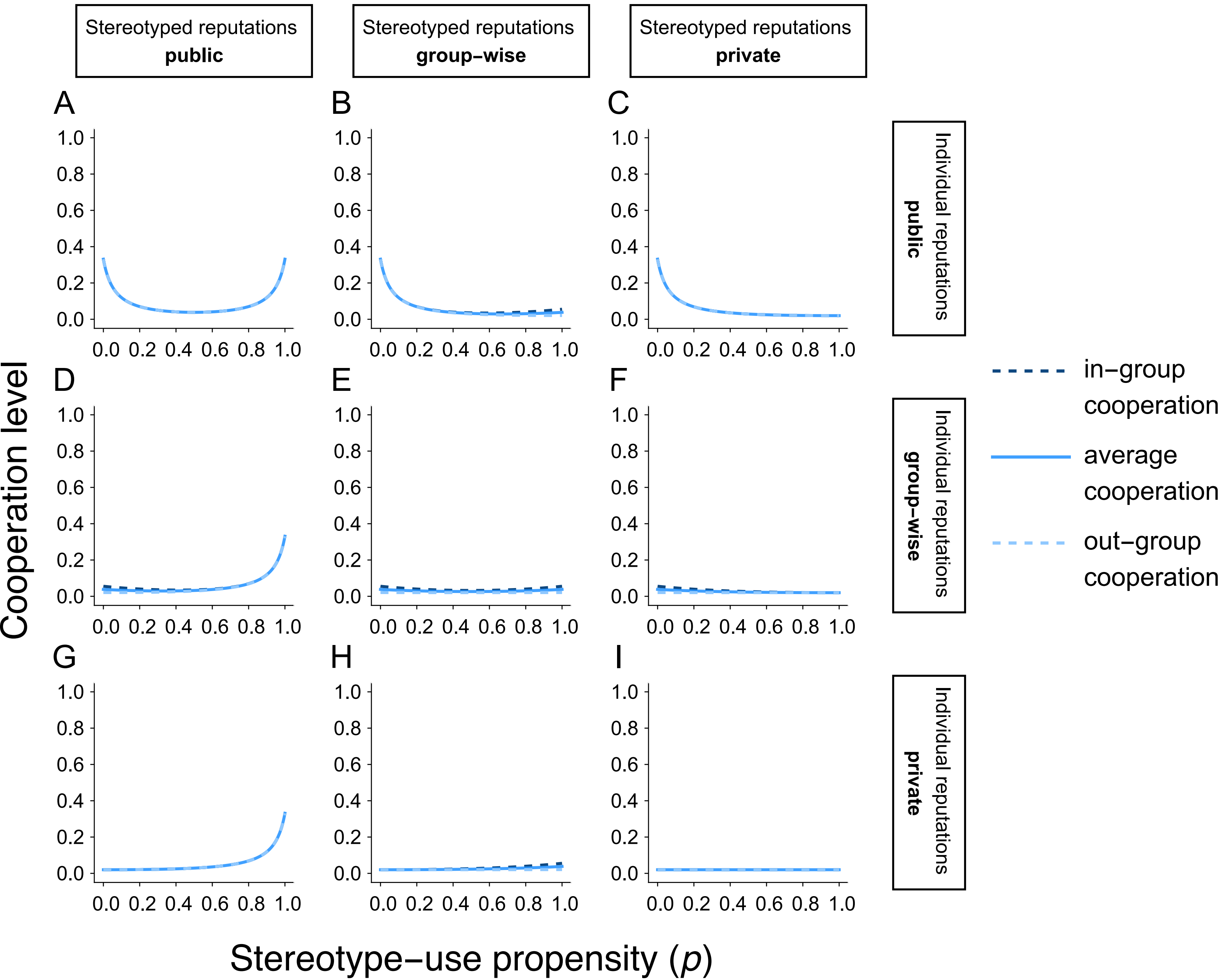

Stereotyped reputations public
Stereotyped reputations group–wise
Stereotyped reputations private
Individual reputations public
Individual reputations group–wise
Individual reputations private
A
B
C
D
E
F
G
H
I
Cooperation level
Stereotype–use propensity (p)
in–group cooperation
average cooperation
out–group cooperation
0.0
0.2
0.4
0.6
0.8
1.0
