# Supplementary material for: When do stereotypes undermine indirect reciprocity?
Source: PLoS Comput Biol. 2024 Mar 1;20(3):e1011862. doi: 10.1371/journal.pcbi.1011862 (PMC10906830; doi:10.1371/journal.pcbi.1011862)
Supplement: S3 Fig — As in Fig 2, but under the Scoring norm. (PDF) [file pcbi.1011862.s005.pdf]

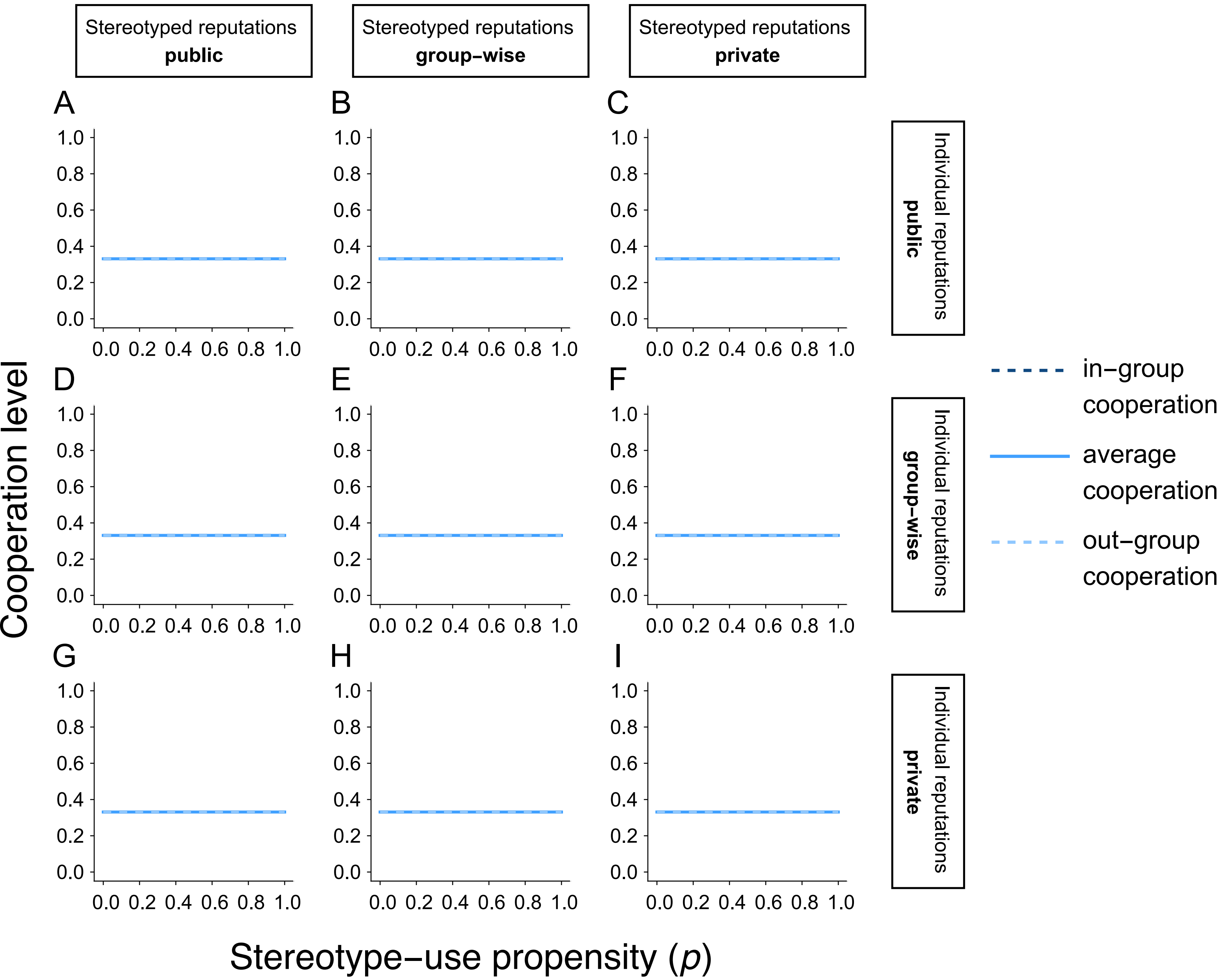
Stereotyped reputations
public
Stereotyped reputations
group−wise
Stereotyped reputations
private
Individual reputations
public
Individual reputations
group−wise
Individual reputations
private
A
B
C
D
E
F
G
H
I
Cooperation level
Stereotype−use propensity (p)
in−group cooperation
average cooperation
out−group cooperation
0.0
0.2
0.4
0.6
0.8
1.0
