# Supplementary material for: When do stereotypes undermine indirect reciprocity?
Source: PLoS Comput Biol. 2024 Mar 1;20(3):e1011862. doi: 10.1371/journal.pcbi.1011862 (PMC10906830; doi:10.1371/journal.pcbi.1011862)
Supplement: S7 Fig — We show the number and type of dynamically attractive values of p under the Stern Judging norm as a function of benefit of cooperation (b) and access cost for individual reputations (η). Individuals are distributed across two groups of equal size (K = 2, ν1 = ν2 = 0.5). Each panel shows a combination of monitoring systems for individual (rows) and stereotyped (columns) reputations. Light gray means stereotype use does not spread by adaptive dynamics (p = 0 in the only stable outcome). Hues of purple mean stereotype use will spread (p* > 0 is the only stable outcome). Hues of orange mean bistability (p = 0 and p* > 0 are both stable outcomes), i.e., stereotype use may spread depending on initial conditions. Parameters: c = 1, ue = ua = 0.02. Decreasing the benefit b of cooperation promotes stereotyping: A lower value of b increases the relative cost of accessing individual reputations, thus making stereotypes more beneficial. As a result, given a fixed η, decreasing b shifts the system from a regime that does not support stereotyping (light gray regions in A–F) through bistable regimes (light and dark orange regions in A–F), to regimes with a single attractive point (light and dark purple regions in A–F) in which stereotyping persists in the population in the long term regardless of initial conditions. Although a small benefit of cooperation generally promotes stereotyping, there is one exception: under private individual reputations, the long-term outcome is independent of b (G–I). Individuals gain a benefit b when donors view them as having good reputations and, therefore, cooperate with them (Model). Under private monitoring, invader and resident individuals have identical individual reputations on average, because two private observers’ assessments are uncorrelated. As a result, for any value of b, residents and invaders receive equal amounts of cooperation. Therefore, changing b has no impact on their relative fitness and, consequently, on the long-term outcomes [file pcbi.1011862.s009.pdf]

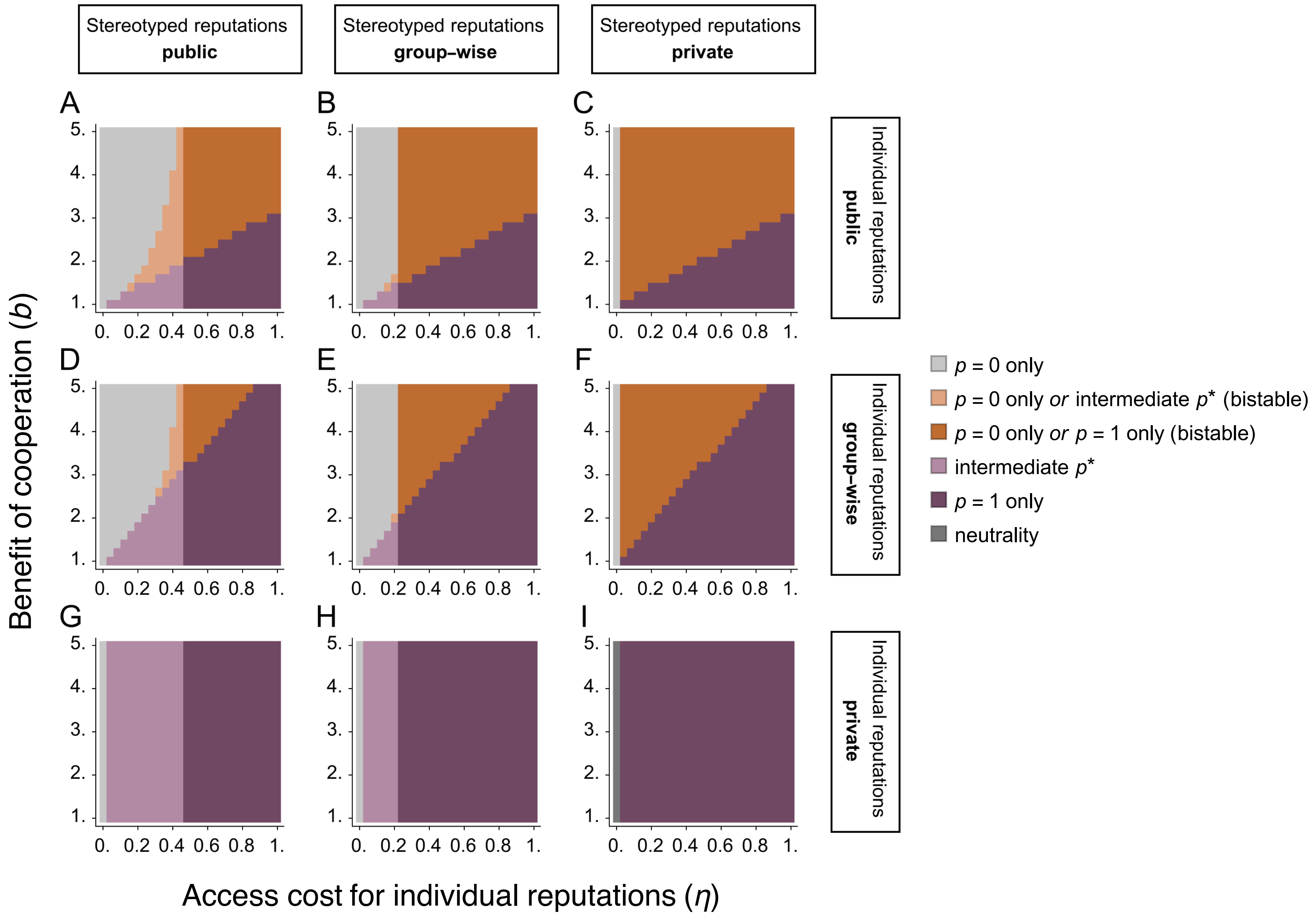

Stereotyped reputations public
Stereotyped reputations group–wise
Stereotyped reputations private
Individual reputations public
Individual reputations group–wise
Individual reputations private
A
B
C
D
E
F
G
H
I
Benefit of cooperation (b)
Access cost for individual reputations (η)
p = 0 only
p = 0 only or intermediate p* (bistable)
p = 0 only or p = 1 only (bistable)
intermediate p*
p = 1 only
neutrality
